# Organosilicon cluster goes ferroelectric

**DOI:** 10.1093/nsr/nwag243

**Published:** 2026-04-29

**Authors:** Huan-Huan Chen, Shu-Wen Xiong, Yan Qin, Xian-Jiang Song, Xiaomeng Liu, Zhanpeng Wang, Han-Yue Zhang, Ren-Gen Xiong

**Affiliations:** Ordered Matter Science Research Center, Nanchang University, Nanchang 330031, China; Ordered Matter Science Research Center, Nanchang University, Nanchang 330031, China; Ordered Matter Science Research Center, Nanchang University, Nanchang 330031, China; Ordered Matter Science Research Center, Nanchang University, Nanchang 330031, China; State Key Laboratory of Digital Medical Engineering, School of Biological Science and Medical Engineering, Southeast University, Nanjing 211189, China; State Key Laboratory of Digital Medical Engineering, School of Biological Science and Medical Engineering, Southeast University, Nanjing 211189, China; State Key Laboratory of Digital Medical Engineering, School of Biological Science and Medical Engineering, Southeast University, Nanjing 211189, China; Ordered Matter Science Research Center, Nanchang University, Nanchang 330031, China

**Keywords:** organosilicon cluster ferroelectric, molecular ferroelectric, structural phase transition, birefringence switching, symmetry breaking

## Abstract

Ferroelectrics and clusters have independently attracted significant attention as two important classes of functional materials. Bridging these two fields by introducing ferroelectricity into clusters offers a compelling strategy to overcome the intrinsic limitations of conventional small-molecule materials. However, cluster-based ferroelectrics remain exceedingly rare and challenging to realize. In this study, we report the construction of an organosilicon molecular cluster ferroelectric, aminopropyl isobutyl Si_8_O_12_ cluster (**1**), achieved by introducing distorted tetrahedral units with a polar –NH_2_ group. Crystal **1** undergoes a 3*F*1-type ferroelectric–ferroelastic phase transition at 328 K, accompanied by a distinct on/off switching behavior of optical birefringence. Comprehensive structural and physical characterizations confirm the coexistence of ferroelectricity and ferroelasticity. Remarkably, this multiferroic crystal demonstrates excellent thermal stability, high chemical robustness, a large number of polar axes, pronounced entropy change, and a low elastic modulus compared with previously reported organosilicon ferroelectric crystals. To the best of our knowledge, Crystal **1** is the first organosilicon cluster ferroelectric, establishing a new design paradigm for organosilicon and molecular ferroelectrics.

## Teaser

This study establishes a new organosilicon cluster multiferroic family through distorted tetrahedral units, enabling tunable structure, multi-channel functional responses, and enhanced thermal/chemical stability, thus establishing a promising paradigm for molecular ferroelectrics.

## INTRODUCTION

Ferroelectric materials, characterized by their switchable spontaneous polarization under external electric fields, have attracted considerable interest due to their fundamental importance in condensed matter physics and their potential for multifunctional device applications [1–5]. Among them, molecule-based ferroelectrics have emerged as a particularly attractive class because of their light weight, ease of solution processing, and mechanical flexibility [6–17]. Recently, organosilicon ferroelectric crystals have drawn interest as a promising subclass of molecular ferroelectrics, combining the versatile physicochemical properties of organosilicon compounds (for example, excellent biocompatibility, low surface energy, and low elastic modulus) with the functional electric responses inherent in ferroelectric materials [18–23]. Despite the existence of chemical design strategies for molecular ferroelectrics [24–26], the number of reported organosilicon ferroelectrics remains quite limited, and all such examples are organic small-molecule crystals. Furthermore, these materials usually suffer from low melting points, narrow operating temperature windows, and chemical instability, which collectively limit their practical applicability.

Clusters, composed of a finite number of bonded atoms or molecules, have attracted considerable interest owing to their unique structures and versatile applications, such as photonics, catalysis, nanotechnology, and materials science [27–32]. The integration of cluster with ferroelectricity provides a promising strategy for molecular ferroelectric systems, enabling externally controllable, ultrahigh-density, and low-power nonvolatile memory devices capable of operation under extreme conditions [4,33–36]. However, molecular clusters exhibiting intrinsic ferroelectricity remain extremely rare [34,37], with only a few examples reported to date, including a potential ferroelectric-like carbon-based cluster, Gd@C_82_ [34], and a spherical carborane-based molecular ferroelectric [37]. Among cluster systems, organosilicon molecular clusters are particularly attractive because of their chemical tunability, excellent thermal stability, and compatibility with both organic and inorganic systems [38–43]. Realizing ferroelectricity in organosilicon clusters could potentially address the drawbacks of small-molecule organosilicon ferroelectrics while also broadening their multifunctional applications [4,33–36]. Yet, the high structural symmetry of most organosilicon clusters fundamentally hinders the emergence of polar structures, making ferroelectricity in this class of materials still unexplored.

**Scheme 1.**
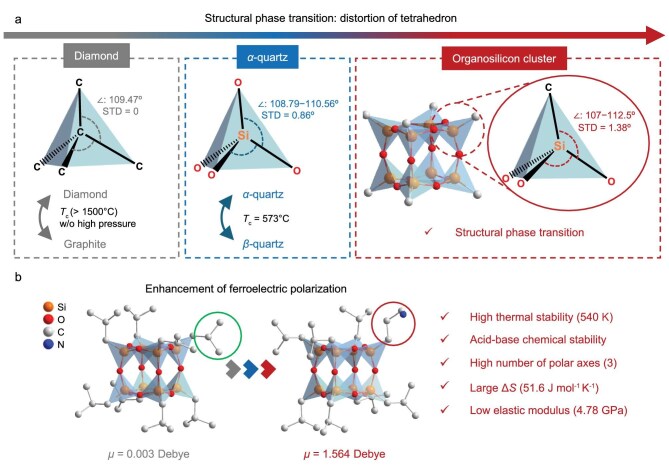
Chemical design for organosilicon molecular cluster ferroelectrics. (a) Distortion of the RSiO_1.5_ tetrahedral unit in the Si_8_O_12_ cage may promote a structural phase transition. (b) The introduction of an amino group enhances ferroelectric polarization. Angles in (a) represent all bond angles within each tetrahedron.

Structural phase transitions are crucial for generating ferroelectricity and are strongly structure-dependent. For example, diamond, with its perfect tetrahedral structure, requires extreme conditions for phase transition, whereas *α*-quartz, due to its structural distortion, undergoes displacive phase transition at significantly lower temperatures (Scheme 1a) [44–47]. Inspired by this tendency, we explored the cage-like Si_8_O_12_ cluster, composed of eight RSiO_1.5_ tetrahedral units, which inherently exhibits great geometric distortion. In this work, we introduced a polar –NH_2_ group into an isobutyl-substituted Si_8_O_12_ cluster to lower molecular symmetry and enhance the molecular dipole moment, thereby creating favorable conditions for ferroelectricity (Scheme 1b). The resulting aminopropylisobutyl Si_8_O_12_ cluster crystal (**1**) represents the first organosilicon molecular cluster multiferroic. It undergoes a 3*F*1-type ferroelectric–ferroelastic phase transition between the polar point groups *P*1 and *R*3 at 328 K, accompanied by a birefringence switching behavior. Notably, crystal **1** also exhibits favorable optical properties, including a broad optical transmission window and a wide operational range for second-harmonic generation (SHG). More interestingly, this cluster-based ferroelectric crystal exhibits unique combination of properties, including high thermal stability (540 K), chemical stability under both acidic and basic conditions, the highest number of polar axes (3), a large entropy change (51.6 J mol^−1^ K^−1^), and a low elastic modulus (4.78 GPa) (Scheme 1b), surpassing previously reported small-molecule organosilicon ferroelectric crystals (Table 1). To the best of our knowledge, this work reports the first demonstration of ferroelectricity in an organosilicon molecular cluster, establishing the Si_8_O_12_ cluster as a new platform for stable, high-performance organosilicon ferroelectrics.

**Table 1.** Comparisons between the thermal stability, number of polar axes, entropy change, and elastic modulus of compound **1** with other reported organosilicon ferroelectric crystals.

Material	Thermal stability (K)	Number of polar axes	Entropy change, Δ*S* (J mol^−1^ K^−1^)	Modulus, *E* (GPa)	Experimental (theoretical) spontaneous polarization, *P*_s_ (μC/cm^2^)	Ref.
**Compound 1**	**540**	**3**	**51.6**	**4.78**	**0.26 (0.42)**	**This work**
(*R/S*)-BINOL-DIPASi^[^^a^^]^	458	2	1.0/3.8	7.7/6.8	1.6/1.7	[18]
D-*chiro*-inositol-SiMe_3_^[^^b^^]^	355	N/A	N/A	5.04	1.4	[19]
*R/S*-SEA-Si^[^^c^^]^	378	N/A	N/A	N/A	(1.17)	[20]
4-AA	403	1	4.8	5.94	0.1 (0.1)	[21]
*R/S-*[TBSQ][TCNQ]^[^^d^^]^	480	1	6.76/6.78	N/A	1.4/1.3	[22]
TFPES^[^^e^^]^	550	N/A	15.1	0.41	N/A	[23]

^[a]^No theoretical spontaneous polarization values were reported. ^[b]^No number of polar axes, structural phase transition and theoretical spontaneous polarization values were reported. ^[c]^No number of polar axes, structural phase transition, modulus data and measured spontaneous polarization value were reported. ^[d]^No modulus data was reported. ^[e]^No number of polar axes and spontaneous polarization value were reported.

## RESULTS AND DISCUSSION

To induce a structural phase transition, we first introduced the isobutyl groups into the Si_8_O_12_ cluster. The resultant isobutyl Si_8_O_12_ cluster (**2**) exhibited a structural phase transition and was found to crystallize in the polar space group *P*1 at room temperature. Although crystal **2** had been previously reported to crystallize in the centrosymmetric space group *P*${\mathrm{\!\!\!\bar{\,\,\,1}}}$, we observed a clear SHG response, suggesting a non-centrosymmetric crystal structure (Fig. S1). According to the principle proposed by Franz Ernst Neumann, which states that the symmetry of physical properties must be higher than or equal to the point group symmetry of the crystal, this SHG response suggests that crystal **2** likely crystallizes in the polar space group *P*1, rather than *P*$\!\!\!\bar{\,\,\,1}$. Despite exhibiting a structural phase transition and a polar structure (Figs S2–S5 and Table S1), the molecular dipole moment of crystal **2** is insufficient to give rise to detectable ferroelectric behavior. Herein, to further lower molecular symmetry and enhance the molecular dipole moment, we introduced a single –NH_2_ group while retaining the other seven isobutyl chains, and eventually obtained crystal **1**.

**Figure 1.**
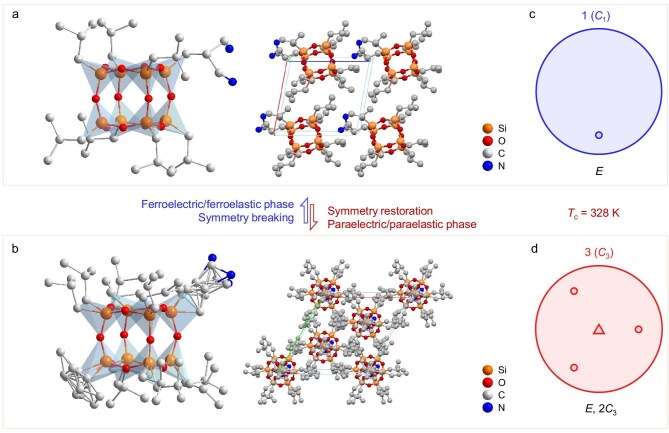
Asymmetric unit (left) and packing view of unit cell (right) in the (a) RTP and (b) HTP. Hydrogen atoms were omitted for clarity, and complete figures are available in the Supporting Information. Transformation of the point group between (c) ferroelectric phase (RTP) and (d) paraelectric phase (HTP).

**Figure 2.**
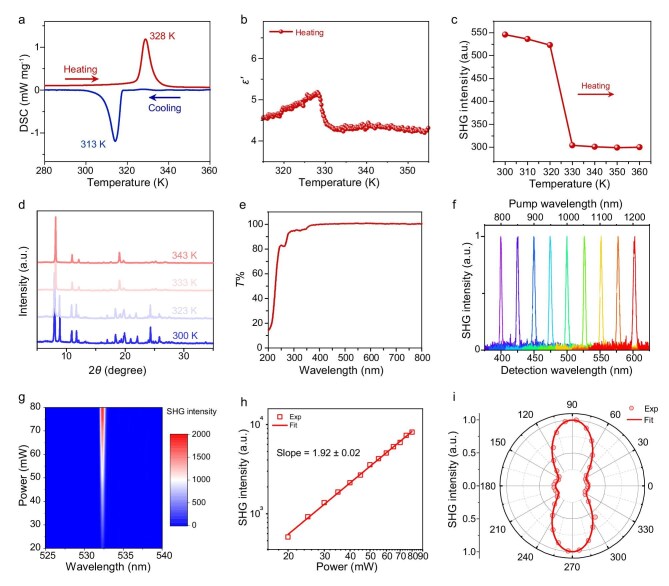
(a) DSC curves of compound **1** in a heating and cooling cycle. (b) Real part (*ε′*) of the complex dielectric constant as a function of temperature of compound **1** during heating. (c) Temperature-dependent SHG intensity of compound **1** during heating. (d) Variable-temperature powder XRD measurements of compound **1**. (e) UV-visible transmittance spectrum of compound **1**. (f) Wavelength-dependent SHG measurement of compound **1**. (g) Power-dependent SHG measurement of compound **1**. (h) Log plot of SHG intensity versus incident power with a slope of 1.92 ± 0.02. (i) SHG intensity as a function of polarization angle, controlled via half-wave plate.

Colorless crystals of compound **1** were obtained by slow evaporation of an ethyl acetate/petroleum ether solution (*v*/*v* = 1:1). The phase purity of compound **1** was confirmed by powder X-ray diffraction (XRD) measurement (Fig. S6). Thermogravimetric analysis (TGA) result indicated that compound **1** exhibited high thermal stability, with an onset decomposition temperature of ∼540 K, far beyond that of most small-molecule organosilicon ferroelectric crystals (Fig. S7 and Table 1) [18–23]. Single crystal XRD analysis revealed that compound **1** crystallizes in the polar space group *P*1 at room temperature (Fig. 1a), with the cell parameters *a* = 10.1134(2) Å, *b* = 11.1379(1) Å, *c* = 11.1621(1) Å, and *β* = 100.347(1)° (Table S2). As shown in Fig. 1a, the asymmetric unit contains one independent molecule, with the amino group exhibiting disorder. The differential scanning calorimetry (DSC) analysis revealed a reversible phase transition at 328 K (Fig. 2a). To gain insight into the structural factors associated with this phase transition, a detailed structural analysis was performed. As revealed by single-crystal X-ray analysis, diamond possesses an ideal C–C–C tetrahedral structure, and the graphite-to-diamond phase transition requires extreme conditions, whereas SiO_2_ contains slightly distorted O–Si–O tetrahedra (standard deviation of 0.86°) and undergoes a phase transition at 573°C, suggesting that the degree of tetrahedral distortion may influence structural phase transitions. In this context, the SiRO_1.5_ tetrahedra in compound **1** exhibited more pronounced distortion, with bond angles ranging from 107.0° to 112.5° and a standard deviation of 1.38° (Scheme 1). Consistent with this observation, compound **1** undergoes a thermally induced phase transition at 328 K, suggesting that the distortion of the RSiO_1.5_ tetrahedral units may facilitate the structural rearrangement. The phase below 328 K is defined as the room-temperature phase (RTP), while the phase above 328 K is defined as the high-temperature phase (HTP). Compound **1** experienced an order–disorder transition and exhibited a large entropy change (Δ*S*) of 51.6 J mol^−1^ K^−1^, which is characteristic of an order–disorder-type phase transition. To the best of our knowledge, this value significantly exceeds that of all reported small-molecule organosilicon ferroelectrics [18–23], including the previous record-holder, tetrakis(4-fluorophenylethynyl)silane (TFPES) [23], which has a Δ*S* of 15.1 J mol^−1^ K^−1^ (Table 1). This large Δ*S* suggests that compound **1** may be a promising candidate for applications in solid-state refrigeration, thermal energy conversion, and energy harvesting.

Compound **1** undergoes a thermally induced phase transition at 328 K. Upon heating, the HTP of crystal **1** crystallizes in the polar space group *R*3 with cell parameters *a* = 16.1357(12) Å, *b* = 16.1357(12) Å, *c* = 17.1112(17) Å, and *β* = 90° (Table S2). The tetrahedral units exhibited greater distortion, and both the amino group and the alkyl chain exhibited greater disorder (Fig. 1b), further confirming the order–disorder phase transition of **1**. Moreover, a significant change in the bond angle of the RSiO_1.5_ tetrahedral units occurs during the phase transition. This phase transition involves a symmetry breaking from a high-symmetry trigonal phase (*R*3) to a low-symmetry triclinic phase (*P*1). According to Aizu notation, this 3*F*1 phase transition can give rise to a reversible ferroelectric and ferroelastic transition. The symmetry breaking of **1** from identity operation (*E*) and 2*C*_3_ in phase *R*3 (point group 3) to only the *E* in phase *P*1 (point group 1) indicates multiaxial ferroelectricity in **1**, whereas most reported organosilicon ferroelectrics are uniaxial (Fig. 1c and d, and Table 1) [18–23].

Comprehensive characterization was further employed to capture the phase transition behavior of compound **1**. As depicted in Fig. 2b, temperature-dependent measurement of the real part (*ε*′) of the complex dielectric constant (*ε* = *ε*′ − *iε*″, where *ε*″ is the imaginary part) revealed a peak-shaped anomaly near the phase transition temperature (*T*_c_) upon heating. Similarly, the step-like anomaly observed in the SHG [48] signal between 320 and 330 K during cooling strongly supports a change in symmetry between the RTP and HTP (Fig. 2c). Moreover, the powder XRD measurement indicated that as the temperature increased above the *T*_c_, changes were observed in the powder XRD patterns, including the disappearance and merging of most diffraction peaks, indicating the appearance of a structural phase transition (Fig. 2d). All the results are consistent with DSC data and further verify the phase transition behavior of compound **1**.

We further investigated the optical properties of compound **1**. The UV-visible transmittance measurement revealed that compound **1** exhibits a broad optical transmission window spanning from the ultraviolet to the visible region, indicating good optical transparency (Fig. 2e). In addition, wavelength-dependent SHG measurement indicated that compound **1** also possesses a wide operational range, suggesting its favorable second-order nonlinear optical properties (Fig. 2f). To elucidate the mechanism of SHG generation in compound **1**, power-dependent SHG spectroscopy was further employed (Fig. 2g and h). A log–log plot of the SHG intensity and pump power revealed a near-quadratic dependence with a fitted slope of 1.92 ± 0.02, confirming that the detected signal originates from a second-order nonlinear optical process. Also, the polarization-dependent SHG measurements revealed pronounced anisotropy with a characteristic dipole-like pattern, and the polarization ratio *ρ* of compound **1** is calculated to be 0.84, revealing strong polarization anisotropy in its SHG response (Fig. 2i). The wide transparency window, broadband SHG response, and pronounced polarization anisotropy indicate that compound **1** possesses second-order nonlinear optical properties, making it a promising candidate for nonlinear optical materials.

**Figure 3.**
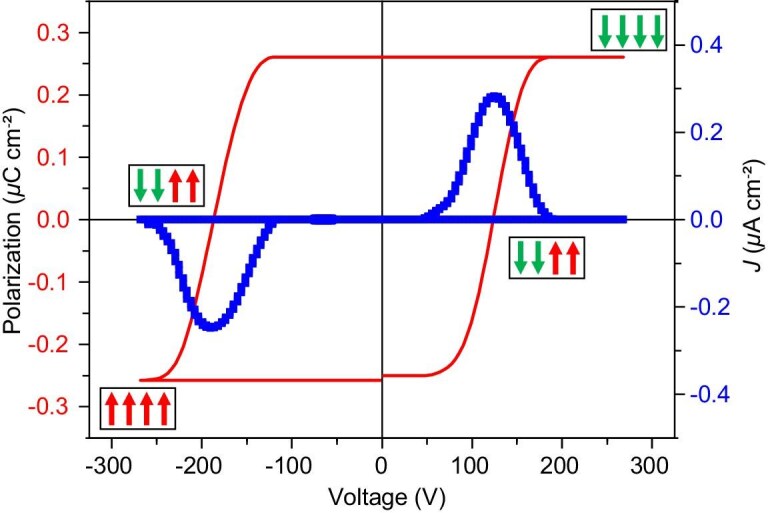
*P-V* hysteresis loop measured on the thin film of compound **1**. Arrows are the schematic illustration of molecular dipole moments.

The ferroelectricity of compound **1** was directly verified by measuring polarization–voltage (*P-V*) hysteresis loops on its crystalline thin film using the double-wave method (Fig. 3). Two typical opposite peaks can be observed in the current density–voltage (*J-V*) curve, indicating two stable states with opposite polarizations. From the *J-V* curve, the well-shaped *P-V* hysteresis loop was further obtained with spontaneous polarization (*P*_s_) of about 0.26 μC/cm^2^. Figure S8 exhibits the *P-V* hysteresis loops under different testing periods and the polarization intensity almost remain unchanged while the coercive voltage slightly decreases. The cycle stability of the saturation polarization has been investigated, showing no significant degradation after multiple cycles. To further evaluate the ferroelectric polarization of compound **1**, we calculated the vector sum of the corresponding molecular dipole moment in the unit cell. The estimated *P*_s_ value is approximately 0.42 μC/cm^2^, which is consistent with the typical range reported for organosilicon ferroelectrics (Table 1). The relatively lower experimental *P*_s_ may result from multiple factors. Only a fraction of the molecular dipoles may be effectively switchable under the applied electric field, while experimental factors such as the quality of the thin film may further contribute to the reduced polarization.

**Figure 4.**
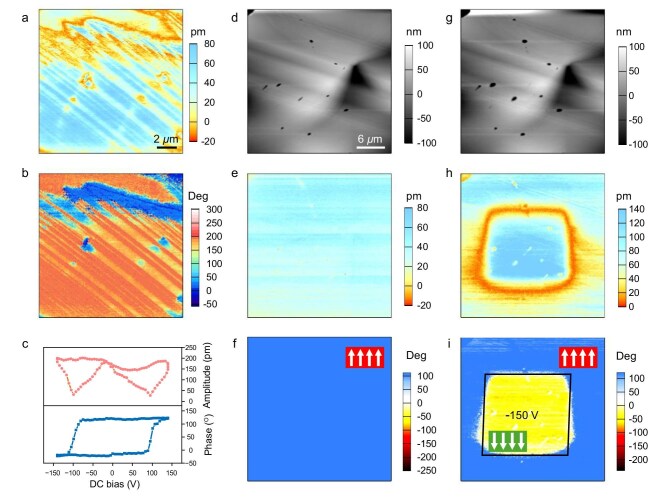
PFM (a) phase and (b) amplitude images on the thin film of compound **1**. (c) Local PFM hysteresis loops obtained by plotting the phase and amplitude signals as functions of the tip voltage for a selected point. Domain switching was observed on the thin film of compound **1**, with each column displaying the topographic image (top), PFM amplitude image (middle), and phase image (bottom) in sequence. The initial state of the film is shown in panels (d–f), while panels (g–i) represent the state after switching, achieved by applying a point poling bias of −150 V at the central position. Arrows are the schematic illustration of molecular dipole moments.

As a well-established technique, piezoresponse force microscopy (PFM) enables both visualization of static domain structures and controlled polarization switching at the micrometer or nanometer scale, offering advantages such as relatively high spatial resolution, non-destructive nature, and simplicity of sample preparation [49,50]. Due to its capability to resolve local domain structures and switching behavior clearly, PFM was employed to investigate the ferroelectric properties of compound **1** at the microscopic level. In the thin film of compound **1**, well-defined ferroelectric domains were clearly observed (Fig. 4a and b). The phase image shows distinct contrast, and the amplitude image reveals clear domain boundaries that correlate well with phase variations while showing no association with surface topography. We further applied the switching spectroscopy piezoresponse force microscopy hysteresis method to verify the ferroelectricity of compound **1**. Figure 4c presents a typical butterfly-shaped amplitude*-*voltage loop along with a corresponding phase hysteresis loop, indicating the switching and hysteresis behaviors of polarization, which are obtained in the field-off period. To further support the ferroelectricity of compound **1**, local switching of domains was conducted on the crystalline thin films of compound **1**. Typically, a characteristic polarization switching process consists of four stages: (i) domain nucleation, (ii) forward extension, (iii) lateral expansion, and (iv) domain merging. Figure 4d and f displays the initial domain configuration of the sample. After applying −150 V to the center area of an initially single-domain region. A new domain has been generated with a clear domain wall and distinct phase contrast (Fig. 4g–i). This reversible domain evolution confirms robust ferroelectric switching behavior. Moreover, surface topography images acquired before and after switching cycles showed no detectable damage or variation, effectively excluding any contribution of surface morphology to the PFM response. Together, these results provide compelling evidence for the ferroelectric nature of **1.** The discovery of the organosilicon cluster ferroelectric **1** suggests that combining intrinsic structural distortion with polar groups may provide a feasible strategy for constructing cluster-based ferroelectrics. This concept could potentially be extended to both organosilicon molecular clusters and metal-based cluster systems. In this regard, preserving the inherent distortions of the cluster framework while introducing polar groups to modulate dipole moments and intermolecular interactions may be important for the rational design of new cluster-based ferroelectrics.

**Figure 5.**
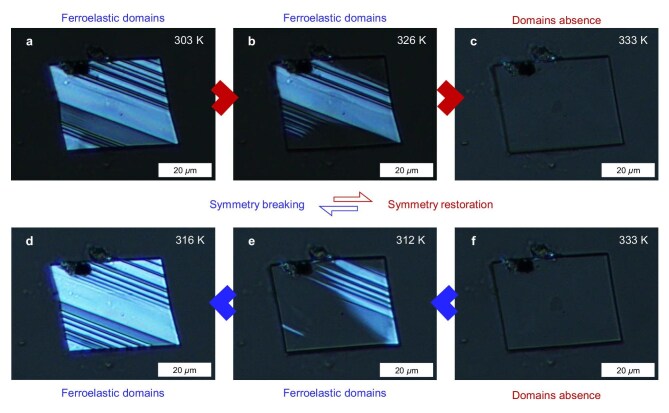
The evolution of ferroelastic domains of **1** during the ferroelastic phase transition. Optical images under an orthogonal polarizing microscope during the heating process (a–c) and cooling process (d–f).

From the perspective of symmetry change, compound **1** undergoes a reversible 3*F*1-type ferroelectric–ferroelastic phase transition, according to Aizu notation. The ferroelastic behavior of compound **1** was further confirmed by the evolution of ferroelastic domains (Fig. 5). Specifically, a ferroelastic domain is defined as a microscopic region with the same spontaneous strain orientation state within a ferroelastic material, serving as direct evidence of ferroelasticity. Such ferroelastic domains can be observed using polarized light microscopy, because domains with different orientations exhibit distinct birefringence properties, resulting in alternating light and dark patterns. The evolution of ferroelastic domains during the paraelastic-to-ferroelastic phase transition is a characteristic feature of ferroelastic crystals. As shown in Fig. 5, distinct stripe-like domains are observed in the RTP of compound **1** crystals, and these domain patterns remain stable in the RTP. When the temperature rises to above 328 K, the ferroelastic domains disappear rapidly, and the material enters the paraelastic phase (Fig. 5a–c). During subsequent heating/cooling of the crystalline thin film, the ferroelastic domains reappear and vanish reversibly, confirming the ferroelastic nature of compound **1** (Fig. 5d–f). Unlike traditional ferroelastic phase transitions that are related to lattice distortion [51,52], compound **1** undergoes a ferroelastic/ferroelectric type phase transition, indicating that this ferroelastic phase transition is also accompanied by symmetry breaking and restoration. In addition, ferroelastic domain switching following a clockwise rotation was observed, providing further evidence for the ferroelastic behavior of the material (Fig. S9). The spontaneous strain components were calculated from the lattice parameters. The calculated spontaneous strain reaches a relatively large value of 0.097, which is ∼10 times greater than that of the well-known Rochelle salt (0.9133 × 10^−2^ at 274 K) [53]. The detailed calculation procedure is provided in the Supporting Information [54]. Interestingly, compound **1** crystal exhibits a reversible macroscopic shape change during its thermally induced phase transition (Fig. S11) [55–57]. Upon heating above the Curie point of 328 K, the crystal undergoes a macroscopic elongation of ∼10% in the length of the crystal. This deformation is fully reversible upon cooling through the transition. This robust actuation behavior suggests potential applications in thermal actuators and expansion compensators.

We further investigated the evolution of birefringence during the phase transition. In birefringent materials, the ordinary and extraordinary light propagate with different refractive indices (*N_o_* and *N_e_*), which leads to an optical path difference (*R*) when light passes through the sample. Birefringent (Δ*n*) is defined as the difference between the refractive indices of the extraordinary and ordinary light (Δ*n* = |*N_e_ − N_o_*|). In our experiment, optical path difference *R* was determined using an orthogonal polarized optical microscope equipped with a Berek compensator with a wavelength of 550 nm. The thickness of the thin film was independently measured by atomic force microscopy and was ∼3.3 μm. The birefringence was calculated according to the following equation:


(1) \begin{eqnarray*}
\Delta n = |N_e - {N}_o| = R/d.
\end{eqnarray*}

**Figure 6.**
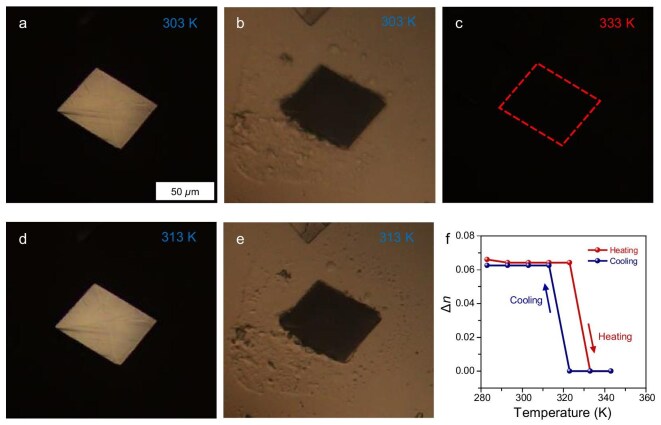
Temperature stimuli-induced on/off switchable birefringence of **1**. (a–c) Optical images of **1** during the heating process under an orthogonal polarizing microscope at 303 K (a) and 333 K (c). (b) **1** achieving complete extinction by a Berek compensator at 303 K. (c–e) Optical images of **1** during the cooling process under an orthogonal polarizing microscope at 333 K (c) and 313 K (d). (e) **1** achieving complete extinction by a Berek compensator at 313 K. (f) The temperature dependence of birefringence during the heating and cooling process.

In principle, the paraelectric–paraelastic phase of **1** belongs to the trigonal crystal system, which is isotropic without birefringence along the optical axis, whereas the ferroelectric–ferroelastic phase adopts a triclinic structure and is optically biaxial with pronounced birefringence. Consequently, this phase transition enables reversible on/off switching of birefringence between the birefringence-active triclinic phase and the birefringence-inactive trigonal phase. At room temperature, **1** exhibits a clear birefringent response with a birefringence of ∼0.065 (Fig. 6a, b, and f). Upon further heating up to 333 K, complete extinction is observed and the birefringence decreases to zero, corresponding to the off state (Fig. 6a–c). Besides, upon further cooling to 313 K, a clear birefringent response is observed again, and the birefringence increases to a stable state corresponding to the on state (Fig. 6c–f). This reversible birefringence switching is consistent with the thermally induced 3*F*1-type ferroelectric–ferroelastic transition, indicating potential applications in smart optical switches and sensors [52,58–60].

Mechanical properties of compound **1** crystalline films were further assessed by the nanoindentation method (Fig. S12). The elastic modulus (*E*) and hardness (*H*) values were calculated and exhibit an *E* of 4.78 GPa and an *H* of 100.1 MPa, respectively. These values present favorable compliance relative to typical organic crystals with an average *E* and *H* of 12.05 GPa and 500.0 MPa [61], respectively, indicating good mechanical properties. Notably, compound **1** exhibited exceptional chemical stability. To demonstrate it, we immersed its powder and 4-AA, a representative small-molecule organosilicon ferroelectric in aqueous HCl (1 M) and NaOH (1 M) solutions. 4-AA dissolved completely in acid and partially dissolved in base solution, the latter accompanied by a color change, indicating its degradation under both acid and basic conditions. In contrast, the morphology of compound **1** remained unchanged (Fig. S13). Its chemical stability was further confirmed by the powder XRD pattern of the solid recovered after solvent exposure (Fig. S14). These findings collectively underscore the exceptional chemical stability of **1** compared to other small-molecule organosilicon ferroelectrics.

## CONCLUSION

To conclude, we reported the first organosilicon molecular cluster multiferroic crystal **1**, constructed via introducing distorted tetrahedra and –NH_2_ group. This crystal experiences a 3*F*1-type full ferroelectric–ferroelastic phase transition at 328 K. The order–disorder transition of the compound **1** molecule in the lattice is responsible for the phase transition. PFM and *P*-*V* hysteresis loop measurements confirmed its ferroelectricity, while the evolution of ferroelastic domains verified its ferroelasticity. The crystal **1** also presents a birefringence switching behavior associated with the phase transition. Besides, crystal **1** exhibits a broad optical transmission window and a wide operational range for SHG. The integrated properties of reversible thermoelastic transformation, high thermal stability, exceptional chemical stability, the highest number of polar axes, large entropy change, and low elastic modulus collectively make **1** a promising candidate for applications in sensors, actuators, soft robotics, and so on. This is the first discovery of an organosilicon molecular cluster multiferroic crystal. This work highlights the potential of the Si_8_O_12_ cluster paradigm in designing chemically and thermally stable organosilicon ferroelectrics and bridges small molecular and molecular cluster ferroelectrics.

## METHODS

### Crystal growth

AR (analytical regent) pure aminopropylisobutyl Si_8_O_12_ (**1**) and isobutyl Si_8_O_12_ (**2**) were purchased from Adamas-beta (CAS number: 444 315–15–5 and 221326–46–1). All reagents and solvents in the syntheses were of reagent grade and used without further purification. The colorless crystals of **1** and **2** were easily obtained by slow evaporation of its ethyl acetate/petroleum ether (1:1) mixture at room temperature.

### Thin film preparation

The thin films were prepared through a drop-casting method. An amount of 12 mg of the **1** crystals was dissolved in 0.6 mL ethyl acetate to prepare a precursor solution, 20 μL of which was then spread on a clean indium-doped tin oxide glass substrate. The planar and compact thin film was obtained after annealing at 347 K for 0.5 h.

### Measurements

Methods of DSC, TGA, dielectric, SHG, single-crystal XRD, powder XRD, ferroelectric, and PFM measurements were described in the supporting information. The crystal structures generated in this study have been deposited in the Cambridge Crystallographic Data Centre under accession codes CCDC 2494370–2494371 for **1**, and 2498131–2498133 for **2**, and can be obtained free of charge via https://www.ccdc.cam.ac.uk/structures/.

## Supplementary Material

nwag243_Supplemental_Files
