# Supplementary material for: Organosilicon cluster goes ferroelectric
Source: Natl Sci Rev. 2026 Apr 29;13(14):nwag243. doi: 10.1093/nsr/nwag243 (PMC13411271; doi:10.1093/nsr/nwag243)
Supplement: nwag243_Supplemental_Files [file nwag243_supplemental_files.zip › cif files/checkcif_compound-1_300k.pdf]

No syntax errors found.
Please wait while processing ....

CIF dictionary
Interpreting this report

Datablock: xsw-250807-2-300k-sphere_auto

Bond precision: C-C = 0.0057 A Wavelength=1.54184

Cell: a=10.1134(2) b=11.1379(1) c=11.1621(1)
alpha=93.844(1) beta=100.347(1) gamma=90.922(1)

Temperature: 300 K

	Calculated	Reported
Volume	1233.59(3)	1233.59(3)
Space group	P 1	P 1
Hall group	P 1	P 1
Moiety formula	C31 H71 N O12 Si8	C31 H71 N O12 Si8
Sum formula	C31 H71 N O12 Si8	C31 H71 N O12 Si8
Mr	874.61	874.60
Dx,g cm-3	1.177	1.177
Z	1	1
Mu (mm-1)	2.466	2.466
F000	472.0	472.0
F000'	475.17	
h,k,lmax	12,13,13	12,13,13
Nref	10018[5009]	8648
Tmin,Tmax	0.781,0.781	0.726,1.000
Tmin'	0.781	

Correction method= # Reported T Limits: Tmin=0.726 Tmax=1.000
AbsCorr = MULTI-SCAN

Data completeness= 1.73/0.86 Theta(max)= 74.102

R(reflections)= 0.0828(6878) wR2(reflections)= 0.2838(8648)

S = 1.219 Npar= 485

The following ALERTS were generated. Each ALERT has the format
test-name_ALERT_alert-type_alert-level.
Click on the hyperlinks for more details of the test.

Alert level C

```
STRVA01_ALERT_4_C         Flack test results are ambiguous.
           From the CIF: _refine_ls_abs_structure_Flack     0.464
           From the CIF: _refine_ls_abs_structure_Flack_su     0.019
PLAT084_ALERT_3_C High wR2 Value (i.e. > 0.25) ...................       0.28 Report
PLAT220_ALERT_3_C NonSolvent    Resd 1   C    Ueq(max)/Ueq(min) Range      3.3 Ratio
PLAT222_ALERT_3_C NonSolvent Resd 1   H   Uiso(max)/Uiso(min) Range      4.1 Ratio
PLAT230_ALERT_2_C Hirshfeld Test Diff for    Si3      --O2        .        5.7 s.u.
And 3 other PLAT230 Alerts
More ...
PLAT234_ALERT_4_C Large Hirshfeld Difference C1       --C24       .       0.16 Ang.
And 8 other PLAT234 Alerts
More ...
PLAT241_ALERT_2_C High    'MainMol' Ueq as Compared to Neighbors of        O2 Check
And 3 other PLAT241 Alerts
More ...
PLAT242_ALERT_2_C Low     'MainMol' Ueq as Compared to Neighbors of       Si1 Check
And 12 other PLAT242 Alerts
More ...
PLAT260_ALERT_2_C Large Average Ueq of Residue Including       Si1      0.127 Check
PLAT340_ALERT_3_C Low Bond Precision on  C-C Bonds ...............    0.00568 Ang.
PLAT412_ALERT_2_C Short Intra XH3 .. XHn     H2        ..H28A      .       1.85 Ang.
                                                      x,y,z  =      1_555 Check
And 2 other PLAT412 Alerts
More ...
PLAT413_ALERT_2_C Short Inter XH3 .. XHn     H22A      ..H30A      .       2.04 Ang.
                                                   -1+x,y,z  =      1_455 Check
PLAT420_ALERT_2_C D-H Bond Without Acceptor  N10       --H10D      .     Please Check
And 3 other PLAT420 Alerts
More ...
PLAT911_ALERT_3_C Missing FCF Refl Between Thmin & STh/L=    0.600         48 Report
              -12  2  2,   8 -8  5,  -9 -8  6,   8 -7  6,  -9  1  6,  -9 -7  7,
               -9 -6  7, -10 -5  7,  -8 -7  8,  -9 -6  8,  -8 -6  8,  -9 -5  8,
               -8 -5  8, -10 -4  8,  -9 -4  8,  -8 -4  8, -10 -3  8,  -7 -8  9,
               -8 -7  9,  -7 -7  9,  -8 -6  9,  -7 -6  9,  -9 -5  9,  -8 -5  9,
```

```
              -7 -5  9,   -9 -4  9,   -8 -4  9,   -7 -4  9,   -9 -3  9,   -8 -3  9,
                  (  18 More Missing: see the .ckf listing file)
PLAT915_ALERT_3_C No Flack x Check Done: Low Friedel Pair Coverage         78 %
PLAT918_ALERT_3_C Reflection(s) with I(obs) much Smaller I(calc) .         10 Check
              -2  0 -3,    0 -3 -2,    3  0 -2,   -1  1 -1,   -2  3 -1,    2 -3  1,
               1 -1  1,   -3  0  1,    0  3  2,    2  0  3,
PLAT939_ALERT_3_C Large Value of Not (SHELXL) Weight Optimized S .      12.36 Check
PLAT987_ALERT_1_C The Flack x is >> 0 -  Do a BASF/TWIN Refinement      Please Check
```

Alert level G

```
PLAT002_ALERT_2_G Number of Distance or Angle Restraints on AtSite         44 Note
PLAT003_ALERT_2_G Number of Uiso or U(i,j)  Restrained non-H-Atoms         54 Report
PLAT007_ALERT_5_G Number of Unrefined Donor-H Atoms .............           4 Report
             H10D     H10E     H10F     H10G
PLAT012_ALERT_1_G N.O.K.    _shelx_res_checksum Found in CIF ......     Please Check
PLAT033_ALERT_4_G Flack x Value Deviates > 3.0 * Sigma from Zero .      0.464 Note
PLAT072_ALERT_2_G SHELXL First  Parameter in WGHT  Unusually Large       0.20 Report
PLAT111_ALERT_2_G ADDSYM Detects New (Pseudo) Centre of Symmetry .         92 %Fit
PLAT113_ALERT_2_G ADDSYM Suggests Possible Pseudo/New  Space-group        P-1 Check
             WARNING: Disordered Atoms Excluded from Analysis
             Check Model Parameter Symmetry for Reflection Data Support
PLAT154_ALERT_1_G The s.u.'s on the Cell Angles are Equal ..(Note)      0.001 Degree
PLAT172_ALERT_4_G The CIF-Embedded .res File Contains DFIX Records         15 Report
PLAT173_ALERT_4_G The CIF-Embedded .res File Contains DANG Records          6 Report
PLAT176_ALERT_4_G The CIF-Embedded .res File Contains SADI Records          4 Report
PLAT178_ALERT_4_G The CIF-Embedded .res File Contains SIMU Records          3 Report
PLAT186_ALERT_4_G The CIF-Embedded .res File Contains ISOR Records          1 Report
PLAT188_ALERT_3_G A Non-default SIMU Restraint Value has been used     0.0100 Report
And 2 other PLAT188 Alerts
More ...
PLAT191_ALERT_3_G A Non-default SADI Restraint Value has been used     0.0400 Report
PLAT299_ALERT_4_G Atom Site Occupancy Constrained at ............         0.5 Check
             N10      N10A     C9       C9A      H9AA     H9AB     H9A      H9B
             H10D     H10E     H10F     H10G     H20A     H20B     H20C     H20D
PLAT301_ALERT_3_G Main Residue  Disorder ..............(Resd     1)        4% Note
PLAT343_ALERT_2_G Unusual sp3      Angle Range in Main Residue for         C1 Check
And 6 other PLAT343 Alerts
More ...
PLAT413_ALERT_2_G Short Inter XH3 .. XHn     H9AB     ..H3A       .       2.13 Ang.
                                                   x,y,-1+z  =      1_554 Check
PLAT415_ALERT_2_G Short Inter D-H..H-X       H3A      ..H10G      .       1.91 Ang.
                                                    x,y,1+z  =      1_556 Check
And 2 other PLAT415 Alerts
More ...
PLAT432_ALERT_2_G Short Inter X...Y Contact  N10A     ..C19       .       2.60 Ang.
                                                   x,y,-1+z  =      1_554 Check
PLAT720_ALERT_4_G Number of Unusual/Non-Standard Labels .........           2 Note
             H9AA     H9AB
PLAT860_ALERT_3_G Number of Least-Squares Restraints ............         432 Note
PLAT912_ALERT_4_G Missing # of FCF Reflections Above STh/L=  0.600        222 Note
PLAT969_ALERT_5_G The 'Henn et al.' R-Factor-gap value ..........      12.078 Note
             Predicted wR2: Based on SigI**2  2.35 or SHELX Weight 23.28
PLAT978_ALERT_2_G Number C-C Bonds with Positive Residual Density.          0 Info
PLAT992_ALERT_5_G Repd & Actual _reflns_number_gt Values Differ by          6 Check
```

```
   0 ALERT level A = Most likely a serious problem - resolve or explain
   0 ALERT level B = A potentially serious problem, consider carefully
  49 ALERT level C = Check. Ensure it is not caused by an omission or oversight
  38 ALERT level G = General information/check it is not something unexpected

   3 ALERT type 1 CIF construction/syntax error, inconsistent or missing data
  49 ALERT type 2 Indicator that the structure model may be wrong or deficient
  13 ALERT type 3 Indicator that the structure quality may be low
  19 ALERT type 4 Improvement, methodology, query or suggestion
   3 ALERT type 5 Informative message, check
```

It is advisable to attempt to resolve as many as possible of the alerts in all categories. Often the minor alerts point to easily fixed oversights, errors and omissions in your CIF or refinement strategy, so attention to these fine details can be worthwhile. It is up to the individual to critically assess their own results and, if necessary, seek expert advice.

PLATON version of 26/09/2025; check.def file version of 20/09/2025

Datablock xsw-250807-2-300k-sphere_auto - ellipsoid plot

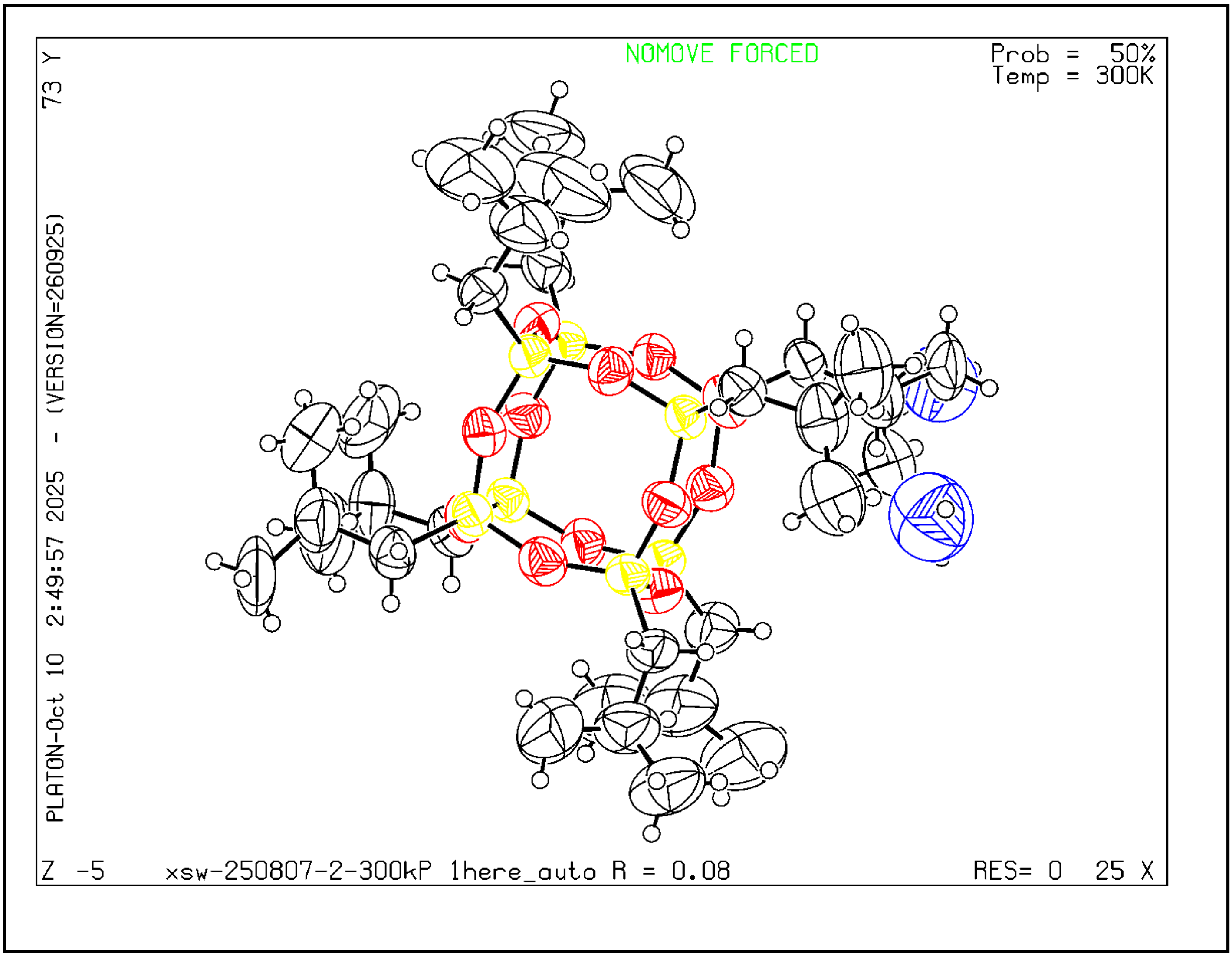

[Download CIF editor (publCIF) from the IUCr](#)
[Download CIF editor (enCIFer) from the CCDC](#)
[Test a new CIF entry](#)
