# Supplementary material for: Organosilicon cluster goes ferroelectric
Source: Natl Sci Rev. 2026 Apr 29;13(14):nwag243. doi: 10.1093/nsr/nwag243 (PMC13411271; doi:10.1093/nsr/nwag243)
Supplement: nwag243_Supplemental_Files [file nwag243_supplemental_files.zip › cif files/checkcif_compound-1_350k.pdf]

No syntax errors found.
Please wait while processing ....

CIF dictionary
Interpreting this report

Datablock: xsw-250807-2-350k

Bond precision:	Si- O = 0.0070 A		Wavelength=1.54184
Cell:	a=16.1357(12)	b=16.1357(12)	c=17.1112(17)
	alpha=90	beta=90	gamma=120
Temperature: 347 K			

	Calculated	Reported
Volume	3858.2(7)	3858.2(7)
Space group	R 3	R 3
Hall group	R 3	R 3
Moiety formula	C31 N O12 Si8	C31 N1 O12 Si8
Sum formula	C31 N O12 Si8	C31 N O12 Si8
Mr	803.03	803.04
Dx,g cm-3	1.037	1.037
Z	3	3
Mu (mm-1)	2.361	2.361
F000	1203.0	1203.0
F000'	1212.50	
h,k,lmax	19,19,20	19,19,20
Nref	3026[1513]	2758
Tmin,Tmax	0.747,0.790	0.552,1.000
Tmin'	0.653	

Correction method= # Reported T Limits: Tmin=0.552 Tmax=1.000
AbsCorr = MULTI-SCAN

Data completeness= 1.82/0.91   Theta(max)= 66.590

R(reflections)= 0.1319(816)   wR2(reflections)= 0.2751(2758)

S = 1.048   Npar= 111

The following ALERTS were generated. Each ALERT has the format
test-name_ALERT_alert-type_alert-level.
Click on the hyperlinks for more details of the test.

Alert level B

PLAT260_ALERT_2_B Large Average Ueq of Residue Including   Si1   0.304 Check

Author Response: It could be due to disorder.

Alert level C

```
STRVA01_ALERT_4_C          Flack test results are ambiguous.
           From the CIF: _refine_ls_abs_structure_Flack     0.430
           From the CIF: _refine_ls_abs_structure_Flack_su     0.170
PLAT042_ALERT_1_C Calc. and Reported MoietyFormula Strings  Differ     Please Check
              Calc: C31 N O12 Si8
              Rep.: C31 N1 O12 Si8
PLAT082_ALERT_2_C High R1 Value ................................         0.13 Report
PLAT084_ALERT_3_C High wR2 Value (i.e. > 0.25) ..................         0.28 Report
PLAT241_ALERT_2_C High   'MainMol' Ueq as Compared to Neighbors of        Si3 Check
PLAT241_ALERT_2_C High   'MainMol' Ueq as Compared to Neighbors of        Si4 Check
PLAT242_ALERT_2_C Low    'MainMol' Ueq as Compared to Neighbors of         O2 Check
PLAT906_ALERT_3_C Large K Value in the Analysis of Variance ......     226.949 Check
And 10 other PLAT906 Alerts
More ...
PLAT911_ALERT_3_C Missing FCF Refl Between Thmin & STh/L=     0.595          11 Report
               0  3  0,    1  1  3,   -1 14  3,   -2 14  5,    8 10  7,  -17 10  9,
             -16  9 11,   -7 10 13,   -9 13 14,   -3 12 15,   -3 11 16,
PLAT918_ALERT_3_C Reflection(s) with I(obs) much Smaller I(calc) .           2 Check
              -5  4  0,   -1  5  0,
PLAT939_ALERT_3_C Large Value of Not (SHELXL) Weight Optimized S .       10.95 Check
```

Alert level G

```
PLAT002_ALERT_2_G Number of Distance or Angle Restraints on AtSite          32 Note
PLAT003_ALERT_2_G Number of Uiso or U(i,j)   Restrained non-H-Atoms         32 Report
PLAT012_ALERT_1_G N.O.K.    _shelx_res_checksum Found in CIF ......     Please Check
PLAT040_ALERT_1_G No H-atoms in this Carbon Containing Compound ..     Please Check
PLAT083_ALERT_2_G SHELXL Second Parameter in WGHT  Unusually Large       10.00 Why ?
```

```
PLAT171_ALERT_4_G The CIF-Embedded .res File Contains EADP Records          3 Report
PLAT172_ALERT_4_G The CIF-Embedded .res File Contains DFIX Records         28 Report
PLAT173_ALERT_4_G The CIF-Embedded .res File Contains DANG Records         10 Report
PLAT176_ALERT_4_G The CIF-Embedded .res File Contains SADI Records          5 Report
PLAT178_ALERT_4_G The CIF-Embedded .res File Contains SIMU Records          1 Report
PLAT186_ALERT_4_G The CIF-Embedded .res File Contains ISOR Records          3 Report
PLAT187_ALERT_4_G The CIF-Embedded .res File Contains RIGU Records          1 Report
PLAT191_ALERT_3_G A Non-default SADI Restraint Value has been used     0.0400 Report
PLAT299_ALERT_4_G Atom Site Occupancy Constrained at ............        0.5 Check
              C1A     C1B     C2A     C2B     C8A     C8B     C9A     C9B
              C10A    C10B    C11A    C11B    C12A    C12B    C13A    C13B
PLAT300_ALERT_4_G Atom Site Occupancy of N1         Constrained at     0.3333 Check
And 7 other PLAT300 Alerts
More ...
PLAT301_ALERT_3_G Main Residue  Disorder .............(Resd    1)        62% Note
PLAT432_ALERT_2_G Short Inter X...Y Contact  C10A     ..C6       .       3.09 Ang.
                                 2/3-x+y,4/3-x,1/3+z  =      6_565 Check
PLAT432_ALERT_2_G Short Inter X...Y Contact  C11B     ..C7       .       3.06 Ang.
                                 2/3-y,1/3+x-y,1/3+z  =      5_555 Check
PLAT773_ALERT_2_G Check long C-C Bond in CIF: C3       --C6              1.84 Ang.
And 5 other PLAT773 Alerts
More ...
PLAT811_ALERT_5_G No ADDSYM Analysis: Too Many Excluded Atoms ....          ! Info
PLAT860_ALERT_3_G Number of Least-Squares Restraints ............         304 Note
PLAT883_ALERT_1_G Absent Datum for _atom_sites_solution_primary ..     Please Do !
PLAT916_ALERT_2_G Hooft y and Flack x Parameter Values Differ by .       0.13 Check
PLAT933_ALERT_2_G Number of HKL-OMIT Records in Embedded .res File          4 Note
                0  3  0,   -3  3  0,    1  1  3,   -1  2 -3,
PLAT969_ALERT_5_G The 'Henn et al.' R-Factor-gap value ..........      4.448 Note
              Predicted wR2: Based on SigI**2  6.18 or SHELX Weight 26.26
```

```
  0 ALERT level A = Most likely a serious problem - resolve or explain
  1 ALERT level B = A potentially serious problem, consider carefully
 21 ALERT level C = Check. Ensure it is not caused by an omission or oversight
 37 ALERT level G = General information/check it is not something unexpected

  4 ALERT type 1 CIF construction/syntax error, inconsistent or missing data
 18 ALERT type 2 Indicator that the structure model may be wrong or deficient
 18 ALERT type 3 Indicator that the structure quality may be low
 17 ALERT type 4 Improvement, methodology, query or suggestion
  2 ALERT type 5 Informative message, check
```

It is advisable to attempt to resolve as many as possible of the alerts in all categories. Often the minor alerts point to easily fixed oversights, errors and omissions in your CIF or refinement strategy, so attention to these fine details can be worthwhile. It is up to the individual to critically assess their own results and, if necessary, seek expert advice.

PLATON version of 26/09/2025; check.def file version of 20/09/2025

Datablock xsw-250807-2-350k - ellipsoid plot

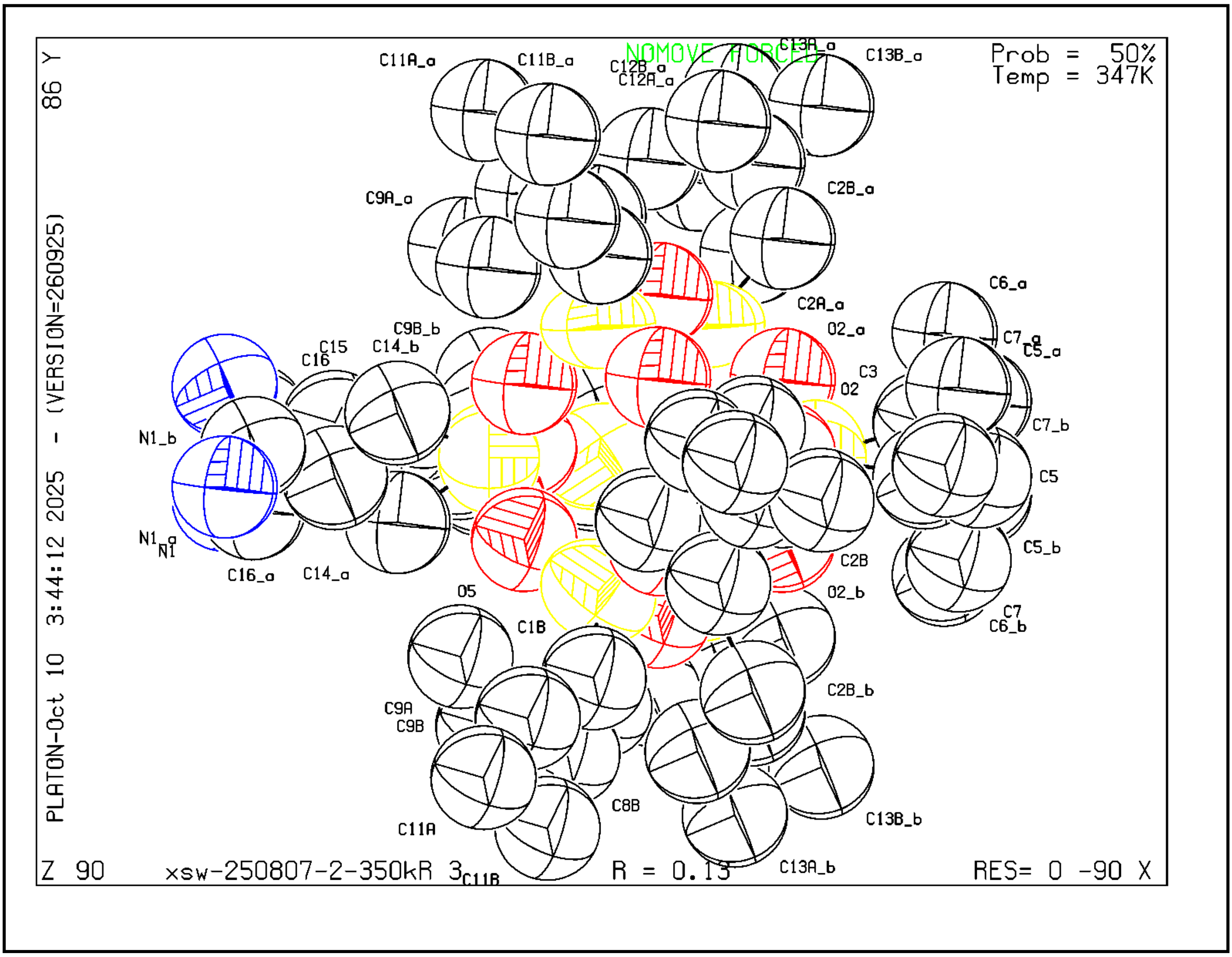

Download CIF editor (publCIF) from the IUCr
Download CIF editor (enCIFer) from the CCDC
Test a new CIF entry
