# Supplementary material for: Organosilicon cluster goes ferroelectric
Source: Natl Sci Rev. 2026 Apr 29;13(14):nwag243. doi: 10.1093/nsr/nwag243 (PMC13411271; doi:10.1093/nsr/nwag243)
Supplement: nwag243_Supplemental_Files [file nwag243_supplemental_files.zip › cif files/checkcif_compound-2-100K.pdf]

No syntax errors found.
Please wait while processing ....

CIF dictionary
Interpreting this report

Datablock: zhy_poss_100k_auto

Bond precision: C-C = 0.0099 A   Wavelength=1.54184

Cell: a=9.9963(2) b=10.8102(2) c=10.9600(1)
alpha=96.770(1) beta=91.073(1) gamma=99.396(1)

Temperature: 100 K

	Calculated	Reported
Volume	1159.45(3)	1159.45(3)
Space group	P 1	P 1
Hall group	P 1	P 1
Moiety formula	C32 H72 O12 Si8	C32 H72 O12 Si8
Sum formula	C32 H72 O12 Si8	C32 H72 O12 Si8
Mr	873.62	873.61
Dx,g cm-3	1.251	1.251
Z	1	1
Mu (mm-1)	2.616	2.616
F000	472.0	472.0
F000'	475.16	
h,k,lmax	12,13,13	12,13,13
Nref	9418[4709]	6339
Tmin,Tmax	0.709,0.731	0.849,1.000
Tmin'	0.643	

Correction method= # Reported T Limits: Tmin=0.849 Tmax=1.000
AbsCorr = MULTI-SCAN

Data completeness= 1.35/0.67   Theta(max)= 73.673

R(reflections)= 0.0379(5915)   wR2(reflections)= 0.1101(6339)

S = 1.081   Npar= 467

The following ALERTS were generated. Each ALERT has the format
test-name_ALERT_alert-type_alert-level.
Click on the hyperlinks for more details of the test.

Alert level B

PLAT915_ALERT_3_B No Flack x Check Done: Low Friedel Pair Coverage 40 %

Alert level C

PLAT213_ALERT_2_C Atom C20 has ADP max/min Ratio ..... 3.1 oblate
PLAT220_ALERT_2_C NonSolvent Resd 1 C Ueq(max)/Ueq(min) Range 4.8 Ratio
PLAT222_ALERT_3_C NonSolvent Resd 1 H Uiso(max)/Uiso(min) Range 6.2 Ratio
PLAT230_ALERT_2_C Hirshfeld Test Diff for Si7 --C29 . 5.7 s.u.
PLAT230_ALERT_2_C Hirshfeld Test Diff for Si8 --C25 . 7.0 s.u.
PLAT340_ALERT_3_C Low Bond Precision on C-C Bonds .... 0.00992 Ang.
PLAT911_ALERT_3_C Missing FCF Refl Between Thmin & STh/L= 0.600 41 Report

```
-9 10  0,   3 -8  6,  10  1  6,   5  4  7,  -5 -6 10,  -6 -5 10,
-3 -4 10,  -4 -3 10,  -3 -7 11,  -4 -6 11,  -3 -6 11,  -5 -5 11,
-4 -5 11,  -3 -5 11,  -5 -4 11,  -4 -4 11,  -3 -4 11,  -6 -3 11,
-5 -3 11,  -4 -3 11,  -3 -3 11,  -6 -2 11,  -5 -2 11,  -4 -2 11,
-6 -1 11,  -1 -6 12,  -3 -5 12,  -2 -5 12,  -4 -4 12,  -3 -4 12,
    (   11 More Missing: see the .ckf listing file)
```

PLAT987_ALERT_1_C The Flack x is >> 0 - Do a BASF/TWIN Refinement Please Check

Alert level G

PLAT033_ALERT_4_G Flack x Value Deviates > 3.0 * Sigma from Zero . 0.210 Note
PLAT111_ALERT_2_G ADDSYM Detects New (Pseudo) Centre of Symmetry . 100 %Fit
PLAT113_ALERT_2_G ADDSYM Suggests Possible Pseudo/New Space-group P-1 Check
Check Model Parameter Symmetry for Reflection Data Support
PLAT154_ALERT_1_G The s.u.'s on the Cell Angles are Equal ..(Note) 0.001 Degree
PLAT171_ALERT_4_G The CIF-Embedded .res File Contains EADP Records 1 Report
PLAT912_ALERT_4_G Missing # of FCF Reflections Above STh/L= 0.600 213 Note
PLAT916_ALERT_2_G Hooft y and Flack x Parameter Values Differ by . 0.23 Check
PLAT941_ALERT_3_G Average HKL Measurement Multiplicity .... 3.0 Low
PLAT969_ALERT_5_G The 'Henn et al.' R-Factor-gap value .... 4.152 Note
Predicted wR2: Based on SigI**2 2.65 or SHELX Weight 10.18
PLAT978_ALERT_2_G Number C-C Bonds with Positive Residual Density. 6 Info

0 ALERT level A = Most likely a serious problem - resolve or explain
1 ALERT level B = A potentially serious problem, consider carefully

```
 8 ALERT level C = Check. Ensure it is not caused by an omission or oversight
10 ALERT level G = General information/check it is not something unexpected

 2 ALERT type 1 CIF construction/syntax error, inconsistent or missing data
 8 ALERT type 2 Indicator that the structure model may be wrong or deficient
 5 ALERT type 3 Indicator that the structure quality may be low
 3 ALERT type 4 Improvement, methodology, query or suggestion
 1 ALERT type 5 Informative message, check
```

It is advisable to attempt to resolve as many as possible of the alerts in all categories. Often the minor alerts point to easily fixed oversights, errors and omissions in your CIF or refinement strategy, so attention to these fine details can be worthwhile. It is up to the individual to critically assess their own results and, if necessary, seek expert advice.

PLATON version of 26/09/2025; check.def file version of 20/09/2025

Datablock zhy_poss_100k_auto - ellipsoid plot

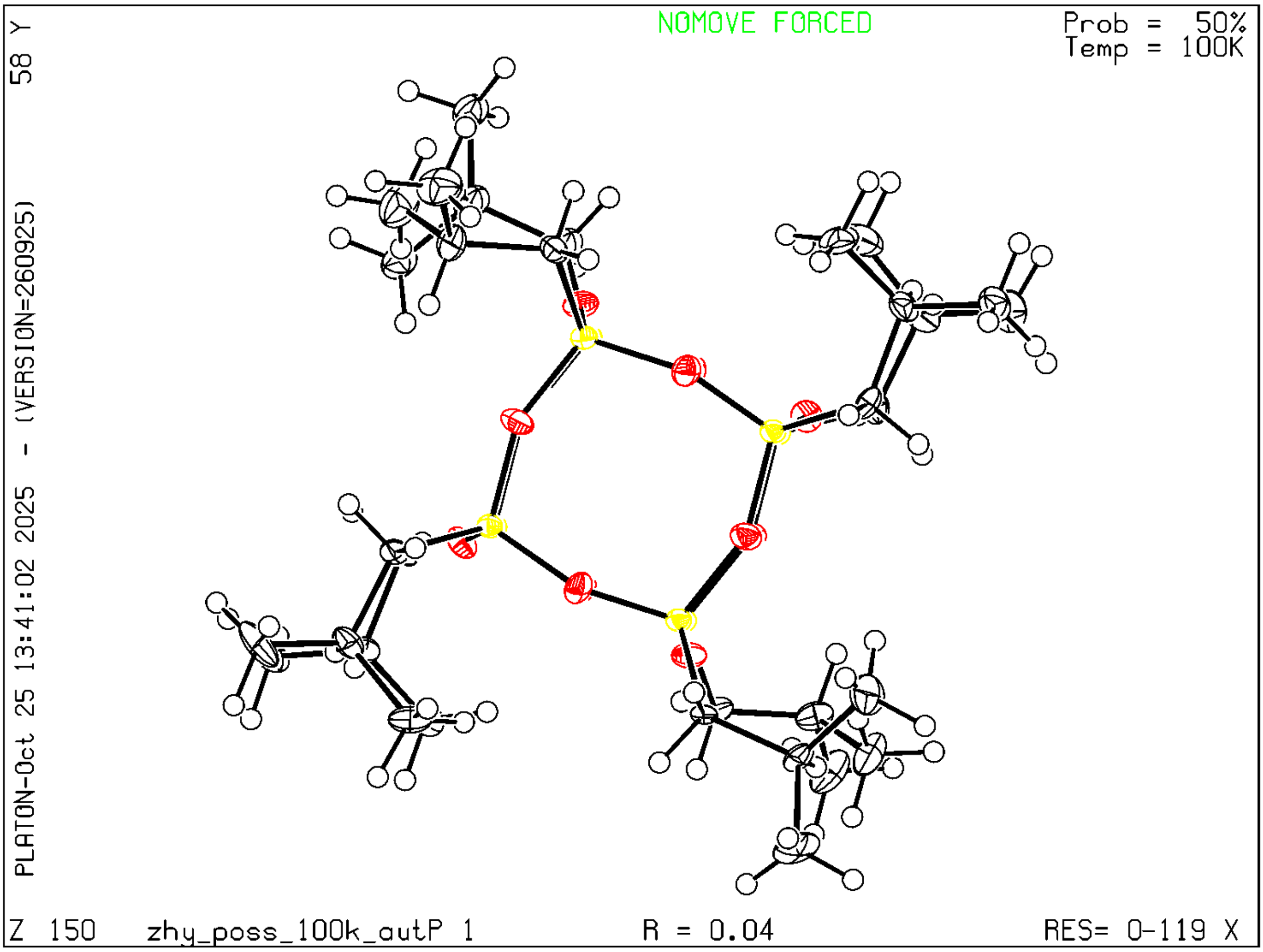

[Download CIF editor (publCIF) from the IUCr](#)
[Download CIF editor (enCIFer) from the CCDC](#)
[Test a new CIF entry](#)
