# Supplementary material for: Organosilicon cluster goes ferroelectric
Source: Natl Sci Rev. 2026 Apr 29;13(14):nwag243. doi: 10.1093/nsr/nwag243 (PMC13411271; doi:10.1093/nsr/nwag243)
Supplement: nwag243_Supplemental_Files [file nwag243_supplemental_files.zip › cif files/checkcif_compound-2-300K.pdf]

No syntax errors found.
Please wait while processing ....

CIF dictionary
Interpreting this report

Datablock: zhy_poss_300k

Bond precision: C-C = 0.0070 A Wavelength=1.54184

Cell: a=10.12467(18) b=11.14837(16) c=11.19548(18)
alpha=93.8033(12) beta=100.4056(14) gamma=90.6432(13)

Temperature: 300 K

	Calculated	Reported
Volume	1239.81(4)	1239.81(3)
Space group	P 1	P 1
Hall group	P 1	P 1
Moiety formula	C32 H72 O12 Si8	C32 H72 O12 Si8
Sum formula	C32 H72 O12 Si8	C32 H72 O12 Si8
Mr	873.62	873.61
Dx,g cm-3	1.170	1.170
Z	1	1
Mu (mm-1)	2.447	2.447
F000	472.0	472.0
F000'	475.16	
h,k,lmax	12,13,13	12,13,13
Nref	10042[5021]	6558
Tmin,Tmax	0.727,0.746	0.930,1.000
Tmin'	0.660	

Correction method= # Reported T Limits: Tmin=0.930 Tmax=1.000
AbsCorr = MULTI-SCAN

Data completeness= 1.31/0.65 Theta(max)= 73.890

R(reflections)= 0.0515(5556) wR2(reflections)= 0.1626(6558)

S = 1.086 Npar= 446

The following ALERTS were generated. Each ALERT has the format
test-name_ALERT_alert-type_alert-level.
Click on the hyperlinks for more details of the test.

Alert level B

PLAT915_ALERT_3_B No Flack x Check Done: Low Friedel Pair Coverage 36 %

Alert level C

STRVA01_ALERT_4_C Flack test results are ambiguous.
From the CIF: _refine_ls_abs_structure_Flack 0.420
From the CIF: _refine_ls_abs_structure_Flack_su 0.050
PLAT230_ALERT_2_C Hirshfeld Test Diff for Si1 --C1 . 6.5 s.u.
PLAT230_ALERT_2_C Hirshfeld Test Diff for Si2 --C5 . 7.0 s.u.
PLAT241_ALERT_2_C High 'MainMol' Ueq as Compared to Neighbors of O4 Check
PLAT241_ALERT_2_C High 'MainMol' Ueq as Compared to Neighbors of O11 Check
PLAT242_ALERT_2_C Low 'MainMol' Ueq as Compared to Neighbors of Si5 Check
And 5 other PLAT242 Alerts
More ...
PLAT340_ALERT_3_C Low Bond Precision on C-C Bonds .... 0.007 Ang.
PLAT911_ALERT_3_C Missing FCF Refl Between Thmin & STh/L= 0.600 43 Report

```
 -7 11  0,   5-12  1,   6-11  1,   5-10  1,   1  1  1,  -7 11  1,
  4-12  2,   5-12  2,   5-11  2,   6-11  2, -12  2  2,   3-12  3,
  4-12  3,   4-11  3,   5-11  3,   6-11  3,   4-10  3,   5-10  3,
  6-10  3,   7-10  3,   3-12  4,   4-12  4,   4-11  4,   5-11  4,
  4-10  4,   5-10  4,   6-10  4,   2-12  5,   3-12  5,   3-11  5,
   (   13 More Missing: see the .ckf listing file)
```

Alert level G

PLAT002_ALERT_2_G Number of Distance or Angle Restraints on AtSite 50 Note
PLAT003_ALERT_2_G Number of Uiso or U(i,j) Restrained non-H-Atoms 43 Report
PLAT012_ALERT_1_G N.O.K. _shelx_res_checksum Found in CIF ...... Please Check
PLAT033_ALERT_4_G Flack x Value Deviates > 3.0 * Sigma from Zero . 0.420 Note
PLAT072_ALERT_2_G SHELXL First Parameter in WGHT Unusually Large 0.10 Report
PLAT171_ALERT_4_G The CIF-Embedded .res File Contains EADP Records 12 Report
PLAT172_ALERT_4_G The CIF-Embedded .res File Contains DFIX Records 14 Report
PLAT178_ALERT_4_G The CIF-Embedded .res File Contains SIMU Records 1 Report
PLAT186_ALERT_4_G The CIF-Embedded .res File Contains ISOR Records 8 Report
PLAT187_ALERT_4_G The CIF-Embedded .res File Contains RIGU Records 1 Report
PLAT188_ALERT_3_G A Non-default SIMU Restraint Value has been used 0.0070 Report

```
PLAT190_ALERT_3_G A Non-default RIGU Restraint Value for First Par     0.0020 Report
PLAT230_ALERT_2_G Hirshfeld Test Diff for    C1          --C2        .        5.7 s.u.
PLAT299_ALERT_4_G Atom Site Occupancy Constrained at ............        0.5 Check
              C10     C11     C12     C14     C15     C16     C18A    C18B
              C19     C20     C27A    C27B    C28A    C28B    C30     C31
              C32     C33     C34     C35     C36     C37     C38     C39
              C40     C41     C42     C43     H9BC    H10     H9BD    H9AA
              H11A    H11B    H11C    H9AB    H12A    H12B    H12C    H14
              H15A    H15B    H15C    H16A    H16B    H16C    H17A    H17B
              H17C    H17D    H18A    H18B    H19A    H19B    H19C    H20A
              H20B    H20C    H26     H26A    H27A    H27B    H27C    H27D
              H27E    H27F    H28D    H28E    H28F    H28G    H28H    H28I
              H29A    H29B    H29C    H29D    H30     H31A    H31B    H31C
              H32A    H32B    H32C    H33     H34A    H34B    H34C    H35A
              H35B    H35C    H36     H37A    H37B    H37C    H38A    H38B
              H38C    H39A    H39B    H39C    H40A    H40B    H40C    H41
              H42A    H42B    H42C    H43A    H43B    H43C
PLAT300_ALERT_4_G Atom Site Occupancy of C2         Constrained at         0.7 Check
And 71 other PLAT300 Alerts
More ...
PLAT301_ALERT_3_G Main Residue  Disorder ..............(Resd     1)         44% Note
PLAT720_ALERT_4_G Number of Unusual/Non-Standard Labels ..........           12 Note
              H1BC    H1BD    H1AA    H1AB    H5BC    H5BD    H5AA    H5AB
              H9BC    H9BD    H9AA    H9AB
PLAT791_ALERT_4_G Model has Chirality at Si2         (Sohncke SpGr)           R Verify
And 3 other PLAT791 Alerts
More ...
PLAT811_ALERT_5_G No ADDSYM Analysis: Too Many Excluded Atoms ....            ! Info
PLAT860_ALERT_3_G Number of Least-Squares Restraints .............          413 Note
PLAT912_ALERT_4_G Missing # of FCF Reflections Above STh/L=  0.600          206 Note
PLAT933_ALERT_2_G Number of HKL-OMIT Records in Embedded .res File            3 Note
               -1 -1 -1,    1  1  1,    3  1  0,
PLAT941_ALERT_3_G Average HKL Measurement Multiplicity ..........          3.3 Low
PLAT969_ALERT_5_G The 'Henn et al.' R-Factor-gap value ...........        6.582 Note
              Predicted wR2: Based on SigI**2  2.47 or SHELX Weight 14.97
PLAT978_ALERT_2_G Number C-C Bonds with Positive Residual Density.            5 Info
```

```
   0 ALERT level A = Most likely a serious problem - resolve or explain
   1 ALERT level B = A potentially serious problem, consider carefully
  13 ALERT level C = Check. Ensure it is not caused by an omission or oversight
  99 ALERT level G = General information/check it is not something unexpected

   1 ALERT type 1 CIF construction/syntax error, inconsistent or missing data
  16 ALERT type 2 Indicator that the structure model may be wrong or deficient
   8 ALERT type 3 Indicator that the structure quality may be low
  86 ALERT type 4 Improvement, methodology, query or suggestion
   2 ALERT type 5 Informative message, check
```

It is advisable to attempt to resolve as many as possible of the alerts in all categories. Often the minor alerts point to easily fixed oversights, errors and omissions in your CIF or refinement strategy, so attention to these fine details can be worthwhile. It is up to the individual to critically assess their own results and, if necessary, seek expert advice.

PLATON version of 26/09/2025; check.def file version of 20/09/2025

Datablock zhy_poss_300k - ellipsoid plot

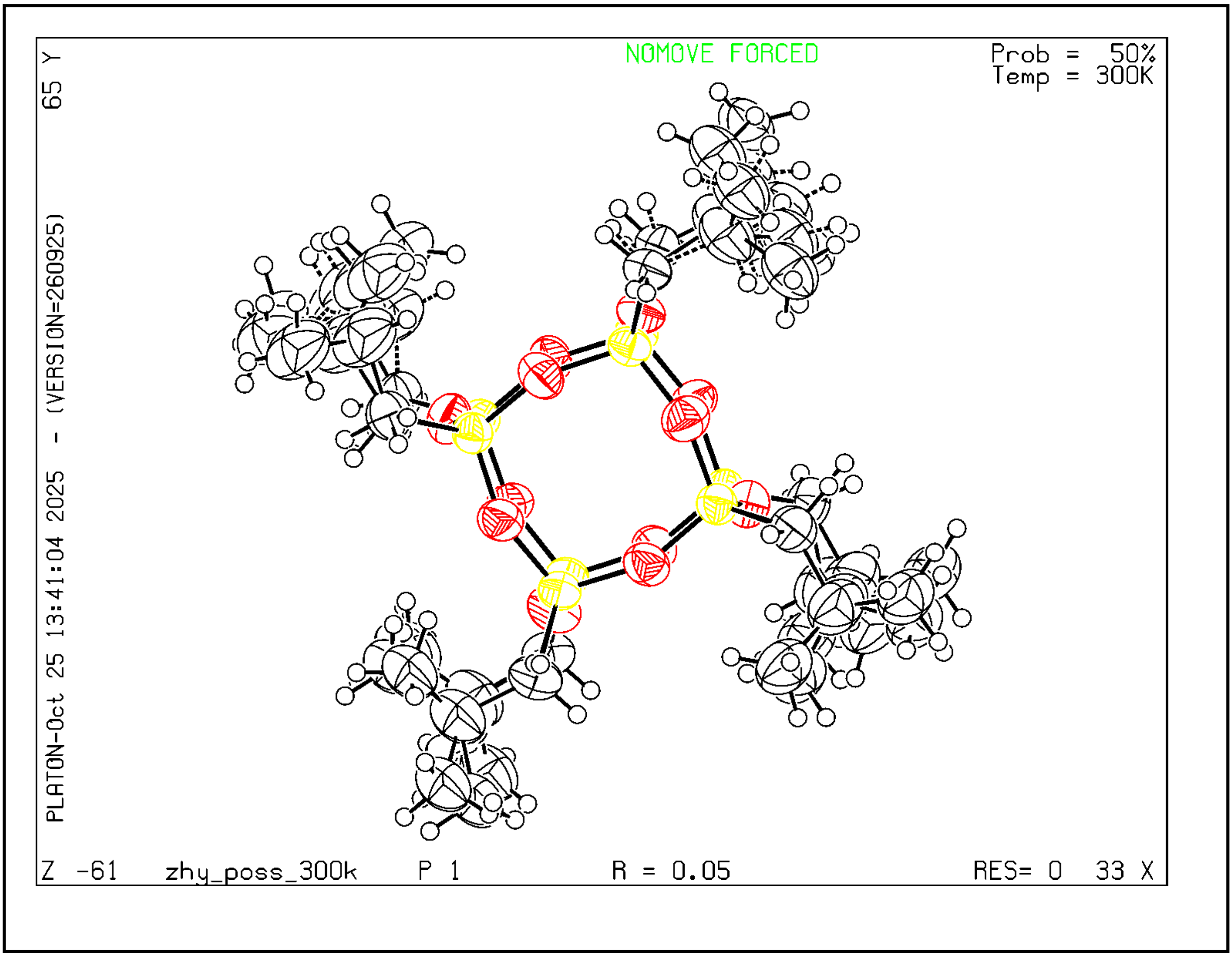

Download CIF editor (publCIF) from the IUCr
Download CIF editor (enCIFer) from the CCDC
Test a new CIF entry
