# Supplementary material for: Organosilicon cluster goes ferroelectric
Source: Natl Sci Rev. 2026 Apr 29;13(14):nwag243. doi: 10.1093/nsr/nwag243 (PMC13411271; doi:10.1093/nsr/nwag243)
Supplement: nwag243_Supplemental_Files [file nwag243_supplemental_files.zip › cif files/checkcif_compound-2-370K.pdf]

No syntax errors found.
Please wait while processing ....

CIF dictionary
Interpreting this report

Datablock: zhy_poss_370k_auto

Bond precision:	Si- O = 0.0248 A		Wavelength=1.54184
Cell:	a=16.2239(11)	b=16.2239(11)	c=17.2393(17)
	alpha=90	beta=90	gamma=120
Temperature: 370 K			

	Calculated	Reported
Volume	3929.7(7)	3929.7(7)
Space group	R 3	R 3
Hall group	R 3	R 3
Moiety formula	C32 O12 Si8	C32 O12 Si8
Sum formula	C32 O12 Si8	C32 O12 Si8
Mr	801.03	801.04
Dx,g cm-3	1.015	1.015
Z	3	3
Mu (mm-1)	2.312	2.312
F000	1200.0	1200.0
F000'	1209.48	
h,k,lmax	19,19,20	19,19,20
Nref	3118[1559]	2880
Tmin,Tmax	0.753,0.794	0.591,1.000
Tmin'	0.658	

Correction method= # Reported T Limits: Tmin=0.591 Tmax=1.000
AbsCorr = MULTI-SCAN

Data completeness= 1.85/0.92   Theta(max)= 66.572

R(reflections)= 0.2117(1231)   wR2(reflections)= 0.2711(2880)

S = 1.064   Npar= 110

The following ALERTS were generated. Each ALERT has the format
test-name_ALERT_alert-type_alert-level.
Click on the hyperlinks for more details of the test.

Alert level A

```
PLAT082_ALERT_2_A High R1 Value ................................        0.21 Report
```

Author Response: The crystal formed twins after a phase transition, which led to poor-quality diffraction spots.

Alert level C

```
PLAT084_ALERT_3_C High wR2 Value (i.e. > 0.25) ...................       0.27 Report
PLAT241_ALERT_2_C High    'MainMol' Ueq as Compared to Neighbors of       Si4 Check
PLAT242_ALERT_2_C Low     'MainMol' Ueq as Compared to Neighbors of        O2 Check
PLAT260_ALERT_2_C Large Average Ueq of Residue Including      Si1       0.263 Check
PLAT723_ALERT_1_C Torsion Calc     -120.00, Rep    -122(91) Dev...       2.00 Sigma
              C1   -C8   -C4   -C8    2_665  1_555   1_555  2_665     #   173 Check
And 11 other PLAT723 Alerts
More ...
PLAT907_ALERT_2_C Flack x > 0.5, Structure Needs to be Inverted? .       0.70 Check
```

Alert level G

```
PLAT002_ALERT_2_G Number of Distance or Angle Restraints on AtSite         32 Note
PLAT003_ALERT_2_G Number of Uiso or U(i,j)   Restrained non-H-Atoms        28 Report
PLAT012_ALERT_1_G No        _shelx_res_checksum Found in CIF ......   Please Check
PLAT032_ALERT_4_G Std. Uncertainty on Flack Parameter Value High .      0.300 Report
PLAT040_ALERT_1_G No H-atoms in this Carbon Containing Compound ..   Please Check
PLAT083_ALERT_2_G SHELXL Second Parameter in WGHT  Unusually Large      20.00 Why ?
PLAT171_ALERT_4_G The CIF-Embedded .res File Contains EADP Records          4 Report
PLAT172_ALERT_4_G The CIF-Embedded .res File Contains DFIX Records         27 Report
PLAT173_ALERT_4_G The CIF-Embedded .res File Contains DANG Records         12 Report
PLAT176_ALERT_4_G The CIF-Embedded .res File Contains SADI Records          4 Report
PLAT186_ALERT_4_G The CIF-Embedded .res File Contains ISOR Records          3 Report
PLAT299_ALERT_4_G Atom Site Occupancy Constrained at ............         0.5 Check
              C1A     C1B     C2A     C2B     C8A     C8B     C9A     C9B
              C10A    C10B    C11A    C11B    C12A    C12B    C13A    C13B
PLAT300_ALERT_4_G Atom Site Occupancy of C1         Constrained at     0.3333 Check
```

```
And 7 other PLAT300 Alerts
More ...
PLAT301_ALERT_3_G Main Residue  Disorder ..............(Resd     1)        62% Note
PLAT432_ALERT_2_G Short Inter X...Y Contact  C11B      ..C6        .       2.98 Ang.
                                        -1/3+x,-2/3+y,1/3+z  =      4_445 Check
PLAT432_ALERT_2_G Short Inter X...Y Contact  C13A      ..C6        .       3.03 Ang.
                                        2/3-x+y,4/3-x,1/3+z  =      6_565 Check
PLAT773_ALERT_2_G Check long C-C Bond in CIF: C3        --C5               1.99 Ang.
And 2 other PLAT773 Alerts
More ...
PLAT811_ALERT_5_G No ADDSYM Analysis: Too Many Excluded Atoms ....           ! Info
PLAT860_ALERT_3_G Number of Least-Squares Restraints .............         245 Note
PLAT933_ALERT_2_G Number of HKL-OMIT Records in Embedded .res File          21 Note
                0  3  0,  -3  3  0,   1  1  3,  -1  2 -3,   0  2 -2,  -3  4 -1,
                1  3  1,  -2  2  2,  -4  4  1,  -1  5  0,  -2  2 -7,  -5  4  0,
                0  2  7,  -1  5 -6,   1  1  6,  -1  2 -6,  -5  4  6,   0  1 -4,
                1  2  5,   0  5  1,  -1  1  1,
```

```
 1 ALERT level A = Most likely a serious problem - resolve or explain
 0 ALERT level B = A potentially serious problem, consider carefully
17 ALERT level C = Check. Ensure it is not caused by an omission or oversight
29 ALERT level G = General information/check it is not something unexpected

14 ALERT type 1 CIF construction/syntax error, inconsistent or missing data
14 ALERT type 2 Indicator that the structure model may be wrong or deficient
 3 ALERT type 3 Indicator that the structure quality may be low
15 ALERT type 4 Improvement, methodology, query or suggestion
 1 ALERT type 5 Informative message, check
```

It is advisable to attempt to resolve as many as possible of the alerts in all categories. Often the minor alerts point to easily fixed oversights, errors and omissions in your CIF or refinement strategy, so attention to these fine details can be worthwhile. It is up to the individual to critically assess their own results and, if necessary, seek expert advice.

PLATON version of 26/09/2025; check.def file version of 20/09/2025

Datablock zhy_poss_370k_auto - ellipsoid plot

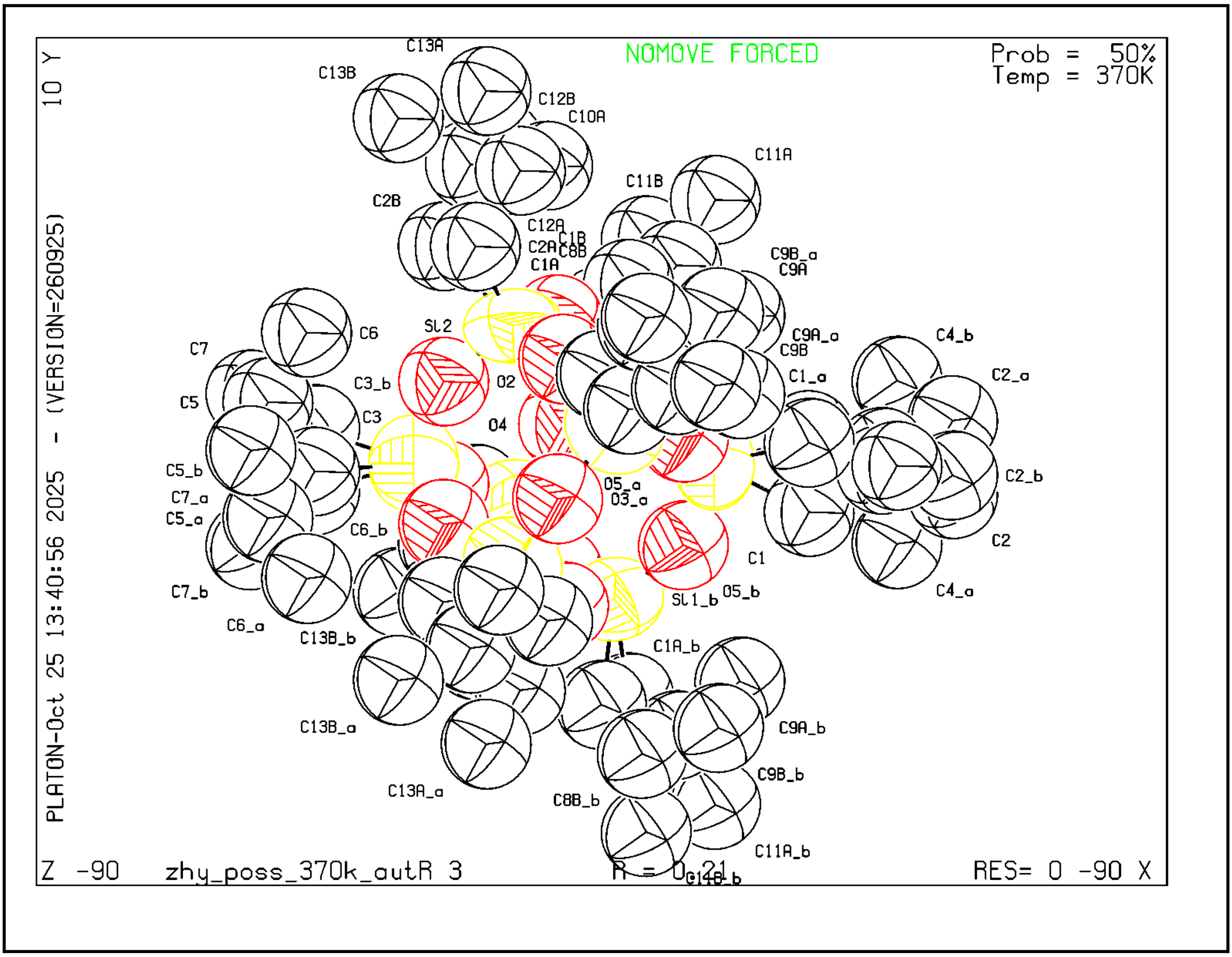

[Download CIF editor (publCIF) from the IUCr](#)
[Download CIF editor (enCIFer) from the CCDC](#)
[Test a new CIF entry](#)
