# Supplementary material for: Organosilicon cluster goes ferroelectric
Source: Natl Sci Rev. 2026 Apr 29;13(14):nwag243. doi: 10.1093/nsr/nwag243 (PMC13411271; doi:10.1093/nsr/nwag243)
Supplement: nwag243_Supplemental_Files [file nwag243_supplemental_files.zip › CIF/checkCIF_NJUZ-Cd.pdf]

checkCIF (basic structural check) running

Checking for embedded fcf data in CIF ...
Found embedded fcf data in CIF. Extracting fcf data from uploaded CIF, please wait **. . .**

checkCIF/PLATON (basic structural check)

Structure factors have been supplied for datablock(s) cu_241111lu_zjl_0m

THIS REPORT IS FOR GUIDANCE ONLY. IF USED AS PART OF A REVIEW PROCEDURE FOR PUBLICATION, IT SHOULD NOT REPLACE THE EXPERTISE OF AN EXPERIENCED CRYSTALLOGRAPHIC REFEREE.

No syntax errors found. CIF dictionary
Please wait while processing .... Interpreting this report

Structure factor report

Datablock: cu_241111lu_zjl_0m

```
Bond precision:          C-C = 0.0171 A                     Wavelength=1.54178
Cell:        a=12.5533(8)        b=16.268(2)        c=19.7709(11)
             alpha=68.727(6)     beta=89.130(4)     gamma=81.363(7)
Temperature: 223 K
                   Calculated                         Reported
Volume             3716.4(6)                          3716.4(6)
Space group        P -1                               P -1
Hall group         -P 1                               -P 1
Moiety formula     C54 H28 Cd N9 S4, C6 H2 N2 [+ solvent]   C54 H28 Cd N9 S4, C6 H2 N2
Sum formula        C60 H30 Cd N11 S4 [+ solvent]      C60 H30 Cd N11 S4
Mr                 1145.60                            1145.59
Dx,g cm-3          1.024                              1.024
Z                  2                                  2
Mu (mm-1)          3.697                              3.697
F000               1158.0                             1158.0
F000'              1163.64
h,k,lmax           14,19,23                           14,19,23
Nref               13128                              13044
Tmin,Tmax          0.649,0.691                        0.173,0.322
Tmin'              0.589
Correction method= # Reported T Limits: Tmin=0.173 Tmax=0.322 AbsCorr = NONE
Data completeness= 0.994               Theta(max)= 66.595
R(reflections)= 0.0996( 5922)                    wR2(reflections)= 0.2711( 13044)
S = 1.076                   Npar= 705
```

The following ALERTS were generated. Each ALERT has the format
test-name_ALERT_alert-type_alert-level.
Click on the hyperlinks for more details of the test.

Alert level C

PLAT026_ALERT_3_C Ratio Observed / Unique Reflections (too) Low .. 45% Check
PLAT084_ALERT_3_C High wR2 Value (i.e. > 0.25) .... 0.27 Report
PLAT213_ALERT_2_C Atom Cd1 has ADP max/min Ratio ..... 3.1 prolat
PLAT220_ALERT_2_C NonSolvent Resd 1 N Ueq(max)/Ueq(min) Range 3.7 Ratio
PLAT241_ALERT_2_C High 'MainMol' Ueq as Compared to Neighbors of Cd1 Check
And 10 other PLAT241 Alerts
More ...
PLAT242_ALERT_2_C Low 'MainMol' Ueq as Compared to Neighbors of N1 Check
And 15 other PLAT242 Alerts
More ...
PLAT250_ALERT_2_C Large U3/U1 Ratio for <U(i,j)> Tensor(Resd 1) 3.9 Note
PLAT250_ALERT_2_C Large U3/U1 Ratio for <U(i,j)> Tensor(Resd 2) 2.2 Note
PLAT260_ALERT_2_C Large Average Ueq of Residue Including Cd1 0.138 Check
PLAT260_ALERT_2_C Large Average Ueq of Residue Including N9 0.210 Check

PLAT342_ALERT_3_C Low Bond Precision on C-C Bonds .... 0.01715 Ang.
PLAT905_ALERT_3_C Negative K value in the Analysis of Variance ... -11.542 Report
PLAT906_ALERT_3_C Large K Value in the Analysis of Variance ...... 2.110 Check
PLAT911_ALERT_3_C Missing FCF Refl Between Thmin & STh/L= 0.595 83 Report

```
 4  0  0,   1  1  0,   1  2  0,   3  2  0,   5  2  0,   0  6  0,
-2 -6  1,  -1 -2  1,  -2 -1  1,   0 -1  1,  -3  0  1,  -7  1  1,
-4  1  1,  -2  1  1,   0  1  1,   1  1  1,  -7  2  1,   0  2  1,
 7  4  1,   6  6  1,   3  9  1,   7-12  2,  -1 -1  2,   0 -1  2,
-1  0  2,  -4  1  2,  -1  2  2,  -1 -1  3,  -1  0  3,   0  0  3,
   (  53 More Missing: see the .ckf listing file)
```

Alert level G

PLAT002_ALERT_2_G Number of Distance or Angle Restraints on AtSite 12 Note
PLAT003_ALERT_2_G Number of Uiso or U(i,j) Restrained non-H-Atoms 21 Report
PLAT004_ALERT_5_G Polymeric Structure Found with Maximum Dimension 3 Info
PLAT072_ALERT_2_G SHELXL First Parameter in WGHT Unusually Large 0.10 Report
PLAT172_ALERT_4_G The CIF-Embedded .res File Contains DFIX Records 4 Report
PLAT176_ALERT_4_G The CIF-Embedded .res File Contains SADI Records 4 Report
PLAT178_ALERT_4_G The CIF-Embedded .res File Contains SIMU Records 4 Report
PLAT186_ALERT_4_G The CIF-Embedded .res File Contains ISOR Records 2 Report
PLAT188_ALERT_3_G A Non-default SIMU Restraint Value has been used 0.0200 Report

And 3 other PLAT188 Alerts

More ...

PLAT191_ALERT_3_G A Non-default SADI Restraint Value has been used 0.0400 Report
PLAT302_ALERT_4_G Anion/Solvent/Minor-Residue Disorder (Resd 2) 25% Note
PLAT343_ALERT_2_G Unusual sp? Angle Range in Main Residue for C43 Check
PLAT367_ALERT_2_G Long? C(sp?)-C(sp?) Bond C43 - C44 . 1.60 Ang.
PLAT432_ALERT_2_G Short Inter X...Y Contact C4 ..N9A . 2.48 Ang.
x,y,z = 1_555 Check

And 8 other PLAT432 Alerts

More ...

PLAT606_ALERT_4_G Solvent Accessible VOID(S) in Crystal Structure ! Info
PLAT860_ALERT_3_G Number of Least-Squares Restraints .... 146 Note
PLAT868_ALERT_4_G ALERTS Due to the Use of _smtbx_masks Suppressed ! Info
PLAT870_ALERT_4_G ALERTS Related to Twinning Effects Suppressed .. ! Info
PLAT910_ALERT_3_G Missing FCF Reflection(s) Below Theta(Min)[Deg]= 2.95 Note
0 0 1,
PLAT913_ALERT_3_G Missing # of Very Strong Reflections in FCF .... 1 Note
1 1 1,
PLAT931_ALERT_5_G CIFcalcFCF Twin Law (0 1 0) Est.d BASF 0.19 Check
PLAT933_ALERT_2_G Number of HKL-OMIT Records in Embedded .res File 10 Note

```
-4  1  1,  -4  1  2,  -1 -1  3,  -1  0  6,   0 -1  1,   0 -1  2,
 0  0  3,   3  4  8,   5  1  5,   9  5  9,
```

PLAT941_ALERT_3_G Average HKL Measurement Multiplicity .... 1.0 Low
PLAT969_ALERT_5_G The 'Henn et al.' R-Factor-gap value .... 2.950 Note
Predicted wR2: Based on SigI**2 9.19 or SHELX Weight 25.19

0 **ALERT level A** = Most likely a serious problem - resolve or explain
0 **ALERT level B** = A potentially serious problem, consider carefully
39 **ALERT level C** = Check. Ensure it is not caused by an omission or oversight
35 **ALERT level G** = General information/check it is not something unexpected

0 ALERT type 1 CIF construction/syntax error, inconsistent or missing data
48 ALERT type 2 Indicator that the structure model may be wrong or deficient
15 ALERT type 3 Indicator that the structure quality may be low
8 ALERT type 4 Improvement, methodology, query or suggestion
3 ALERT type 5 Informative message, check

It is advisable to attempt to resolve as many as possible of the alerts in all categories. Often the minor alerts point to easily fixed oversights, errors and omissions in your CIF or refinement strategy, so attention to these fine details can be worthwhile. It is up to the individual to critically assess their own results and, if necessary, seek expert advice.

PLATON version of 26/09/2025; check.def file version of 20/09/2025

duplicate check

No duplication found

Datablock cu_241111lu_zjl_0m - ellipsoid plot

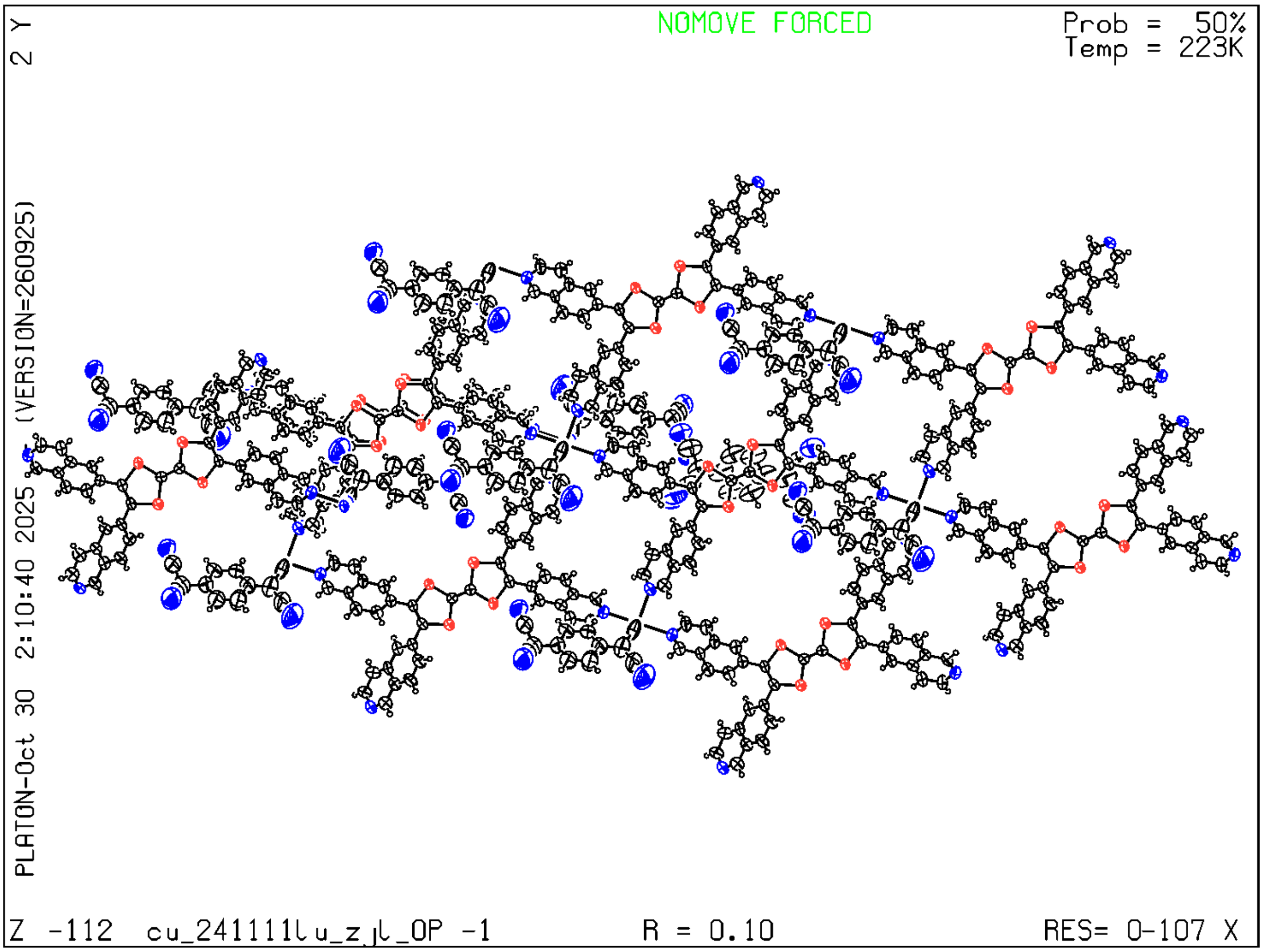

Download CIF editor (publCIF) from the IUCr
Download CIF editor (enCIFer) from the CCDC
Test a new CIF entry
