# Supplementary material for: Organosilicon cluster goes ferroelectric
Source: Natl Sci Rev. 2026 Apr 29;13(14):nwag243. doi: 10.1093/nsr/nwag243 (PMC13411271; doi:10.1093/nsr/nwag243)
Supplement: nwag243_Supplemental_Files [file nwag243_supplemental_files.zip › CIF/checkCIF_NJUZ-Int.pdf]

checkCIF (basic structural check) running

Checking for embedded fcf data in CIF ...
Found embedded fcf data in CIF. Extracting fcf data from uploaded CIF, please wait . . .

checkCIF/PLATON (basic structural check)

Structure factors have been supplied for datablock(s) cu_250106lu_zjl_zn_cu_0m

THIS REPORT IS FOR GUIDANCE ONLY. IF USED AS PART OF A REVIEW PROCEDURE FOR PUBLICATION, IT SHOULD NOT REPLACE THE EXPERTISE OF AN EXPERIENCED CRYSTALLOGRAPHIC REFEREE.

No syntax errors found. CIF dictionary
Please wait while processing .... Interpreting this report

Structure factor report

Datablock: cu_250106lu_zjl_zn_cu_0m

```
Bond precision:          C-C = 0.0124 A                    Wavelength=1.54178
Cell:        a=12.2699(6)          b=16.4830(14)        c=19.5674(9)
             alpha=69.209(5)       beta=88.991(4)       gamma=81.621(5)
Temperature: 193 K
                     Calculated                        Reported
Volume               3657.7(4)                         3657.7(4)
Space group          P -1                              P-1
Hall group           -P 1                              -P 1
Moiety formula       C54 H28 N8 S4 Zn, C6 H2 N2 [+ solvent]   C54 H28 N8 S4 Zn, C6 H2 N2
Sum formula          C60 H30 N10 S4 Zn [+ solvent]     C60 H30 N10 S4 Zn
Mr                   1084.57                           1084.55
Dx,g cm-3            0.985                             0.985
Z                    2                                 2
Mu (mm-1)            1.822                             1.822
F000                 1108.0                            1108.0
F000'                1110.15
h,k,lmax             14,19,23                          14,19,23
Nref                 13385                             13336
Tmin,Tmax            0.789,0.849                       0.213,0.324
Tmin'                0.789
Correction method= # Reported T Limits: Tmin=0.213 Tmax=0.324 AbsCorr = NONE
Data completeness= 0.996              Theta(max)= 68.243
R(reflections)= 0.0970( 5031)                   wR2(reflections)= 0.2896( 13336)
S = 0.854                  Npar= 677
```

The following ALERTS were generated. Each ALERT has the format
test-name_ALERT_alert-type_alert-level.
Click on the hyperlinks for more details of the test.

Alert level B

PLAT026_ALERT_3_B Ratio Observed / Unique Reflections (too) Low .. 38% Check

Author Response: Due to weak diffraction of the sample.

Alert level C

PLAT084_ALERT_3_C High wR2 Value (i.e. > 0.25) .... 0.29 Report
PLAT241_ALERT_2_C High 'MainMol' Ueq as Compared to Neighbors of C50 Check
PLAT250_ALERT_2_C Large U3/U1 Ratio for <U(i,j)> Tensor(Resd 1) 2.9 Note
PLAT260_ALERT_2_C Large Average Ueq of Residue Including N9 0.103 Check
PLAT334_ALERT_2_C Small <C-C> Benzene Dist. C57 -C59_h . 1.36 Ang.
PLAT341_ALERT_3_C Low Bond Precision on C-C Bonds .... 0.01244 Ang.
PLAT361_ALERT_2_C Long C(sp3)-C(sp3) Bond C51 - C51_g . 1.70 Ang.
PLAT905_ALERT_3_C Negative K value in the Analysis of Variance ... -3.527 Report
PLAT911_ALERT_3_C Missing FCF Refl Between Thmin & STh/L= 0.600 49 Report

```
 1  0  0,   5  5  0,  -2 -2  1,  -2 -1  1,  -2  1  1,   4  5  1,
-1 -1  2,   0  0  2,   1  0  2,  -2  1  2,  -3  2  2,  -1  2  2,
-2  3  2,  -4 -6  3,  -4 -5  3,  -3 -4  3,  -3  1  3,   6  2  3,
-5 -5  4,   2 -4  4,   4  0  4,  -4  1  4,   0  2  4,   2  3  4,
12 13  4,  -3 -6  5,  -5 -5  5,   5  1  5,  13  3  7, -12 -2  8,
    (   19 More Missing: see the .ckf listing file)
```

Alert level G

PLAT002_ALERT_2_G Number of Distance or Angle Restraints on AtSite 2 Note
PLAT004_ALERT_5_G Polymeric Structure Found with Maximum Dimension 2 Info
PLAT072_ALERT_2_G SHELXL First Parameter in WGHT Unusually Large 0.15 Report
PLAT172_ALERT_4_G The CIF-Embedded .res File Contains DFIX Records 1 Report
PLAT606_ALERT_4_G Solvent Accessible VOID(S) in Crystal Structure ! Info
PLAT794_ALERT_5_G Tentative Bond Valency for Zn1 (II) . 1.48 Info
PLAT860_ALERT_3_G Number of Least-Squares Restraints .... 1 Note
PLAT868_ALERT_4_G ALERTS Due to the Use of _smtbx_masks Suppressed ! Info
PLAT870_ALERT_4_G ALERTS Related to Twinning Effects Suppressed .. ! Info
PLAT908_ALERT_2_G Max. Perc. Data with I > 2*s(I) per Res.Shell . 69.54% Note
PLAT931_ALERT_5_G CIFcalcFCF Twin Law (0 1 0) Est.d BASF 0.25 Check
PLAT933_ALERT_2_G Number of HKL-OMIT Records in Embedded .res File 7 Note

```
-2 -1  1,  -2  1  2,  -2  3  2,  -1 -1  2,   0  0  2,   0  2  4,
 1  0  0,
```

PLAT941_ALERT_3_G Average HKL Measurement Multiplicity .... 1.0 Low
PLAT969_ALERT_5_G The 'Henn et al.' R-Factor-gap value .... 2.065 Note
Predicted wR2: Based on SigI**2 14.02 or SHELX Weight 33.92

---

0 **ALERT level A** = Most likely a serious problem - resolve or explain
1 **ALERT level B** = A potentially serious problem, consider carefully
9 **ALERT level C** = Check. Ensure it is not caused by an omission or oversight
14 **ALERT level G** = General information/check it is not something unexpected

0 ALERT type 1 CIF construction/syntax error, inconsistent or missing data
9 ALERT type 2 Indicator that the structure model may be wrong or deficient
7 ALERT type 3 Indicator that the structure quality may be low
4 ALERT type 4 Improvement, methodology, query or suggestion
4 ALERT type 5 Informative message, check

It is advisable to attempt to resolve as many as possible of the alerts in all categories. Often the minor alerts point to easily fixed oversights, errors and omissions in your CIF or refinement strategy, so attention to these fine details can be worthwhile. It is up to the individual to critically assess their own results and, if necessary, seek expert advice.

PLATON version of 26/09/2025; check.def file version of 20/09/2025

duplicate check

No duplication found

Datablock cu_250106lu_zjl_zn_cu_0m - ellipsoid plot

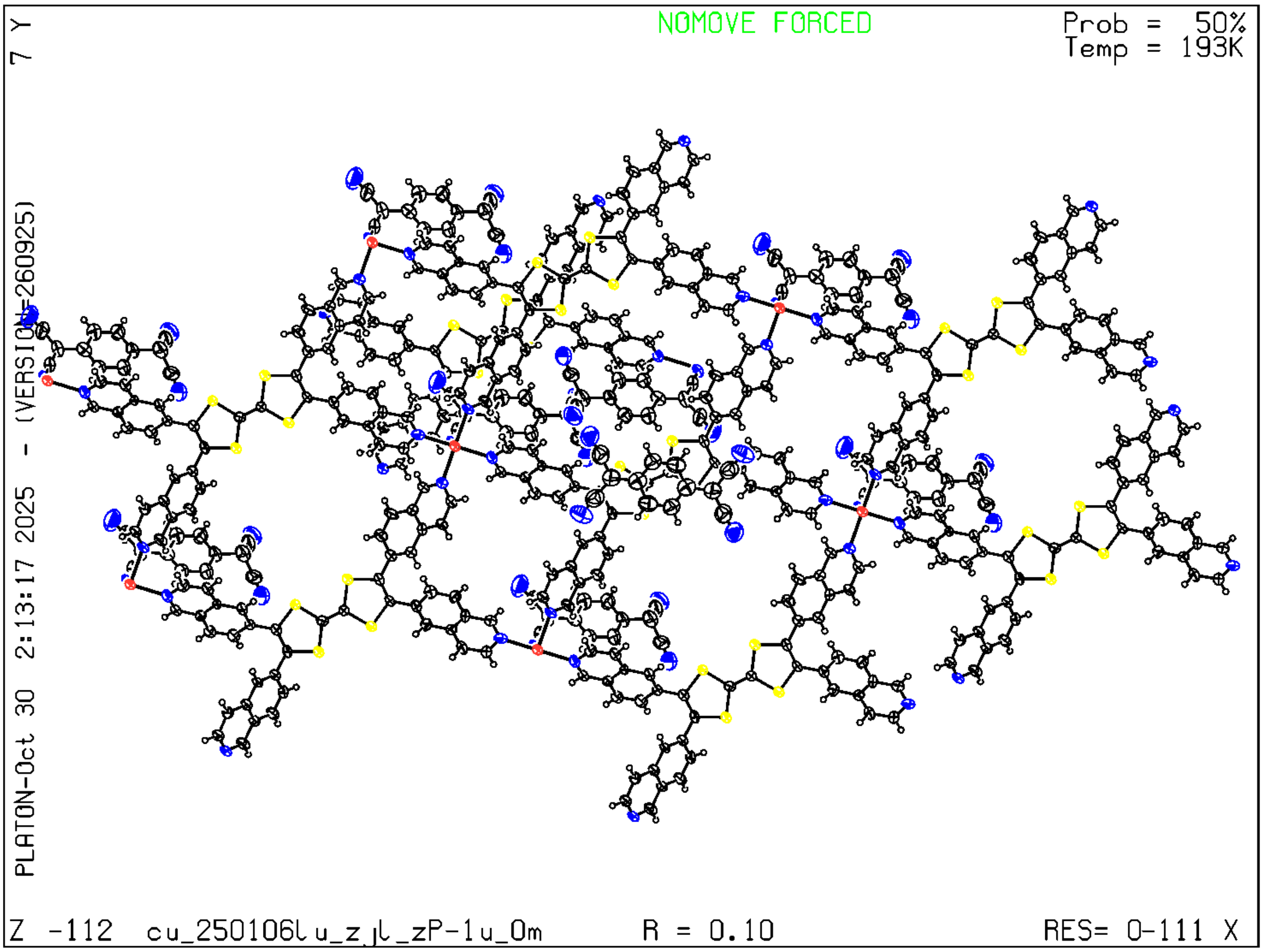

Download CIF editor (publCIF) from the IUCr
Download CIF editor (enCIFer) from the CCDC
Test a new CIF entry
