# Supplementary material for: Organosilicon cluster goes ferroelectric
Source: Natl Sci Rev. 2026 Apr 29;13(14):nwag243. doi: 10.1093/nsr/nwag243 (PMC13411271; doi:10.1093/nsr/nwag243)
Supplement: nwag243_Supplemental_Files [file nwag243_supplemental_files.zip › Revised_SI_without_marked_changes-2517.pdf]

Supplementary Information for

Reversible N_2 Binding and Photoreduction at Dinuclear Sites in Dynamic Metal-Organic Frameworks

Anqi Zhang[1], Zedong Zhang[1], Danyang Xu[1,2], Keke Tu[3], Xiaocheng Zhou[1], Zixuan Chen[3], Yan Xiong[1,2], Shuai Yuan[1], Jing-Lin Zuo[1,*]

[1] State Key Laboratory of Coordination Chemistry, School of Chemistry and Chemical Engineering, Nanjing University, Nanjing 210023, China.

[2] Suzhou Key Laboratory of Green Intelligent Manufacturing of New Energy Materials and Devices, Institute of Green Chemistry and Engineering, Nanjing University, Suzhou 215163, China.

[3] State Key Laboratory of Analytical Chemistry for Life Science, School of Chemistry and Chemical Engineering, Nanjing University, Nanjing 210023, China.

[†]These authors contributed equally to this work.

*Correspondence: zuojl@nju.edu.cn.

Characterization

The C, H, and N microanalyses were carried out using a Vario Micro Cube elemental analyzer. For Fourier-transform infrared (FTIR) spectroscopy measurements, the samples were prepared into KBr pellets, and the FTIR spectra were recorded in the range of 4,000-400 cm^{-1} on a Vector22 Bruker spectrophotometer. PXRD patterns were obtained at room temperature on a Bruker D8 ADVANCE instrument (Cu $K\alpha_1$, λ = 1.54056 Å; $K\alpha_2$, λ = 1.54439 Å) at a rate of 0.1° s^{-1}. The room-temperature EPR spectra were obtained using a Bruker ER-420 spectrometer with a 9.4452 GHz modulating magnetic field in the X band. UV-Vis extinction spectra were recorded on a UV-3600 spectrophotometer (Shimadzu, Kyoto, Japan).

SERS spectra in colloid state were obtained with a glass slide for liquid samples using a Renishaw in Via–Reflex Raman microscope system (Renishaw, U.K.). A Helium-neon laser at 633 nm was used for excitation, and spectra were acquired using a 50× working objective lens on a sample at laser power of 17 mW and exposure time of 10 s.

Single crystal data were collected on a Bruker D8 Venture Photon II instrument (Cu Kα radiation, λ = 1.54178 Å) at 223(2) K and the data were reduced using APEX3 software. Absorption corrections were applied using the SADABS method. The crystal structures were solved and refined against F^2 by the full–matrix least-squares method using the SHELXL-2016/6 program. The positions of the metal atoms and their first coordination spheres were located from direct-method electron-density maps. All non-hydrogen atoms were refined with anisotropic thermal parameters and hydrogen atoms were calculated theoretically, placed onto specific atoms, and refined isotropically with fixed thermal factors.

Synthesis of the L ligand

Tetra(isoquinolin-6-yl)tetrathiafulvalene (L) was prepared by a reflux reaction process, as illustrated in Supplementary Fig. 1. Briefly, $Pd(OAc)_2$ (84 mg), P(*t*-Bu_3).HBF_4 (320 mg) and Cs_2CO_3 (240 mg) were placed in a reaction flask containing 1,4-dioxane (50 mL) under a N_2 atmosphere. A mixture of TTF (300 mg) and 6-bromoisoquinoline (1.8 g) was added. The reaction mixture was refluxed for 48 h at 115 °C. The organic compounds were extracted three times with chloroform. The organic compounds were purified by column chromatography with silica gel using petroleum ether-dichloromethane as the fluid phase to afford target product L as a dark green solid powder (yield: 73%).

Synthesis of Li(TCNQ)

Briefly, tetracyanoquinodimethane (TCNQ, 6.7 g) and LiI (13.4 g) were placed in dry boiling acetonitrile under a N_2 atmosphere. The mixture was stirred for 1 h and cooled to room temperature. The light-precipitated purple solid (5.9 g) was washed and filtered several times with acetonitrile and diethyl ether. Then, the product was dried under reduced pressure

overnight (yield: 65 % based on TCNQ).

Measurement of photocatalytic NRR activity

The concentration of yield ammonia was spectrophotometrically measured by the indophenol blue indicator method. Briefly, 2 mL of the product solution, 2 mL of a 1 M NaOH solution with 5 % salicylic acid and 5 % sodium citrate, 1 mL of 0.05 M NaClO, and 0.2 mL of 1 % sodium nitroferricyanide (III) dihydrate ($C_5FeN_6Na_2O{\cdot}2\,H_2O$) solution were uniformly mixed. After standing in the dark for 2 hours, the concentration of indophenol blue formed was detected using a ultraviolet-visible (UV-Vis) spectrophotometer at a wavelength of 655 nm in the absorption spectrum.

Purification of $^{15}N_2$ isotope gas

Before isotope-labelling experiments, the $^{15}N_2$ feed gas (98 atom%, Sigma) was purified by passing it through a Cu impurity trap before entering the reactor (**Supplementary Fig. 30**). The trap consisted of a U-shaped stainless-steel tube packed with Cu–Zn–Al oxide catalyst, which was pre-reduced in 5% H_2/Ar at 300 °C for 2 h, purged with Ar at 300 °C for 30 min, and then cooled to −100 °C in an ethanol/liquid nitrogen slurry. This purification step was introduced to suppress possible interference from trace ammonia and NO_x impurities in the commercial $^{15}N_2$ gas during isotope-labelling measurements.

Raman calculation method

The Raman position of the $^{15}N{\equiv}^{15}N$ stretching vibration is verified by Hooke's law (reduced mass model):

$$\nu = \frac{1}{2\pi c}\sqrt{\frac{k}{\mu}}$$

and

$$\mu = \frac{M_A\,M_B}{M_A + M_B}$$

where *c* is the velocity of light, *K* is the force constant of the bond between A and B, and μ is the reduced mass of atoms A and B (with masses M_A and M_B) on the basis of the $^{14}N{\equiv}^{14}N$ stretching vibration with a calculated position at approximately 1,920 cm^{-1}.

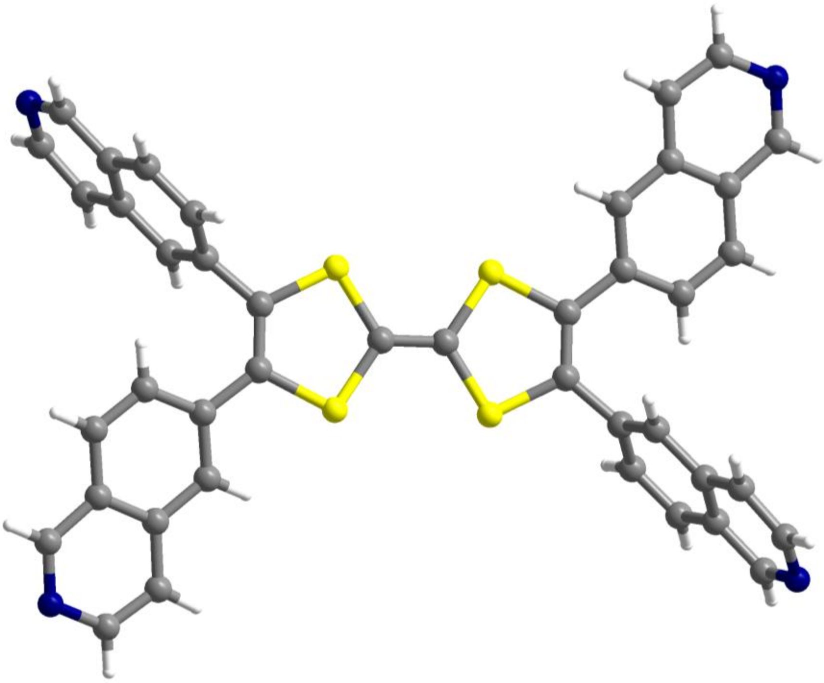

Supplementary Fig. 1. crystal structure of L.

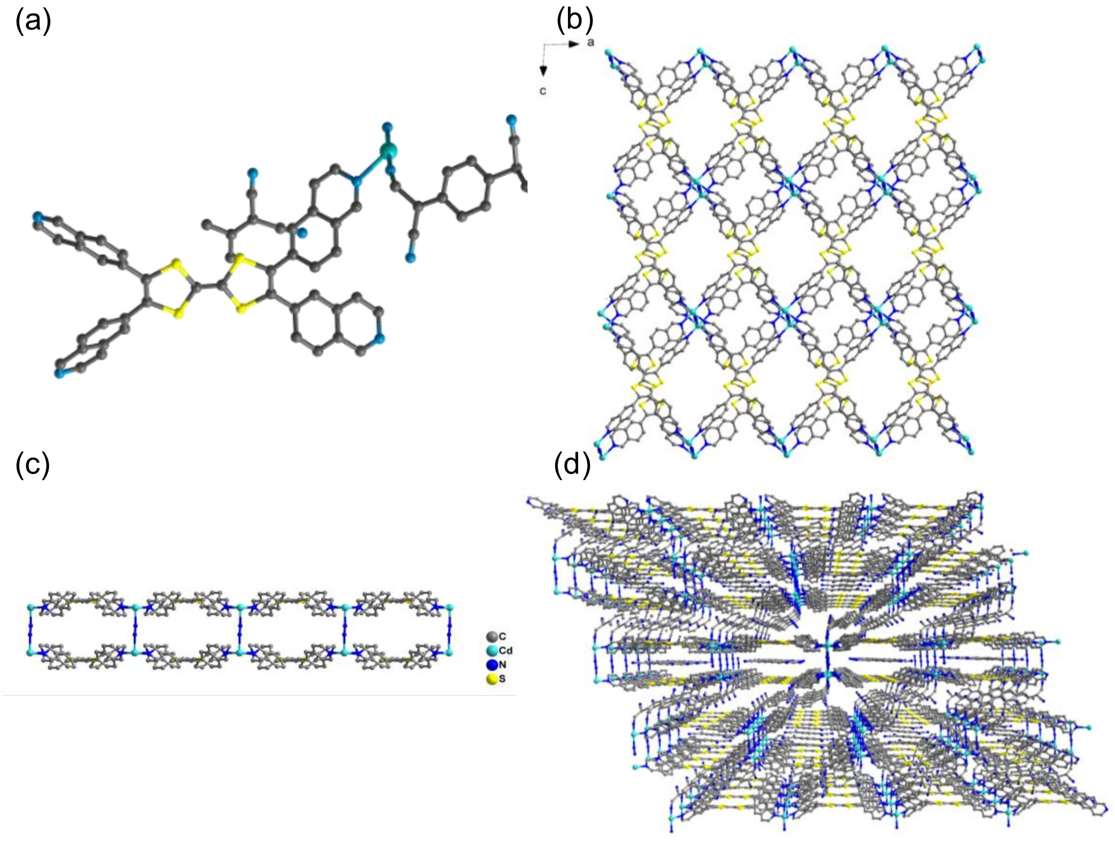

Supplementary Fig. 2. Schematic structure diagram of NJUZ-Cd. (a) The 2D layered structure of NJUZ-Cd is viewed along the b, c axis. (b) The 3D layered structure of NJUZ-Cd.

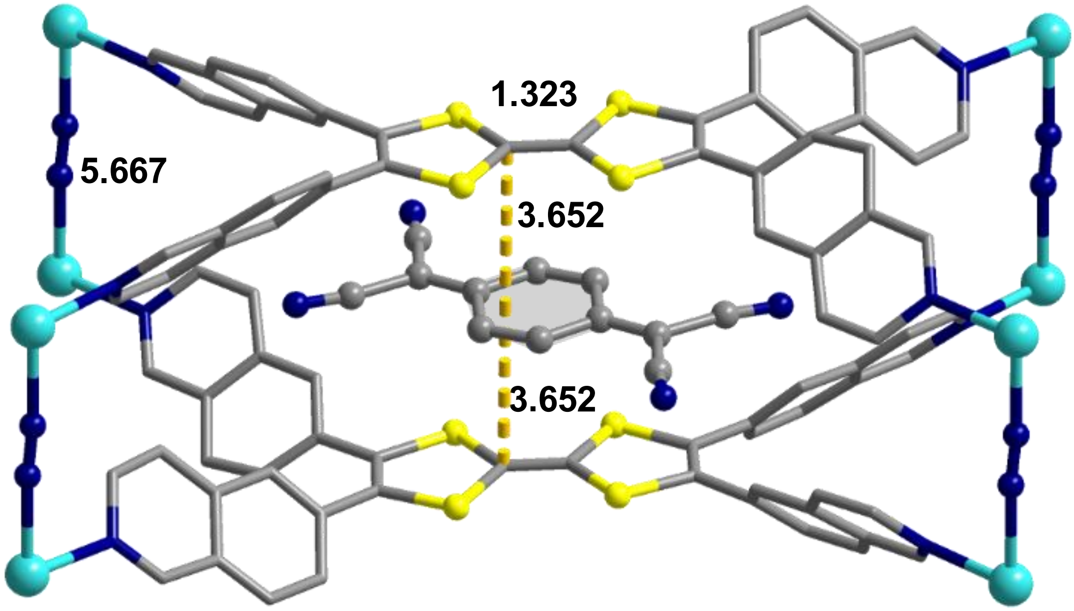

Supplementary Fig. 3. The interactions of π···π stacking in the cage of $[(L)_2(Cd_2N_2)_4]$.

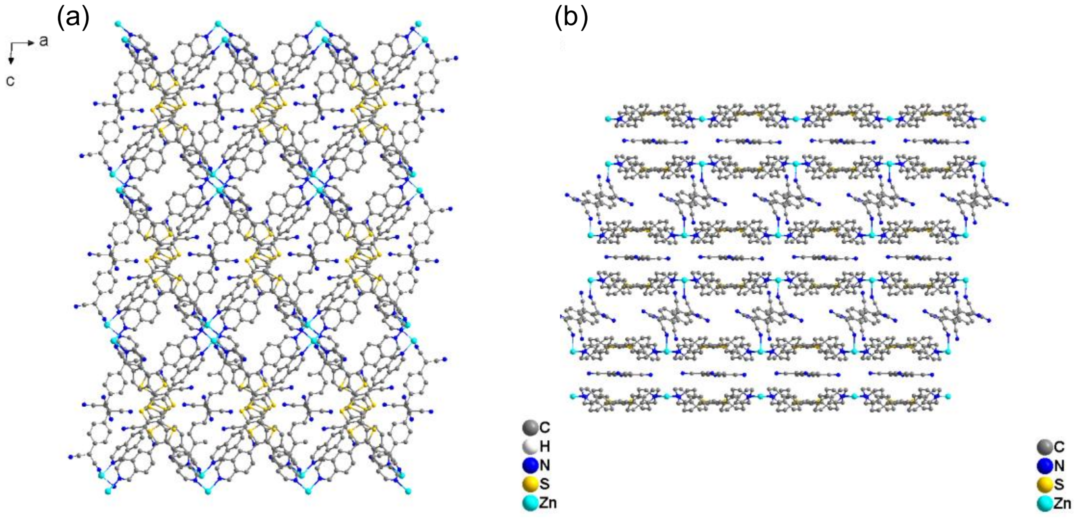

Supplementary Fig. 4. Schematic structure diagram of NJUZ-Cd. (a) The 2D layered structure of NJUZ-Cd is viewed along the b, c axis. (b) The 3D layered structure of NJUZ-Cd.

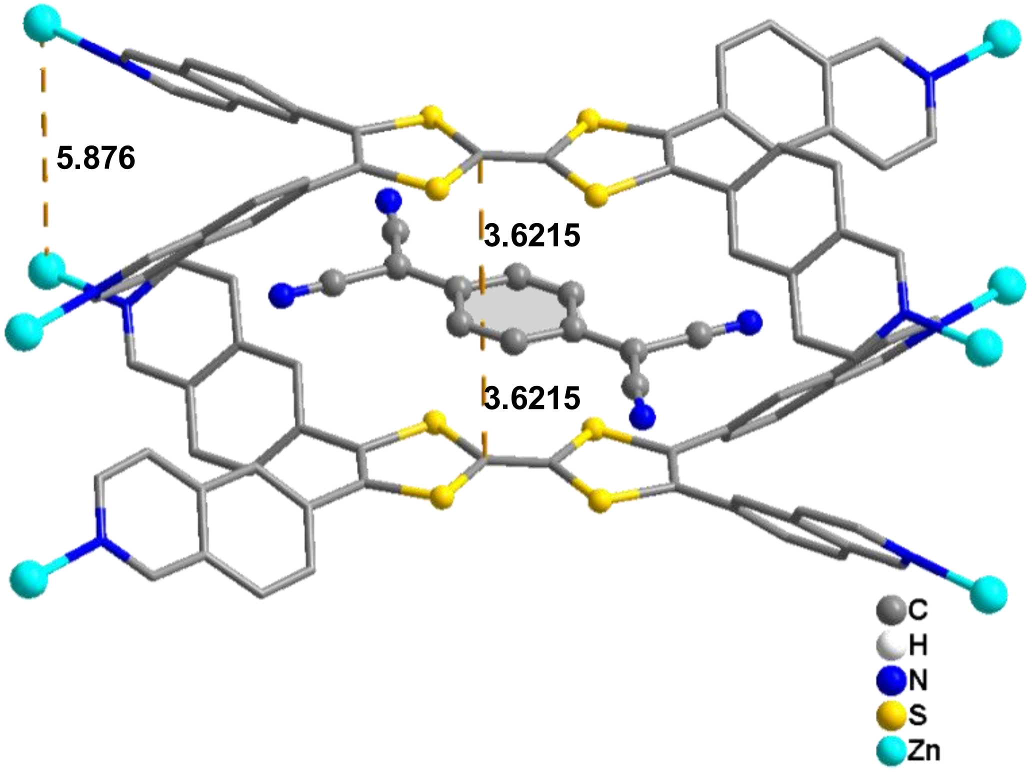

Supplementary Fig. 5. The interactions of π···π stacking in the cage of $[(L)_2(Zn_2)_4]$.

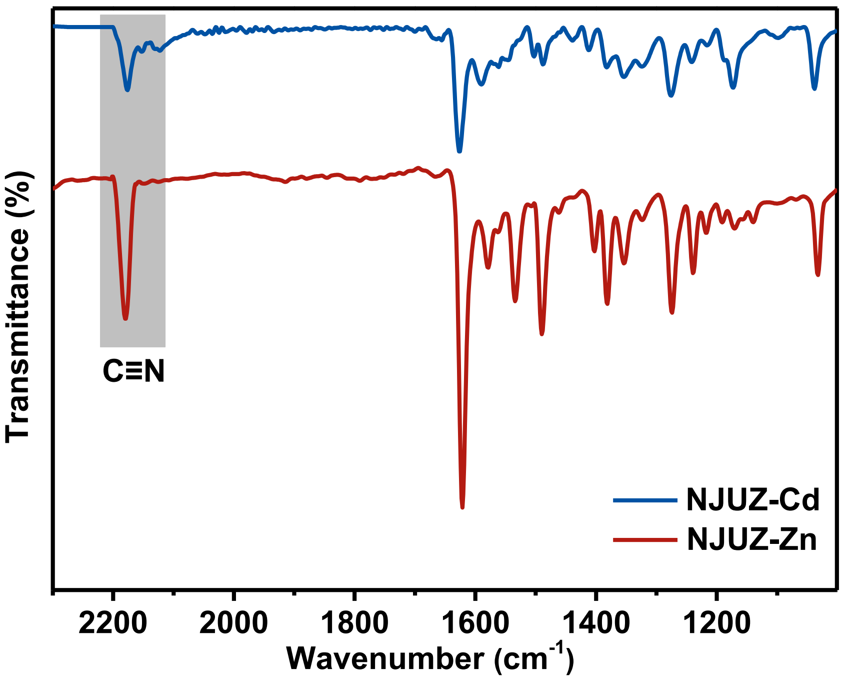

Supplementary Fig. 6. IR spectrum of NJUZ–M (M = Zn, Cd).

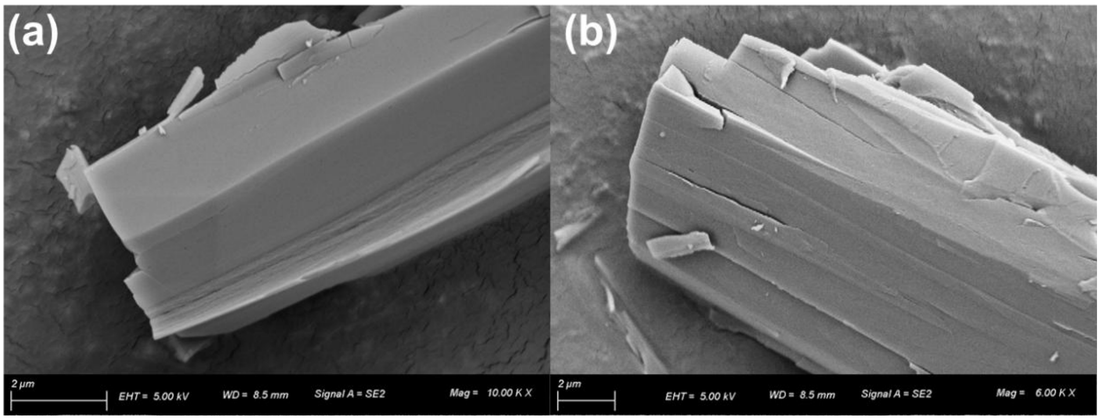

Supplementary Fig. 7. SEM images of (a) NJUZ-Zn, (b) NJUZ-Cd.

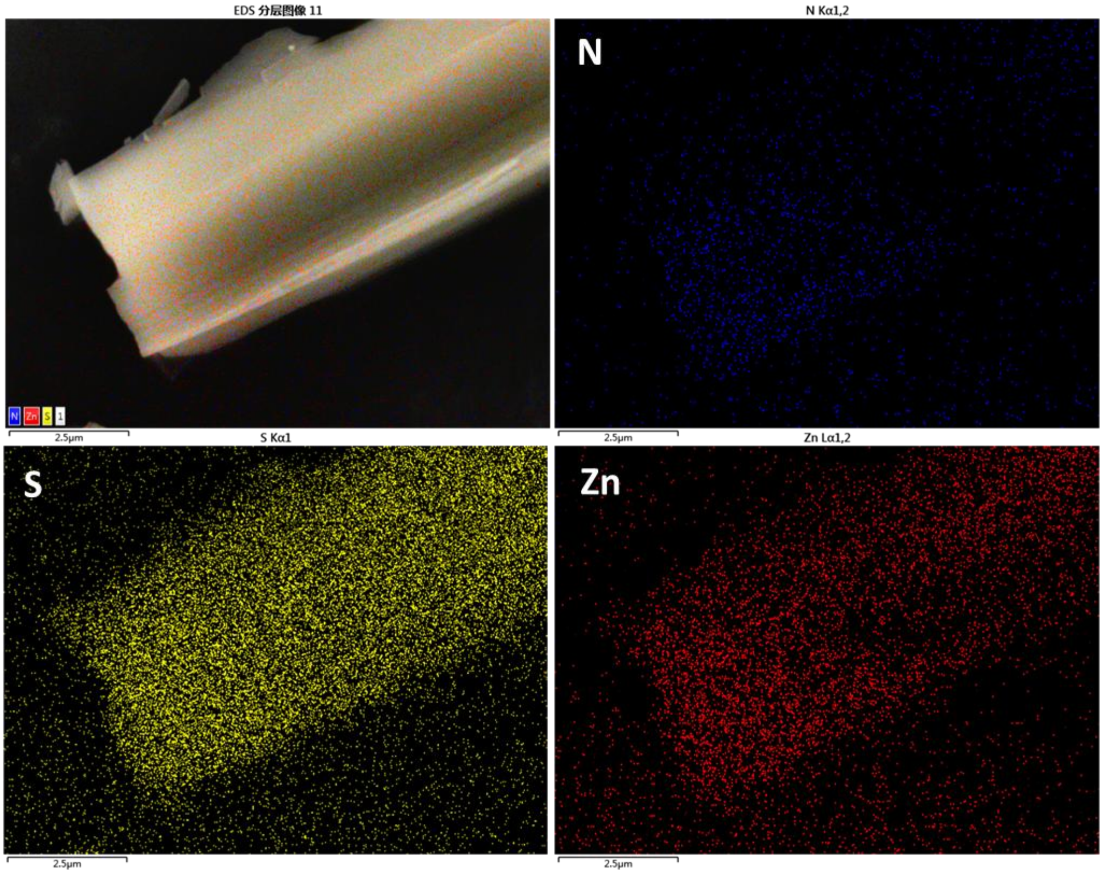

Supplementary Fig. 8. HAADF-STEM images and elemental mapping of NJUZ-Zn.

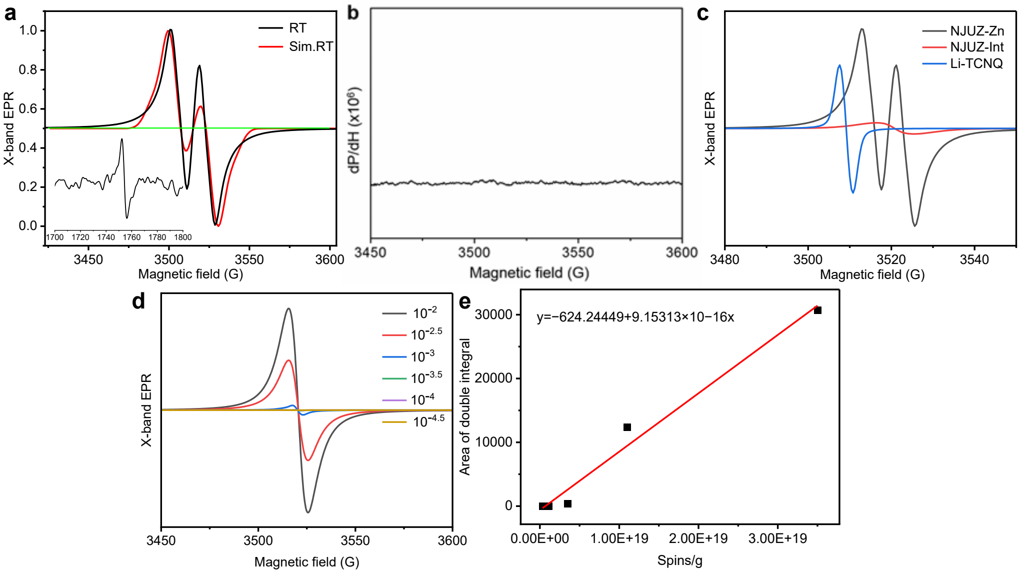

Supplementary Fig. 9. (a) Solid EPR spectrum of NJUZ-Zn measured at room temperature (RT, ν = 9.8564 GHz) compared with the simulated EPR spectrum. The forbidden transitions at the half-field and simulated spectrum are shown as inserts. (b) Solid EPR spectrum of L measured at room temperature. (c) Solid EPR spectrum of NJUZ-Zn, NJUZ-Int, Li-TCNQ measured at room temperature. (d) Room-temperature X-band EPR spectra of TEMPOL/KBr solid standards with different TEMPOL contents measured under the same instrumental conditions as the framework sample. (e) Linear calibration curve obtained by plotting the doubly integrated EPR area against the corresponding spin concentration of the TEMPOL/KBr standards. The red line represents the linear fit used for quantitative spin calibration.

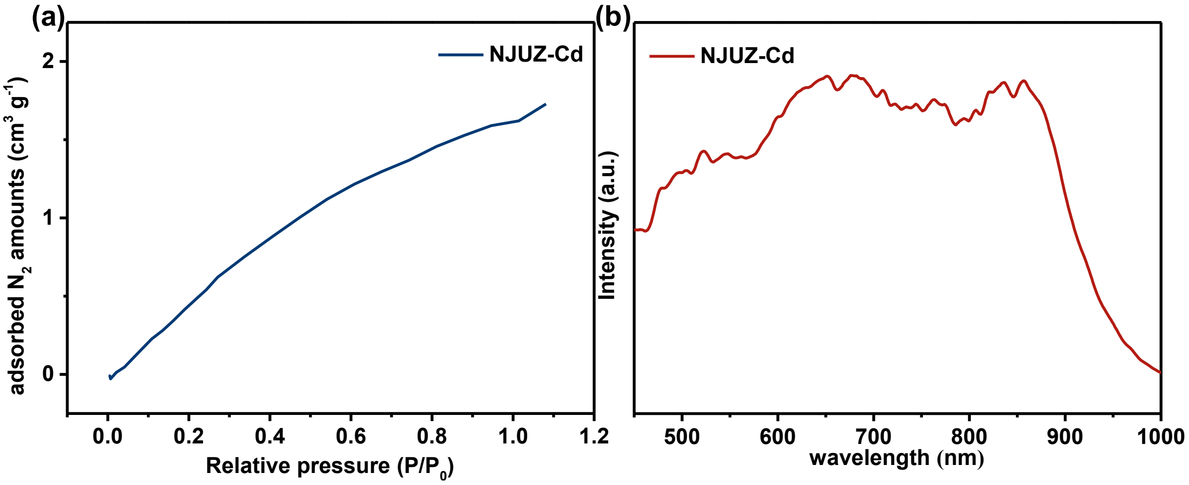

Supplementary Fig. 10. (a) N_2 adsorption isotherm of NJUZ-Cd at room temperature. (b) UV-vis absorption spectrum and fingertip aspects of NJUZ-Cd.

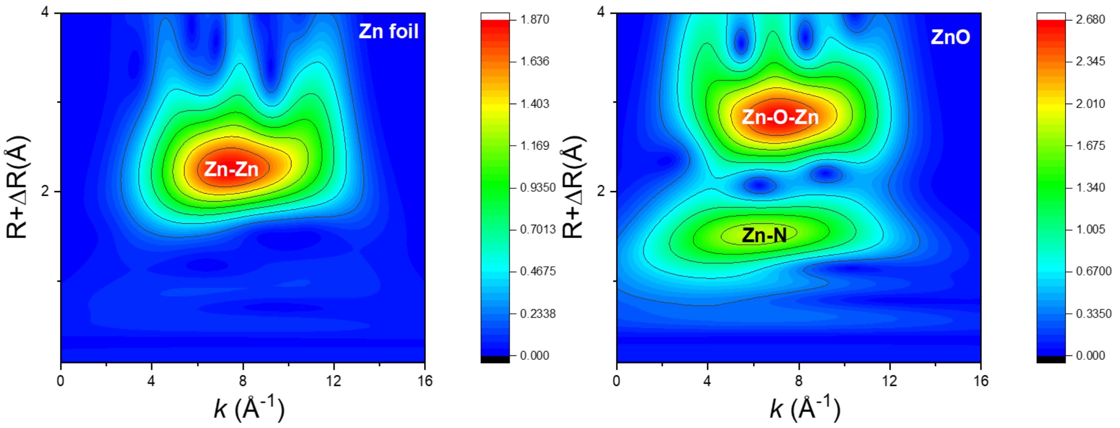

Supplementary Fig. 11. Wavelet Transformation for the k^3-weighted EXAFS signal of ZnO and Zn foil reference.

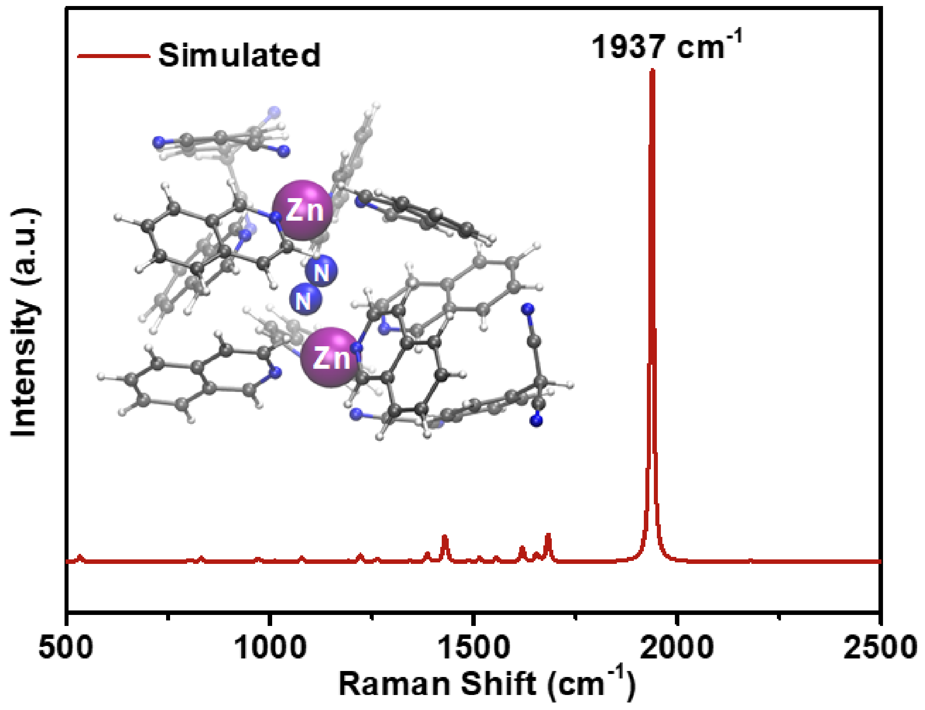

Supplementary Fig. 12. Simulated Raman spectrum of the Zn–N_2–Zn structure calculated using the DFT method at level1. The inset shows the Zn–N_2–Zn structure.

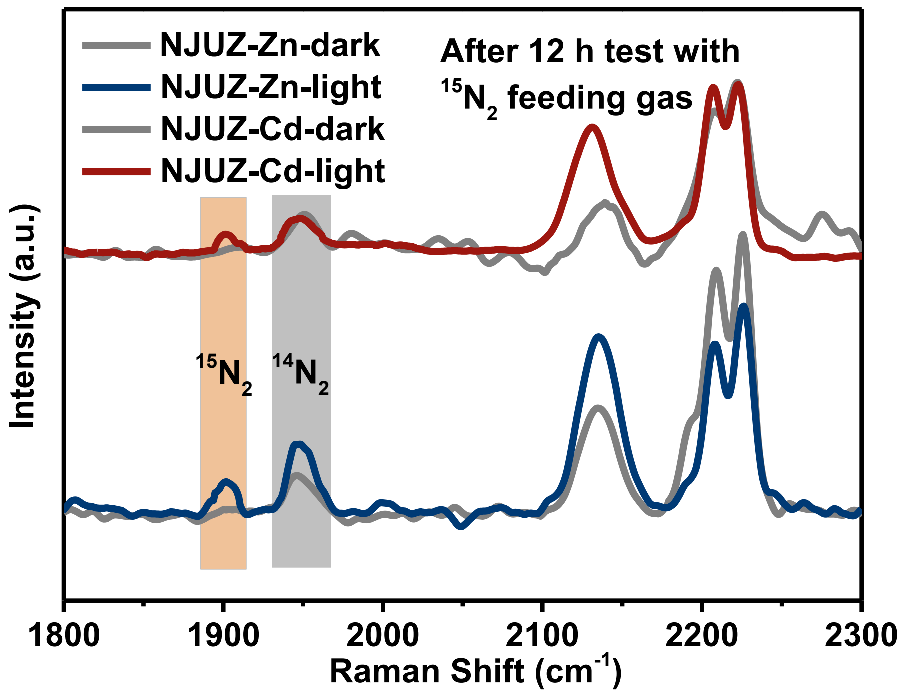

Supplementary Fig. 13. Comparative details of the Raman spectra for the N≡N bond of NJUZ-M (M = Zn, Cd) after catalytic reaction in both visible light and dark environments with $^{15}N_2$ feeding gas.

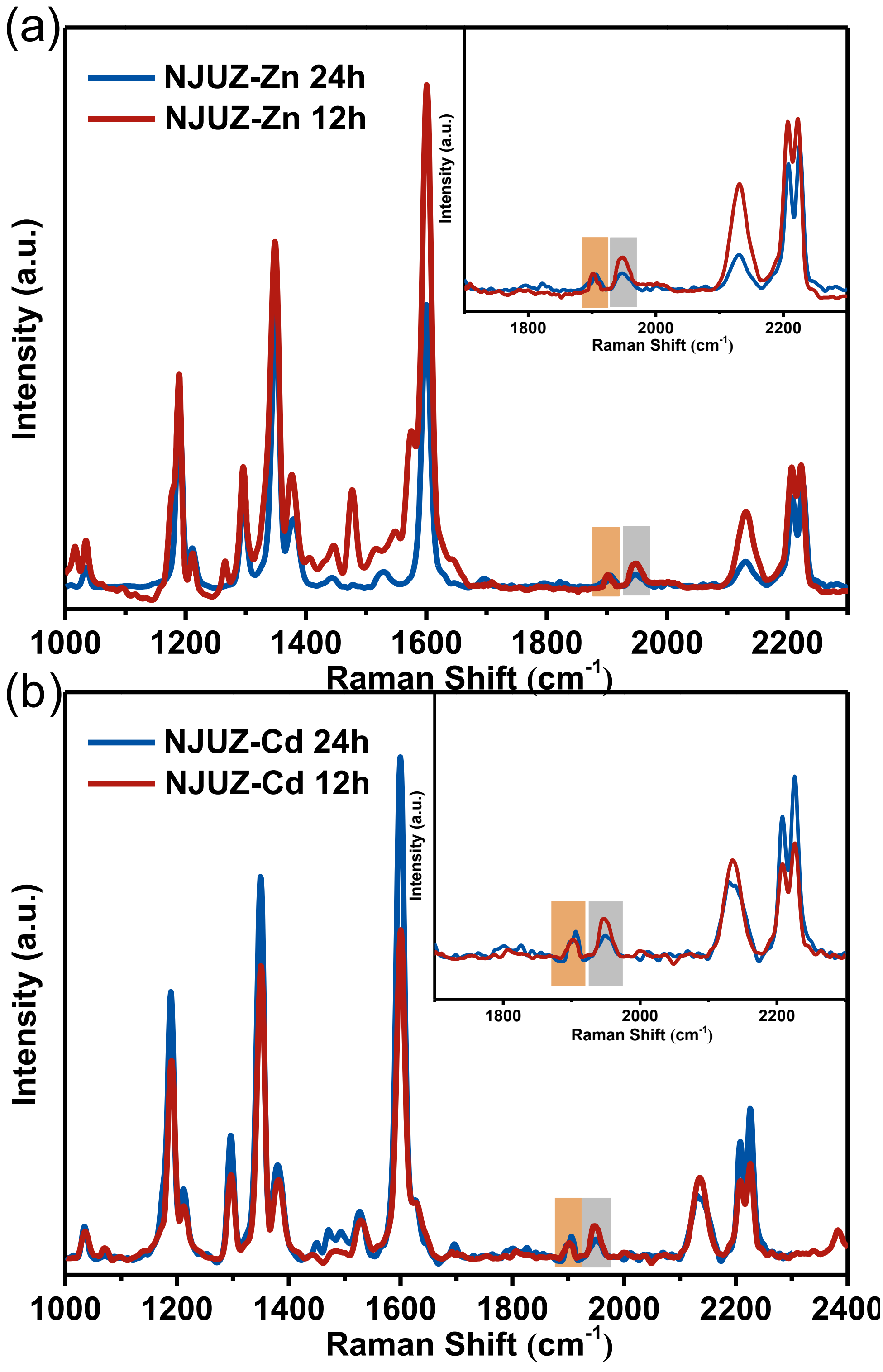

Supplementary Fig. 14. Comparison of Raman spectra after photocatalytic reaction in $^{15}N_2$ for 12 h and 24 h (a) NJUZ-Zn, (b) NJUZ-Cd. The inset shows the Raman spectra of the M–N_2–M structure at different reaction times.

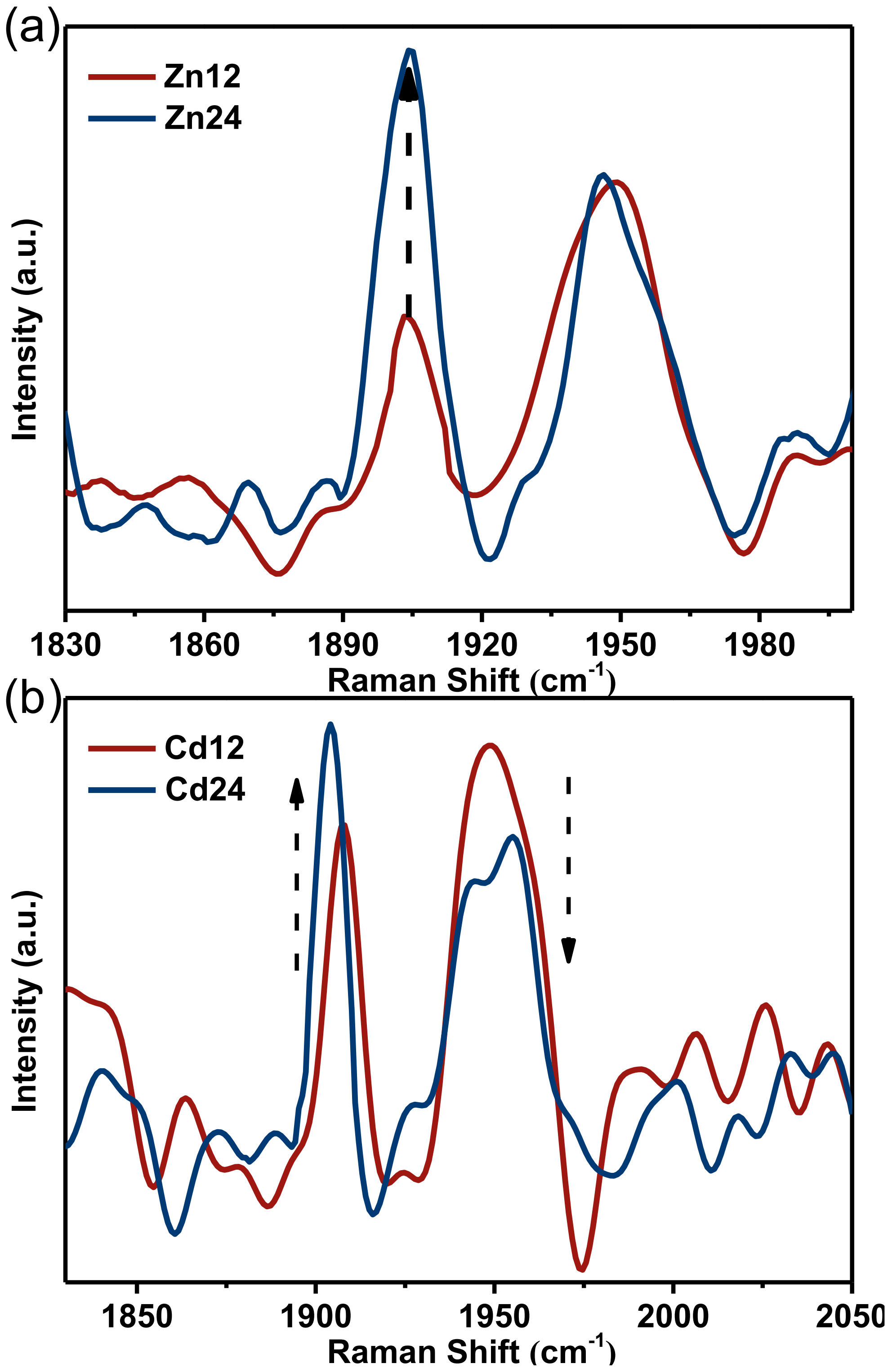

Supplementary Fig. 15. Raman spectra of the Zn–N_2–Zn and Cd–N_2–Cd at different reaction times in $^{15}N_2$ atmosphere.

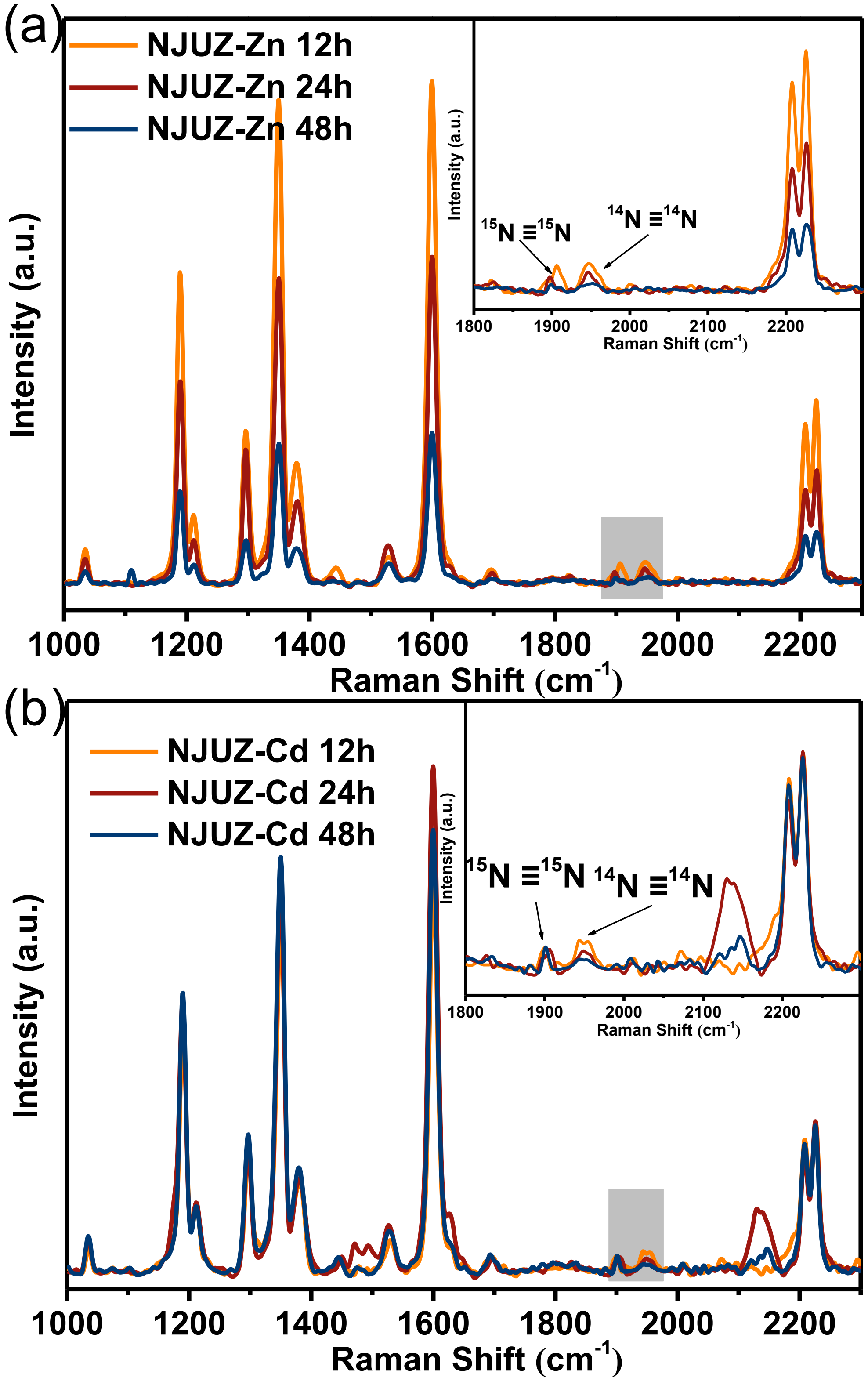

Supplementary Fig. 16. Comparison of Raman spectra after photocatalytic reaction in $^{15}N_2$ for 12 h, 24 h, and 48 h (a) NJUZ-Zn, (b) NJUZ-Cd. The inset shows the Raman spectra of the M–N_2–M structure at different reaction times.

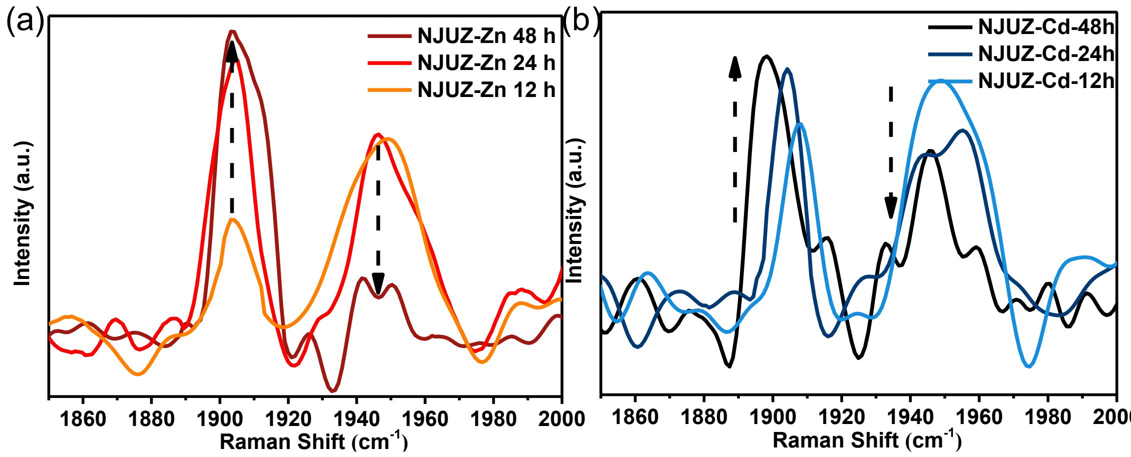

Supplementary Fig. 17. Comparative details of the Raman spectra for the N≡N bond of NJUZ-M (M = Zn, Cd) after photocatalytic reaction in $^{15}N_2$ for 12 h, 24 h, and 48 h.

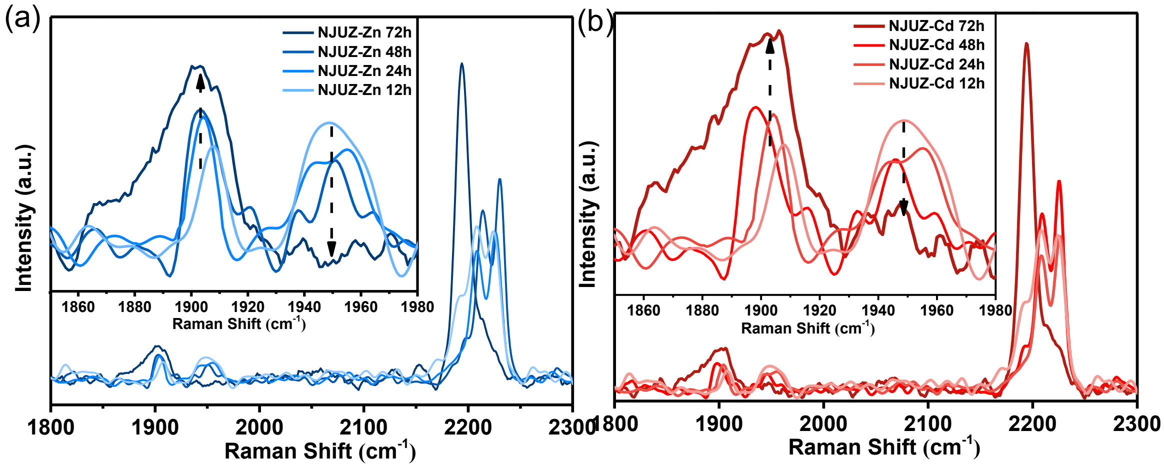

Supplementary Fig. 18. Comparative details of the Raman spectra for the N≡N bond of NJUZ-M (M = Zn, Cd) after photocatalytic reaction in $^{15}N_2$ for 12 h, 24 h, 48 h, and 72 h.

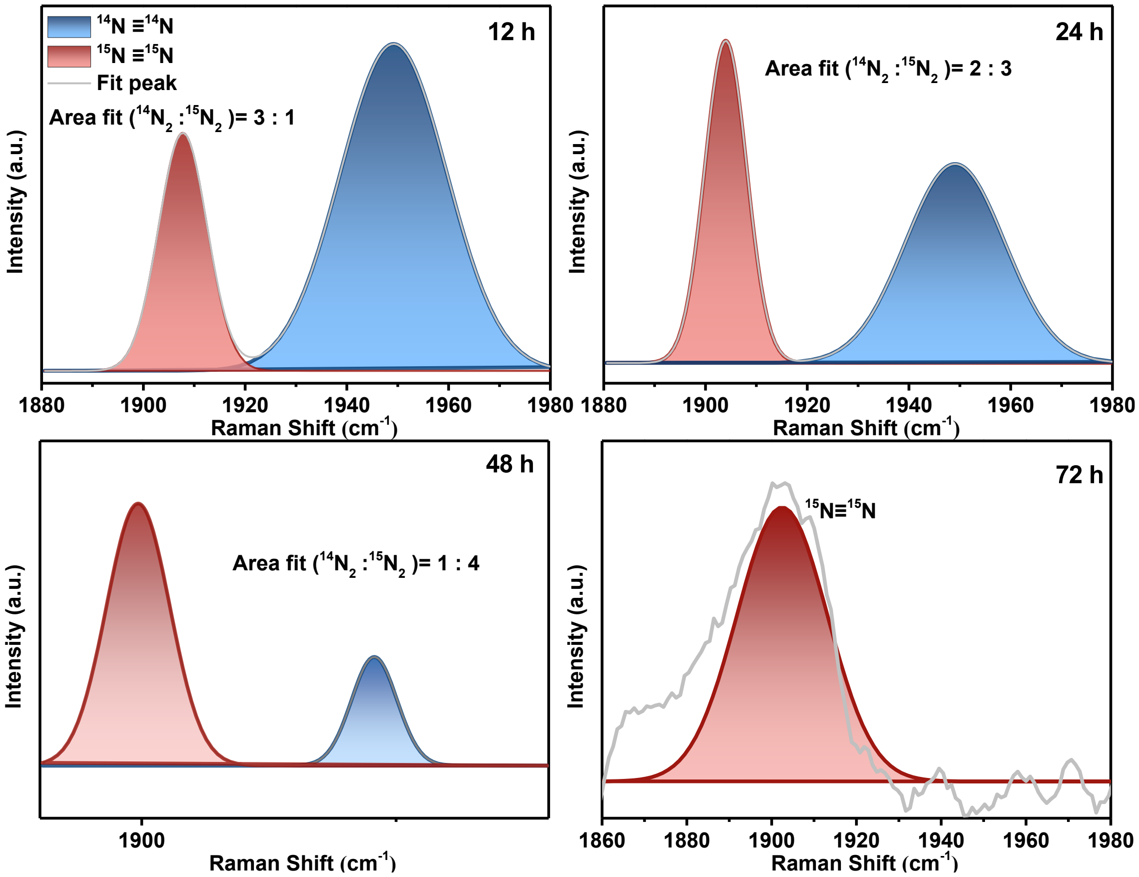

Supplementary Fig. 19. The spectra after fitting of the Raman spectra of N≡N bonds tested at different reaction times (NJUZ-Zn).

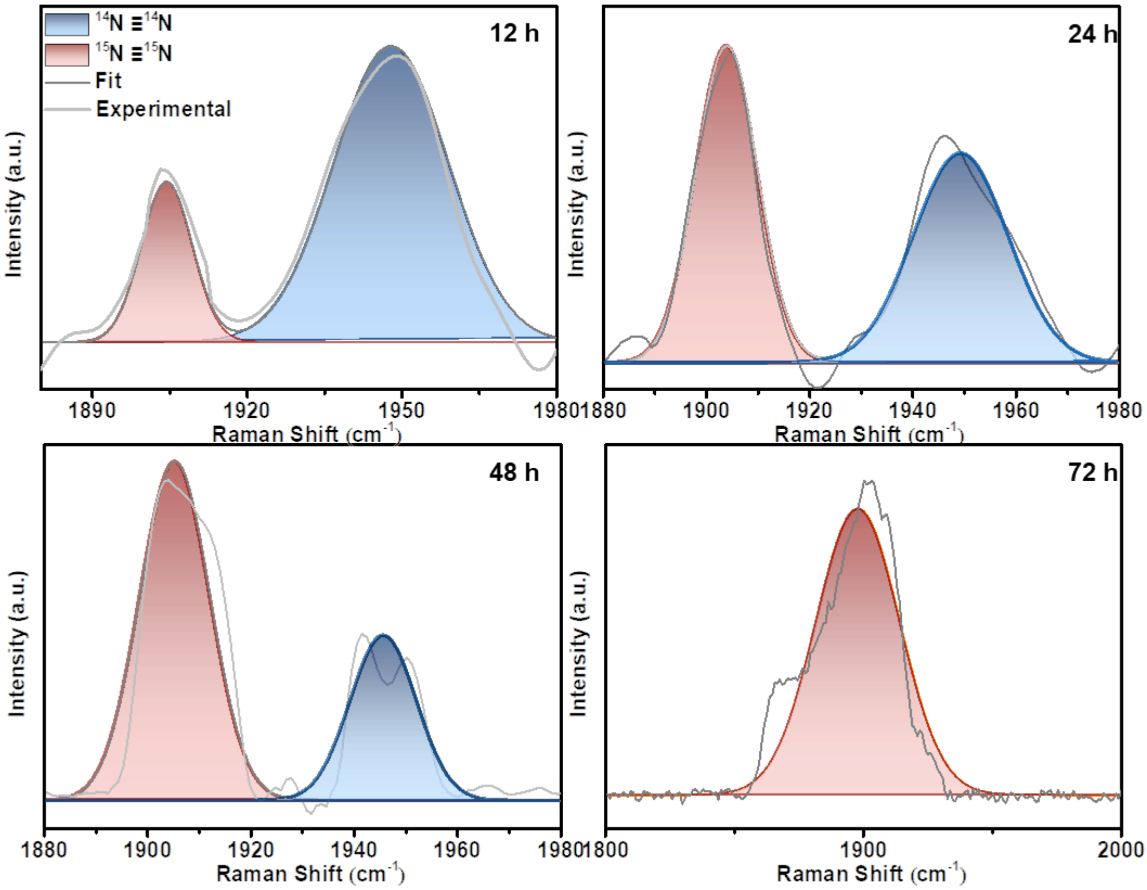

Supplementary Fig. 20. The spectra after fitting of the Raman spectra of N≡N bonds tested at different reaction times (NJUZ-Cd).

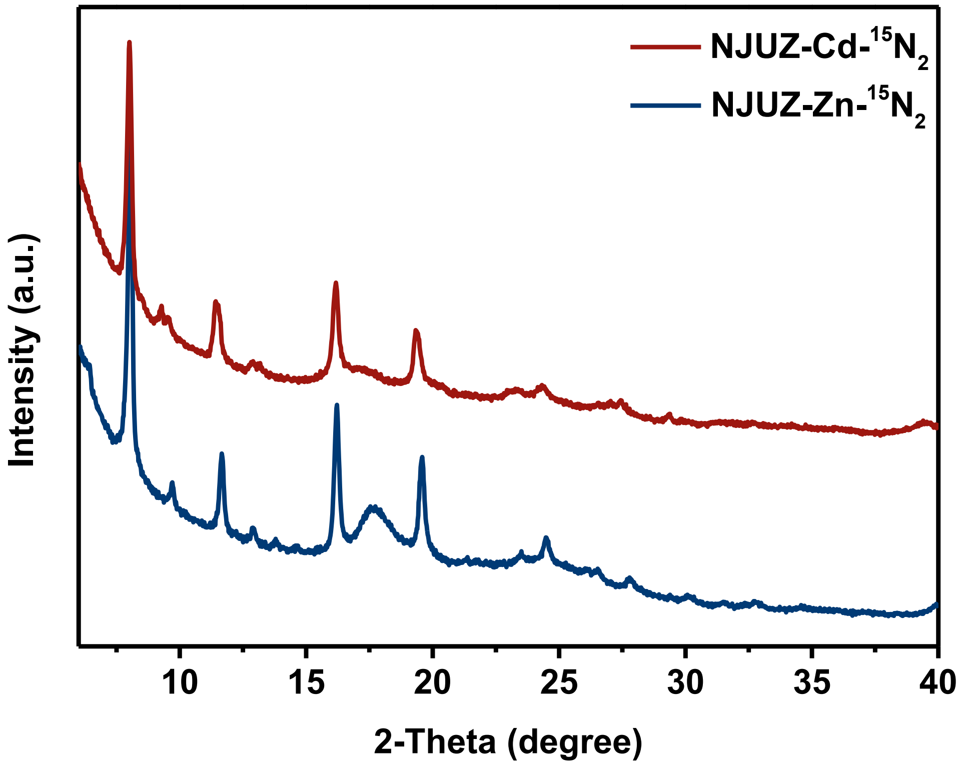

Supplementary Fig. 21. XRD patterns of NJUZ–Zn-$^{15}N_2$ and NJUZ–Cd-$^{15}N_2$.

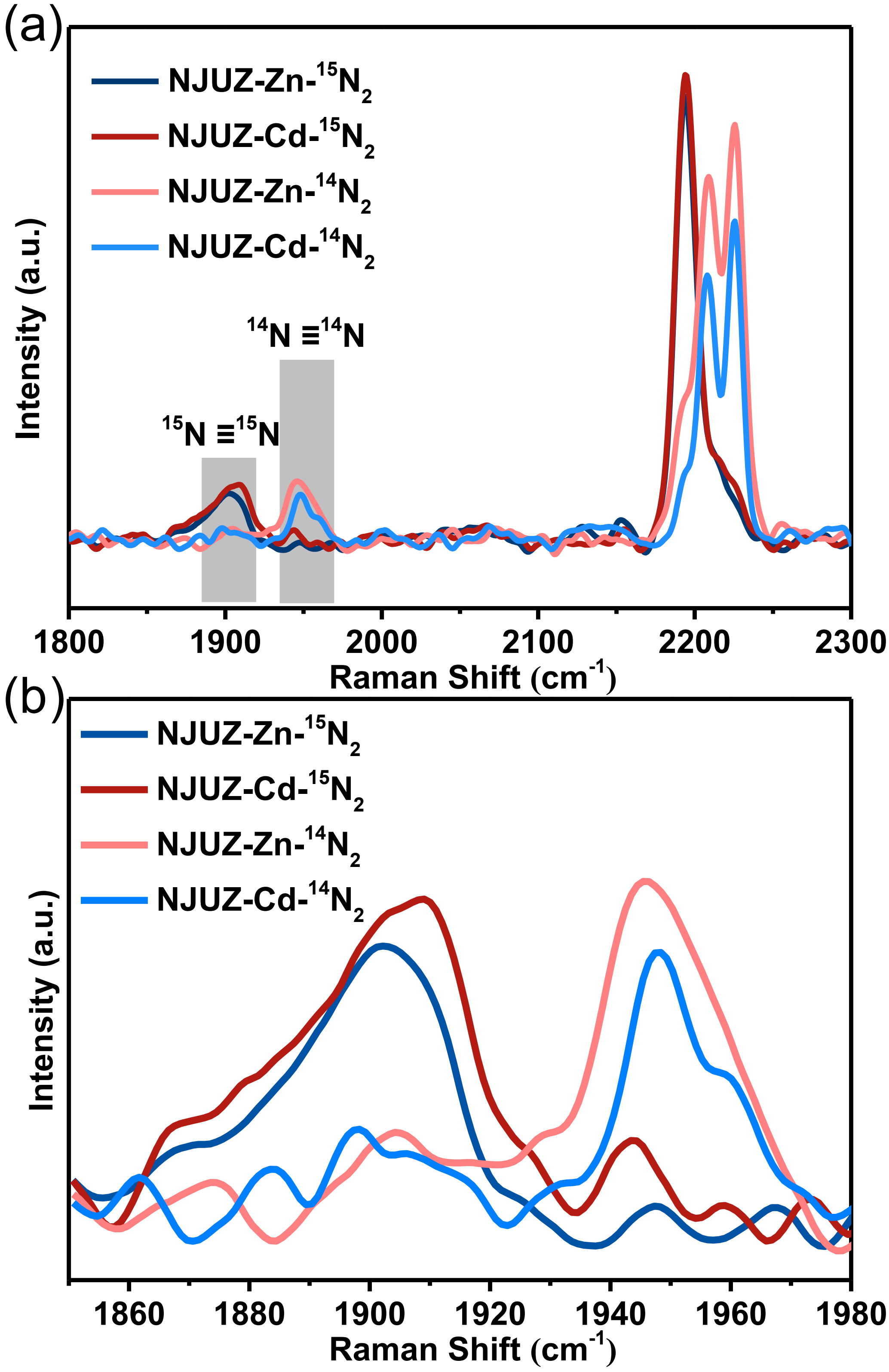

Supplementary Fig. 22. Raman spectra of Zn-N_2-Zn and Cd-N_2-Cd in single crystals grown under different conditions.

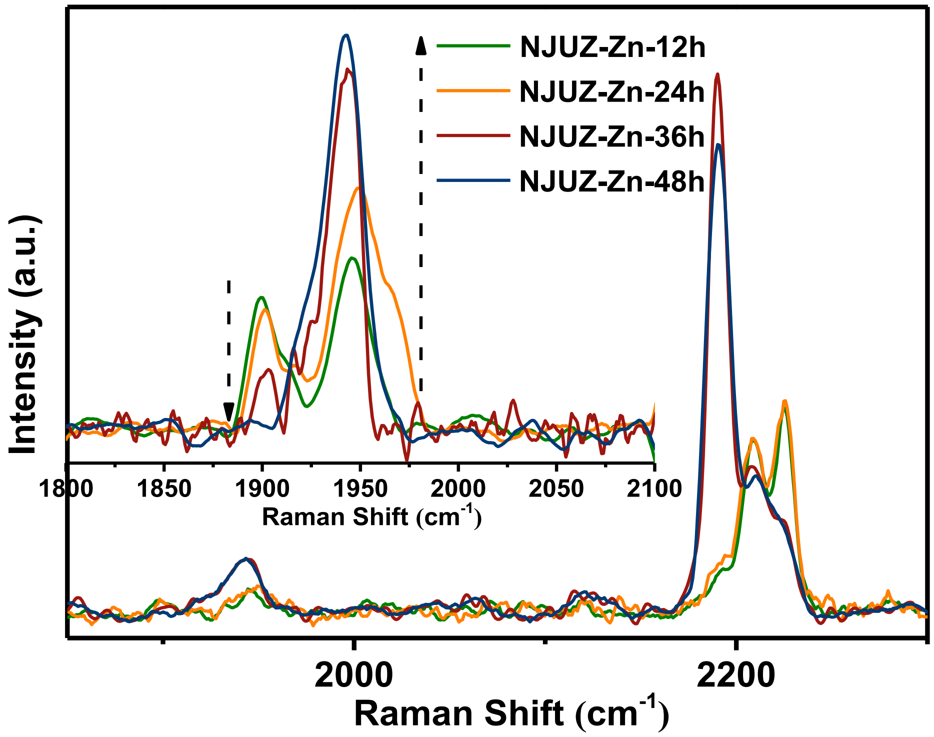

Supplementary Fig. 23. Comparative details of the Raman spectra for the N≡N bond of NJUZ-M (M = Zn) after photocatalytic reaction in $^{14}N_2$ for 12 h, 24 h, 36 h, and 48 h.

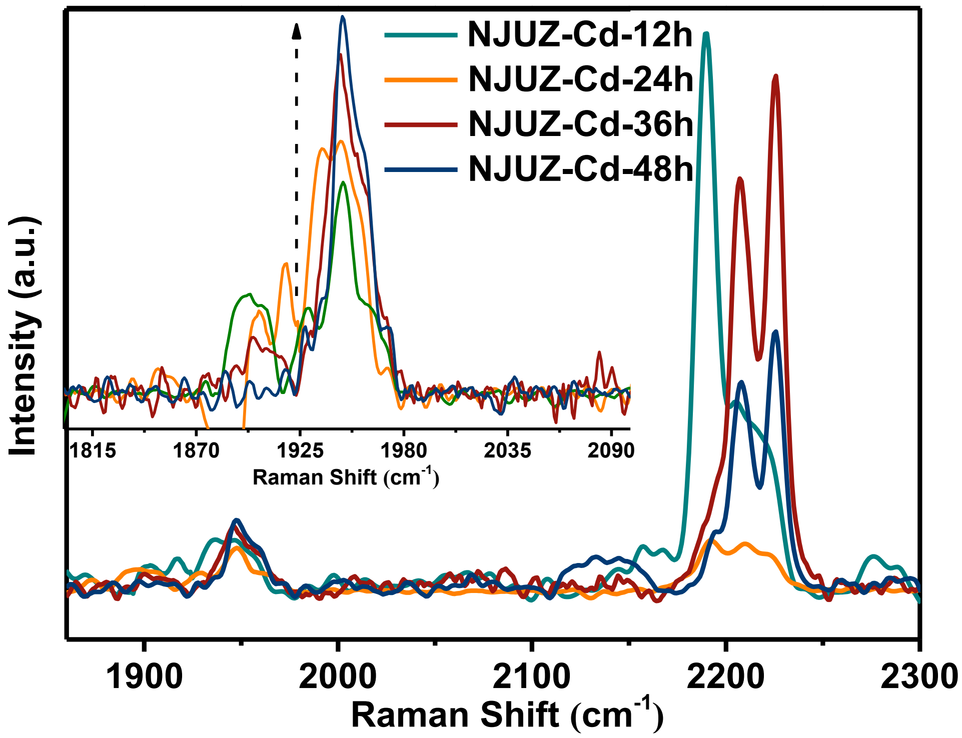

Supplementary Fig. 24. Comparative details of the Raman spectra for the N≡N bond of NJUZ-M (M = Cd) after photocatalytic reaction in $^{14}N_2$ for 12 h, 24 h, 36 h, and 48 h.

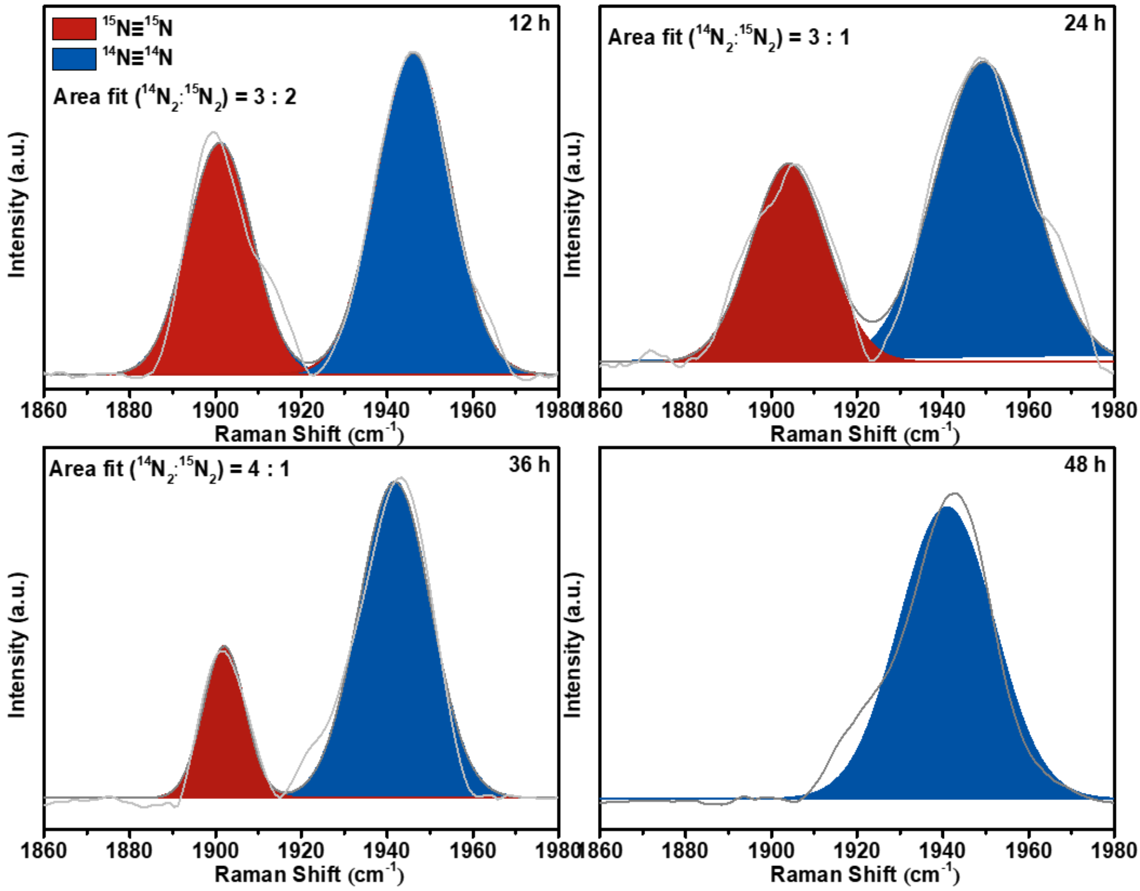

Supplementary Fig. 25. The spectra after fitting of the Raman spectra of N≡N bonds tested at different reaction times in $^{14}N_2$ atmosphere (NJUZ-Zn).

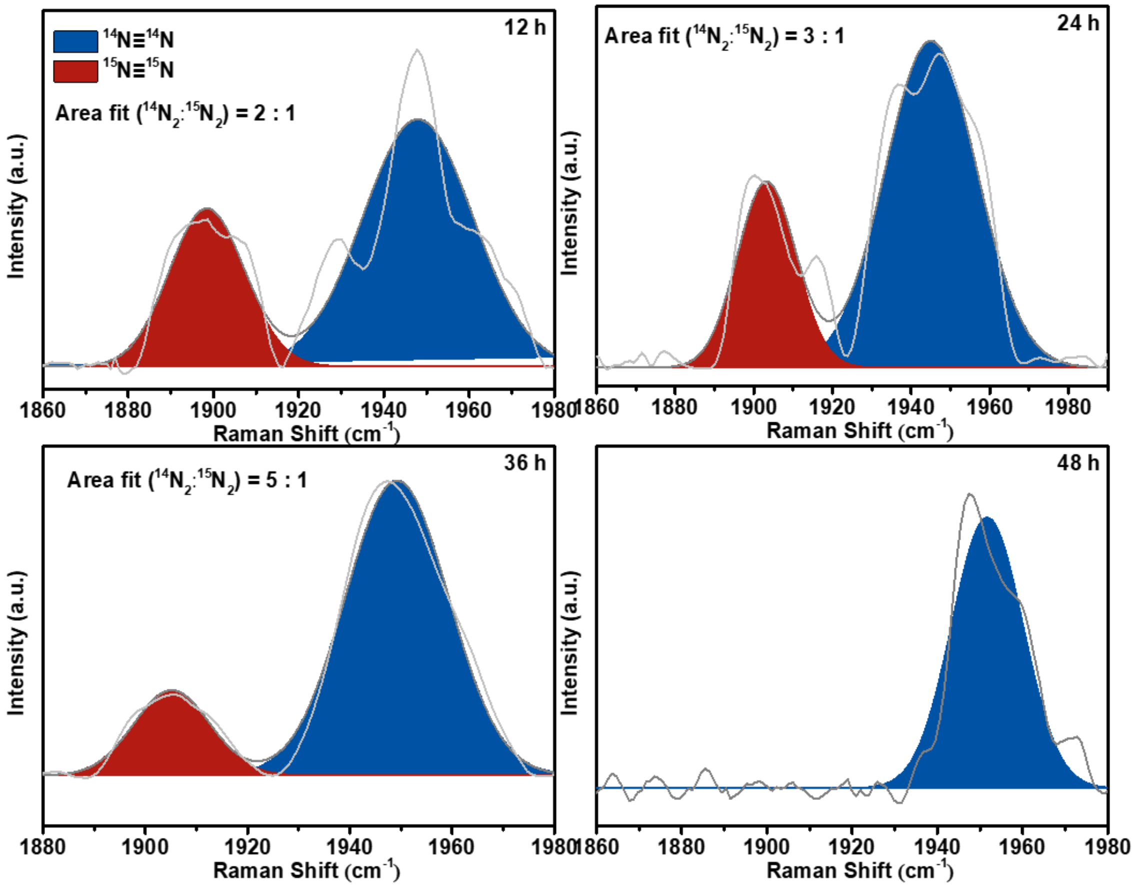

Supplementary Fig. 26. The spectra after fitting of the Raman spectra of N≡N bonds tested at different reaction times in $^{14}N_2$ atmosphere (NJUZ-Cd).

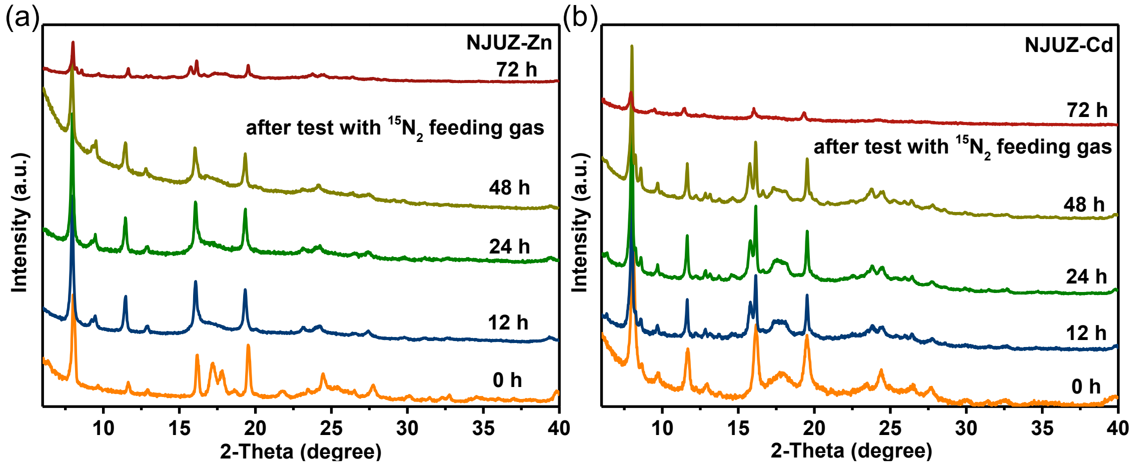

Supplementary Fig. 27. XRD patterns of NJUZ–Zn and NJUZ–Cd after different reaction times in $^{15}N_2$ atmosphere.

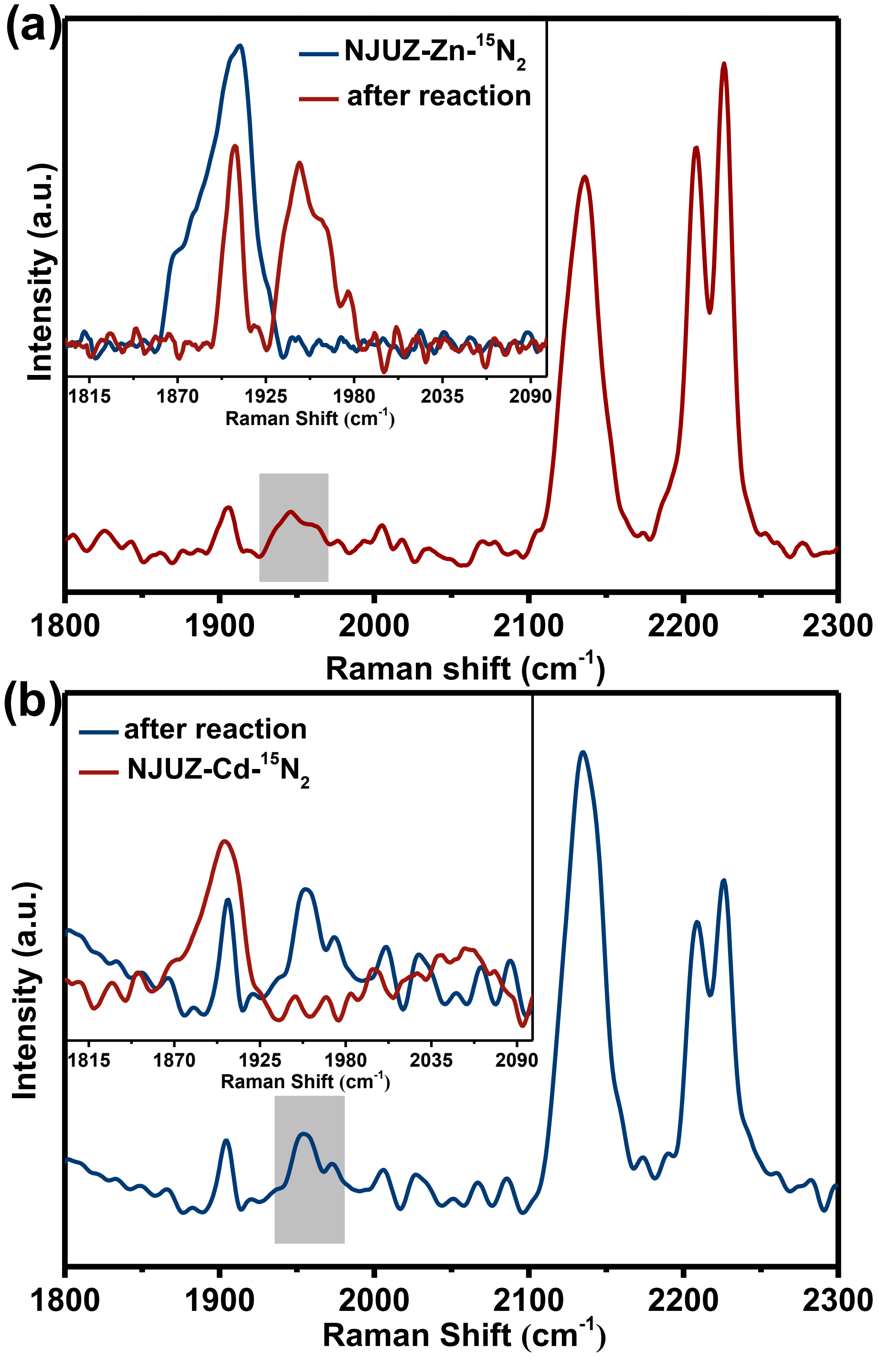

Supplementary Fig. 28. Comparative details of the Raman spectra for the N≡N bond of NJUZ-M (M =Zn, Cd) after photocatalytic reaction in unpurified air flow.

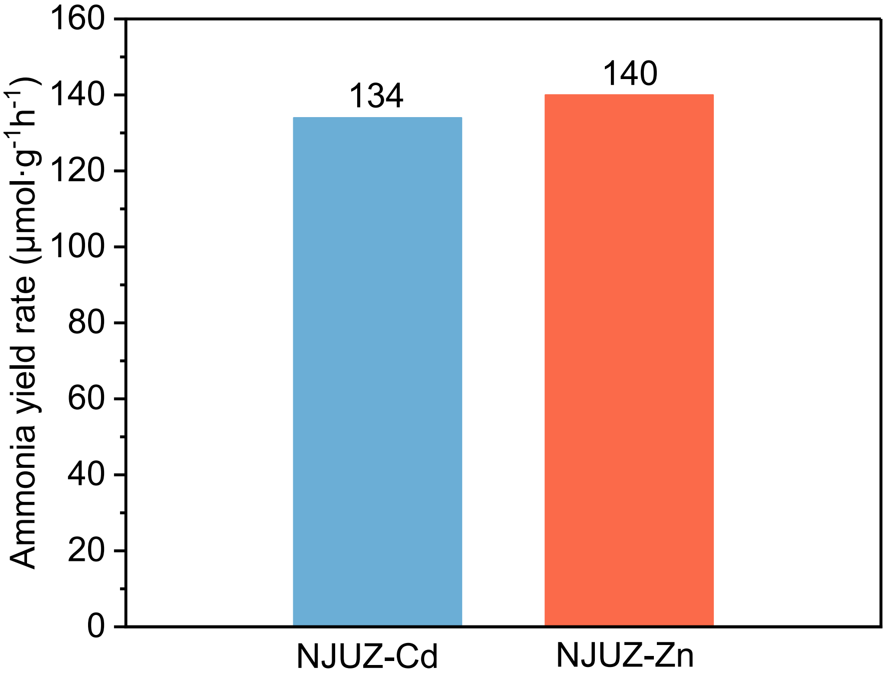

Supplementary Fig. 29. Nitrogen reduction reaction (NRR) was carried out on both NJUZ-Zn and NJUZ-Cd under nitrogen flow.

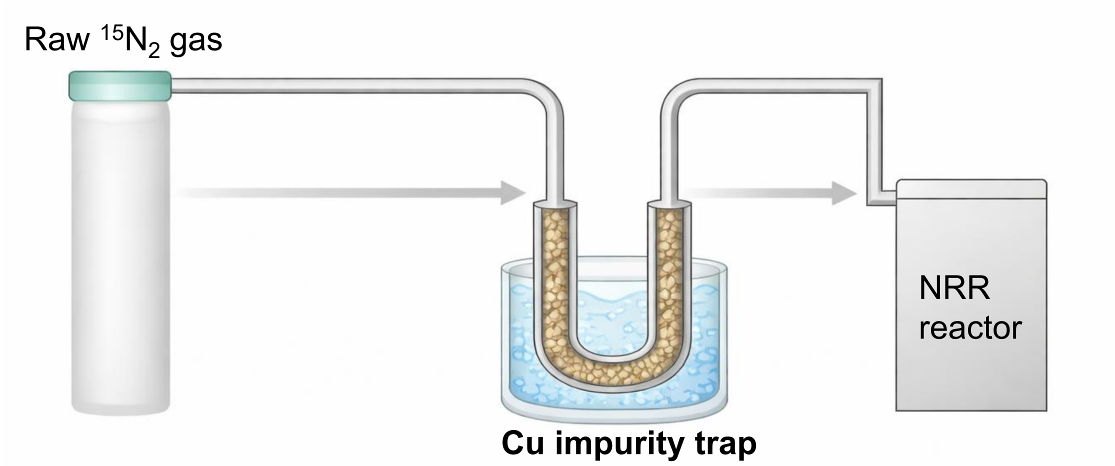

Supplementary Fig. 30. Schematic illustration of the purification of $^{15}N_2$ before the NRR experiment. The gas was passed through a Cu impurity trap consisting of a U-shaped stainless-steel tube packed with Cu–Zn–Al oxide catalyst. The trap was pre-reduced in 5% H_2/Ar at 300 °C for 2 h, then purged with Ar at 300 °C for 30 min, and cooled to −100 °C using an ethanol/liquid nitrogen slurry before use.

Supplementary Table 1. crystal data and structure refinement parameters of **NJUZ-Cd** and **NJUZ-Int**.

Name	NJUZ-Cd	NJUZ-Int
Empirical formula	$C_{60}H_{30}CdN_{11}S_4$	$C_{60}H_{30}N_{10}S_4Zn$
Formula weight	1145.59	1084.55
Temperature/K	223.15	193.00
Crystal system	triclinic	triclinic
Space group	P-1	P-1
a/Å	12.5533(8)	12.2699(6)
b/Å	16.268(2)	16.4830(14)
c/Å	19.7709(11)	19.5674(9)
α/°	68.727(6)	69.209(5)
β/°	89.130(4)	88.991(4)
γ/°	81.363(7)	81.621(5)
V/Å^3	3716.4(6)	3657.7(4)
Z	2	2
ρ_{calc}/gcm^{-3}	1.024	0.985
μ/mm^{-1}	3.697	1.822
F(000)	1158.0	1108.0
Crystal size/mm^3	0.13 × 0.12 × 0.1	0.13 × 0.11 × 0.09
Radiation	CuKα (λ = 1.54178)	CuKα (λ = 1.54178)
2Θ range for data collection/°	5.902 to 133.19	4.834 to 136.486
Index ranges	-14 ≤ h ≤ 14, -18 ≤ k ≤	-14 ≤ h ≤ 14, -19 ≤ k ≤
Reflections collected	13044	13336
Independent reflections	13044	13336
Data/restraints/parameters	13044/146/705	13336/0/677
Goodness-of-fit on F^2	1.076	1.074
Final R indexes [I>=2σ(I)][a,b]	R1 = 0.0996, wR2 =	R1 = 0.0957, wR2 =
Final R indexes [all data][a,b]	R1 = 0.1493, wR2 =	R1 = 0.1618, wR2 =
Largest diff. peak/hole / e Å^{-3}	0.89/-0.66	0.67/-0.55

Supplementary Table 2. Selected bond lengths (Å) of **NJUZ-Cd**.

C1-C2	1.397	C33-N3	1.303(13)
C1-N1	1.275	C34-C35	1.411(14)
C2-C3	1.408	C34-C41	1.345(14)
C2-C7	1.418	C35-C36	1.373(11)
C3-C4	1.422	C36-C37	1.336(13)
C3-C19	1.380	C37-C38	1.439(14)
C4-C5	1.294	C37-C42	1.434(11)
C5-C6	1.425	C38-C39	1.435(14)
C5-C8	1.464	C38-C41	1.425(10)
C6-C7	1.385(12)	C39-C40	1.345(13)
C8-C9	1.326(10)	C40-N4	1.415(15)
C8-S2	1.753(7)	C42-N4	1.276(14)
C9-C10	1.496(10)	C43-C44	1.594(14)
C9-S1	1.748(7)	C43-N5	1.274(13)
C10-C11	1.396(15)	C44-C45	1.519(14)
C10-C15	1.432(15)	C44-C54	1.408(19)
C11-C12	1.425(11)	C45-C46	1.318(16)
C12-C13	1.406(14)	C45-C50	1.433(18)
C12-C17	1.330(16)	C46-C47	1.419(16)
C13-C14	1.376(15)	C47-C48	1.340(18)
C13-C16	1.412(13)	C48-C49	1.349(17)
C14-C15	1.382(12)	C48-C51	1.564(18)
C16-N2	1.291(16)	C49-C50	1.413(17)
C17-C18	1.400(12)	C51-C51#1	1.52(4)
C18-N2	1.356(16)	C51-C52	1.56(3)
C19-C20	1.346(12)	C51-C53	1.51(2)
C20-N1	1.399(13)	C52-N8	1.14(3)
C21-C22	1.309(10)	C53-N7	1.120(16)
C21-S1	1.737(7)	C54-N6	1.20(2)
C21-S2	1.763(7)	Cd1-N1	2.364(6)
C22-S3	1.795(7)	Cd1-N2#2	2.356(7)
C22-S4	1.745(7)	Cd1-N3#3	2.338(6)
C23-C24	1.325(11)	Cd1-N4#4	2.377(6)
C23-C34	1.488(10)	Cd1-N5	1.796(13)
C23-S2	1.768(7)	Cd1-N60	2.266(4)
C24-C25	1.479(10)	N60-N60#5	1.110(8)
C24-S4	1.795(7)	C55-C56	1.52(3)
C25-C26	1.311(13)	C55-N9	1.16(3)
C25-C32	1.351(13)	C56-C57	1.38(3)

C26-C27	1.419(11)	C56-C60	1.49(2)
C27-C28	1.369(16)	C56-C55A	1.53(3)
C27-C30	1.354(15)	C57-C58	1.413(16)
C28-C29	1.337(12)	C57-C59	1.406(14)
C29-N3	1.374(14)	C58-C59#6	1.34(2)
C30-C31	1.398(15)	C60-N10	1.190(19)
C30-C33	1.468(11)	N9A-C55A	1.17(3)
C31-C32	1.368(11)		

Symmetry transformations used to generate equivalent atoms:

^{1}2-X, 1-Y, -1-Z; ^{2}1+X, +Y, +Z; ^{3}1+X, +Y, -1+Z; ^{4}+X, +Y, -1+Z; ^{5}2-X, -Y, -Z; ^{6}1-X, -Y, 1-Z

Supplementary Table 3. Selected bond angles (°) of **NJUZ-Cd**.

N1-C1-C2	124.1(10)	C40-C39-C38	118.4(12)
C1-C2-C3	119.1(9)	C39-C40-N4	123.0(11)
C1-C2-C7	122.6(9)	C34-C41-C38	119.5(10)
C3-C2-C7	118.2(7)	N4-C42-C37	124.5(11)
C2-C3-C4	118.3(9)	N5-C43-C44	138.8(12)
C19-C3-C2	115.6(7)	C45-C44-C43	123.8(11)
C19-C3-C4	125.7(10)	C54-C44-C43	117.9(11)
C5-C4-C3	123.9(9)	C54-C44-C45	117.4(14)
C4-C5-C6	119.1(8)	C46-C45-C44	118.9(13)
C4-C5-C8	120.0(9)	C46-C45-C50	118.1(12)
C6-C5-C8	120.8(9)	C50-C45-C44	122.7(12)
C7-C6-C5	120.2(10)	C45-C46-C47	124.5(12)
C6-C7-C2	120.2(9)	C48-C47-C46	115.5(13)
C5-C8-S2	114.5(5)	C47-C48-C49	124.5(12)
C9-C8-C5	129.7(7)	C47-C48-C51	117.3(13)
C9-C8-S2	115.7(5)	C49-C48-C51	118.0(13)
C8-C9-C1	126.5(7)	C48-C49-C50	119.2(13)
C8-C9-S1	119.0(5)	C49-C50-C45	118.1(13)
C10-C9-S1	114.4(5)	C511-C51-C48	118(2)
C11-C10-C9	120.5(9)	C511-C51-C52	107.0(16)
C11-C10-C1	119.1(8)	C52-C51-C48	107.6(15)
C15-C10-C9	120.0(9)	C53-C51-C48	111.0(11)
C10-C11-C1	119.6(10)	C53-C51-C511	105.9(19)
C13-C12-C1	119.5(9)	C53-C51-C52	106.7(16)
C17-C12-C1	122.3(10)	N8-C52-C51	176(2)
C17-C12-C1	118.2(8)	N7-C53-C51	177(2)
C12-C13-C1	117.3(11)	N6-C54-C44	175(3)
C14-C13-C1	120.4(8)	N1-Cd1-N42	92.6(2)
C14-C13-C1	122.2(10)	N23-Cd1-N1	88.7(2)
C13-C14-C1	121.0(10)	N23-Cd1-N42	175.7(4)
C14-C15-C1	120.1(10)	N34-Cd1-N1	179.3(3)
N2-C16-C1	123.1(11)	N34-Cd1-N23	91.9(3)
C12-C17-C1	122.2(12)	N34-Cd1-N42	86.8(2)
N2-C18-C1	119.0(12)	N5-Cd1-N1	90.4(3)
C20-C19-C3	122.0(11)	N5-Cd1-N23	90.0(4)
C19-C20-N1	121.3(10)	N5-Cd1-N34	89.9(3)
C22-C21-S1	121.9(5)	N5-Cd1-N42	85.9(4)
C22-C21-S2	124.0(5)	N5-Cd1-N60	178.7(3)
S1-C21-S2	114.1(4)	N60-Cd1-N1	90.3(3)

C21-C22-S3	122.1(5)	N60-Cd1-N23	91.2(4)
C21-C22-S4	124.1(5)	N60-Cd1-N34	89.5(3)
S4-C22-S3	113.7(4)	N60-Cd1-N42	93.0(4)
C2-4C23-C3	129.5(6)	C1-N1-C20	117.6(7)
C24-C23-S3	116.7(5)	C1-N1-Cd1	126.7(7)
C34-C23-S3	113.8(5)	C20-N1-Cd1	115.4(6)
C23-C24-C2	127.8(7)	C16-N2-C18	120.0(8)
C23-C24-S4	118.1(5)	C16-N2-Cd15	118.6(7)
C25-C24-S4	114.1(5)	C18-N2-Cd15	121.0(8)
C26-C25-C2	120.7(8)	C29-N3-Cd16	117.8(6)
C26-C25-C3	115.5(7)	C33-N3-C29	116.5(7)
C32-C25-C2	123.7(9)	C33-N3-Cd16	125.1(7)
C25-C26-C2	122.4(9)	C40-N4-Cd17	118.8(7)
C28-C27-C2	124.8(10)	C42-N4-C40	118.9(8)
C30-C27-C2	118.1(9)	C42-N4-Cd17	121.9(7)
C30-C27-C2	116.8(9)	C43-N5-Cd1	168.0(9)
C29-C28-C2	124.3(12)	N608-N60-Cd1	173.3(9)
C28-C29-N3	121.1(12)	C21-S1-C9	95.0(3)
C27-C30-C3	122.3(8)	C8-S2-C21	95.9(3)
C27-C30-C3	117.3(10)	C23-S3-C22	96.0(3)
C31-C30-C3	120.3(10)	C22-S4-C24	95.5(3)
C32-C31-C3	112.5(10)	N9-C55-C56	171(3)
C25-C32-C3	128.6(10)	C57-C56-C55	120.5(17)
N3-C33-C3	123.8(9)	C57-C56-C60	124(2)
C35-C34-C2	120.3(9)	C57-C56-C55A	108(4)
C41-C34-C2	118.4(9)	C60-C56-C55	115(2)
C41-C34-C3	121.2(8)	C60-C56-C55A	113(6)
C36-C35-C3	119.8(10)	C56-C57-C58	127(2)
C37-C36-C3	120.7(10)	C56-C57-C59	116.4(17)
C36-C37-C3	121.0(8)	C59-C57-C58	116.3(19)
C36-C37-C4	122.8(10)	C599-C58-C57	122.4(19)
C42-C37-C3	116.1(9)	C589-C59-C57	120.9(16)
C39-C38-C3	119.0(8)	N10-C60-C56	174(3)
C41-C38-C3	117.7(9)	N9A-C55A-C56	158(9)
C41-C38-C3	123.3(10)		

Symmetry transformations used to generate equivalent atoms:

[1]2-X, 1-Y, -1-Z; [2]+X, +Y, -1+Z; [3]1+X, +Y, +Z; [4]1+X, +Y, -1+Z; [5]-1+X, +Y, +Z; [6]-1+X, +Y, +Z, [7]+X, +Y, 1+Z, [8]2-X, -Y, -Z, [9]1-X, -Y, 1-Z

Supplementary Table 4. Elemental analysis of NJUZ-Cd, and NJUZ-Int.

sample	**N（%）**	**C（%）**	**H（%）**	**S（%）**
**NJUZ-Cd**	**9.417**	**58.578**	**2.601**	**12.622**
**NJUZ-Int**	**9.174**	**58.475**	**2.488**	**12.377**

Supplementary Table 5. EXAFS fitting parameters at the Zn K-edge of NJUZ-Zn, and NJUZ-Int SACs catalyst.

**Condition**	**Path**	***CN***	***R* (Å)**	**σ^2 (10^{-3}Å^2)**	**ΔE_0 (eV)**	R factor
Zn foil	Zn-Zn	6	2.63	10.8	-0.1	0.010
NJUZ-Zn	Zn-N1	1.8	1.98	3.4	-0.9	0.018
	Zn-N2	3.9	2.15	8.3		
NJUZ-Int	Zn-N1	2.0	1.98	3.0	-1.1	0.012
	Zn-N2	3.1	2.15	4.5		

[a]CN is the coordination number for the absorber-backscatter pair, R is the average absorber-backscatter distance, σ^2 is the Debye-Waller factor, and ΔE_0 is the inner potential correction. *S_0^2 was fixed to 0.87 as determined from Zn foil fitting. The accuracies of the above parameters are estimated as CN, ±20%; R, ±1%; σ^2, ± 20%; ΔE_0, ±20%. The data range used for data fitting in k-space (Δk) and R-space (ΔR) are 3.0-10.1 Å^{-1} and 0.8-2.4 Å, respectively.

Supplementary Table 6. Comparison of the catalytic performance of NJUZ-M and reported catalysts

Catalyst	Light source power	Ammonia yield	Ref.
**Gd-IHEP−8**	Xe lamp 300 W	220 μmol $h^{-1}g^{-1}$	*Angew. Chem. Int. Ed.,* 59 (2020), 20666-20671
**HMOF (Fe^{III}/Fe^{II})−10**	Xe lamp 300 W	164 μmol $h^{-1}g^{-1}$	*Chem. Eng. J.*, 443 (2022), 136559
**Ag@MIL−101(Cr)**	Xe lamp 300 W	138.81 μmol $g^{-1}h^{-1}$	*Catal. Sci. Technol.*, 13 (2023), 705-713.
**NJUZ-Zn**	Xe lamp 300 W	140 μmol $g^{-1}h^{-1}$	*Nat. Chem.* 15 (2023), 286–293.
**NJUZ-Cd**	Xe lamp 300 W	134 μmol $g^{-1}h^{-1}$	*This work*
**Al-PMOF(Fe)**	Xe lamp	127 μg $h^{-1}g^{-1}$	*ACS Nano,* 15 (2021), 9670-9678
**Gd-IHEP−7**	Xe lamp 300 W	128 μmol $h^{-1}g^{-1}$	*Angew. Chem. Int. Ed.,* 59 (2020), 20666-20671
**U(0.5Hf)−2SH**	Xe lamp 300 W	116.1 μmol $g^{-1}h^{-1}$	*Appl. Catal. B: Environ.*, 292 (2021), 120167
**Bi-MOF/g-C_3N_4**	Xe lamp	114.73 μmol $h^{-1}g^{-1}$	*J. Clean. Prod.,* 425 (2023), 138912
**MIL−125@TiO_2**	Xe lamp 300 W	102.7 μmol $h^{-1}g^{-1}$	*J. Alloy. Compd.*, 909 (2022), 164751
**UiO−66-visible light**	Xe lamp 300 W	95 μmol $h^{-1}g^{-1}$	*Nanoscale,* 13 (2021), 7801-7809
**Ti_3C_2-QD/Ni-MOF**	Xe lamp 300 W	88.79 μmol $g^{-1}h^{-1}$	*ACS Sustain. Chem. Eng.,* 8 (2020), 17791-17799
**Keggin-type POM@MIL−101(Cr)**	Xe lamp 300 W	75.56 μmol $h^{-1}g^{-1}$	*J. Colloid Interface Sci.*, 621 (2022), 406-415
**Ru-MOF−74**	λ > 400 nm	70.9 μmol $h^{-1}g^{-1}$	*Surf. Interfaces,* 33 (2022), 102225

**JH Fe/TiO_2**	Xe lamp 300 W	56.87μmol $h^{-1}g^{-1}$	*Appl. Catal. B: Environ. Energy,* 347 (2024), 123795
**Ru_1/d-UiO−66**	Xe lamp 300 W	53.28 μmol $h^{-1}g^{-1}$	*Angew. Chem. Int. Ed.,* 63 (2023), e202314408
**MIL−101(Fe)**	Xe lamp 300 W	50.355 μmol $L^{-1}h^{-1}$	*J. Solid State Chem.*, 285 (2020), 121245
**Fe-abtc**	Xe lamp 300 W	49.8 μmol $h^{-1}g^{-1}$	*J. Colloid Interface Sci.*, 633 (2023), 703-711
**(1:1) CeO_2/UNH (Ce)**	Hg lamp 250 W	47.55 μmol $L^{-1}h^{-1}$	*ACS Appl. Nano Mater.*, 4 (2021), 9635-9652
**MIL−100(Fe)**	Xe lamp 300 W	46.532 μmol $L^{-1}h^{-1}$	*J. Solid State Chem.,* 285 (2020), 121245
**MIL−88(Fe)**	Xe lamp 300 W	40.035 μmol $L^{-1}h^{-1}$	*J. Solid State Chem.*, 285 (2020), 121245
**Zr-abtc**	Xe lamp 300 W	35.7 μmol $h^{-1}g^{-1}$	*J. Colloid Interface Sci.*, 633 (2023), 703-711
**MOF−76(Ce)**	Xe lamp 300 W	34 μmol $g^{-1}h^{-1}$	*ACS Appl. Mater. Interfaces,* 11 (2019), 29917-29923
**UiO−66$(SH)_2$−200**	Xe lamp 300 W	32.4 μmol $h^{-1}g^{-1}$	*Angew. Chem. Int. Ed.,* 61 (2022), e202117244
**Au@MOF**	Xe lamp 300 W	18.9 mmol $g^{-1}h^{-1}$	*J. Am. Chem. Soc.,* 143 (2021), 5727-5736
**NH_2-MIL−125(Ti)**	Xe lamp 300 W	12.3 μmol $g^{-1}h^{-1}$	*Appl. Catal. B: Environ.*, 267 (2020), 118686
**Graphene-embedded Ce-UiO−66**	LED light source (365 nm)	3.06 mmol L^{-1} (24 h)	*Angew. Chem. Int. Ed.*, 61 (2022), e202207026
**MOF@DF-C_3N_4**	Xe lamp 300 W	2.32 mmol $g^{-1}h^{-1}$	*RSC Adv.,* 10 (2020), 26246-26255
