# Supplementary material for: Organosilicon cluster goes ferroelectric
Source: Natl Sci Rev. 2026 Apr 29;13(14):nwag243. doi: 10.1093/nsr/nwag243 (PMC13411271; doi:10.1093/nsr/nwag243)
Supplement: nwag243_Supplemental_Files [file nwag243_supplemental_files.zip › Supporting_Information-359.pdf]

Supporting Information for

Organosilicon Cluster Goes Ferroelectric

Huan-Huan Chen,[*,a,†] *Shu-Wen Xiong,*[a,†] *Yan Qin,*[a] *Xian-Jiang Song,*[a] *Xiaomeng Liu,*[b] *Zhanpeng Wang,*[b] *Han-Yue Zhang*[*,b] *and Ren-Gen Xiong,*[*,a]

[a] Ordered Matter Science Research Center, Nanchang University, Nanchang 330031, People's Republic of China

[b] State Key Laboratory of Digital Medical Engineering, School of Biological Science and Medical Engineering, Southeast University, Nanjing, 211189, People's Republic of China.

Email: chenhh@ncu.edu.cn; xiongrg@seu.edu.cn; zhanghanyue@seu.edu.cn

†These authors contribute equally to this work

Methods

Crystal growth. AR (analytically regent) pure aminopropylisobutyl Si_8O_{12} (**1**) and isobutyl Si_8O_{12} were purchased from Adamas-beta (CAS number: 444315-15-5 and 221326-46-1). All reagents and solvents in the syntheses were of reagent grade and used without further purification. The colorless crystals of **1** and **2** were easily obtained by slow evaporation of its ethyl acetate/petroleum ether (1:1) mixture at room temperature.

Single-crystal X-ray crystallography and powder X-ray diffraction. Single-crystal X-ray diffraction data of **1** in the RTP and HTP were measured using a Rigaku Saturn 924 diffractometer with Mo-Kα radiation (λ = 0.71073 Å). Crystalclear software package was used to perform data processing, and crystal structures were resolved by direct method and continuous Fourier synthesis, with the refinement of full-matrix least-squares methods based on F2. The data collection and structure refinement of these crystals are summarized in **Table S1**. The X-ray crystallographic structures have been deposited at the Cambridge Crystallographic Data Centre (deposition numbers

CCDC 2494370-2494371, and 2498131-2498133) and can be obtained free of charge from the CCDC via www.ccdc.cam.ac.uk/getstructures. Variable-temperature powder X-ray diffraction (PXRD) measurements were performed on a Rigaku D/MAX 2000 PC X-ray diffractometer, and data were collected in the 2θ range of 5°–50° with a step size of 0.02°.

Differential scanning calorimetry and dielectric measurements. Differential scanning calorimetry (DSC) measurements were recorded on a NETZSCH DSC 200F3 instrument with a heating/cooling rate of 10 K min^{-1} under in aluminum crucibles at nitrogen atmosphere. The dielectric permittivity curves were measured on an automatic impedance Tonghui 2828 analyzer with an applied voltage of 1.0 V.

Second harmonic generation measurements. SHG measurement was conducted on a home-built confocal scanning microscope. A 1064 nm picosecond laser (Rainbow 1064 OEM, NPI lasers) and a femtosecond laser (10 W, 1030 ± 10 nm< 290 fs, PHAROS, Light Conversion) equipped with an optical parametric amplifier (ORPHEUS, Light Conversion) system are selected as the excitation sources. A 50X objective (N.A = 0.6, Nikon TU Plan ELWD) was selected to focus the laser onto the sample and collect the reflected SHG signals. The SHG signal was detected by a spectrograph (SpectraPro HRS-300, Teledyne Princeton Instruments). Temperature-dependent SHG measurement was performed using a liquid nitrogen cryostat (Instec, HCS621GXY). Polarization dependent SHG measurement was realized by rotating a half-wave plate on the excitation path.

Thin-film preparation. The thin films were prepared through a drop-casting method. 12 mg of the **1** crystals were dissolved in 0.6 mL ethyl acetate to prepare precursor solution, 20 μL of which was then spread on a clean indium-doped tin oxide (ITO)

glass substrate. The planar and compact thin film was obtained after annealing at 347 K for 0.5 hours.

PFM characterization. The PFM measurement was conducted using a commercial piezoresponse force microscope (Oxford Instrument, Cypher ES). This system consists of a high-voltage package, an in-situ heating stage, and custom-designed light sources.

***P*-*V* loop measurement.** The P-V hysteresis loop was measured using a semi-crystalline thin film capacitor. The ferroelectric hysteresis of the capacitor architecture (GaIn/sample film/ITO) was characterized by the double wave method.

Measurement of optical path difference. The optical path difference R of compound **1** was measured using an orthogonally polarized optical microscope equipped with a Berek compensator (OLYMPUS BX53-P). Monochromatic light with a wavelength of 550 nm was used during the measurement. The R value was determined by adjusting the compensator until the interference color in the selected region was compensated to extinction, following the standard Berek compensator procedure.

Spontaneous strain calculation. In both of the ferroelastic phase transition species of $3F1$, the relationship of crystal unit cells across phase transitions is shown in Figure S12. To ensure consistency of geometric relationship before and after phase transitions, the re-chosen unit cells in LTP and HTP present corresponding lattice parameters of (10.113, 11.138, 11.162, 93.844°, 100.347°, 90.922°) and (10.923, 10.923, 10.923, 95.223°, 95.223°, 95.223°), respectively. The spontaneous strain tensor in the general case is given by:

$$[e_{ij}] = \begin{bmatrix} e_{11} & e_{12} & e_{13} \\ 0 & e_{22} & e_{23} \\ 0 & 0 & e_{33} \end{bmatrix}$$

The components of the spontaneous-strain tensor as:

$$e_{11} = \frac{a\sin\gamma}{a_0\sin\gamma_0} - 1$$

$$e_{22} = \frac{b}{b_0} - 1$$

$$e_{33} = \frac{c\sin\alpha\sin\beta^*}{c_0\sin\alpha_0\sin\beta_0^*} - 1$$

$$e_{23} = \frac{1}{2}\left[\frac{c\cos\alpha}{c_0\sin\alpha_0\sin\beta_0^*} - \frac{b\cos\alpha_0}{b_0\sin\alpha_0\sin\beta_0^*} + \frac{\cos\beta_0^*}{\sin\beta_0^*\sin\gamma_0}\left(\frac{a\cos\gamma}{a_0} - \frac{b\cos\gamma_0}{b_0}\right)\right]$$

$$e_{13} = \frac{1}{2}\left(\frac{a\sin\gamma\cos\beta_0^*}{a_0\sin\gamma_0\sin\beta_0^*} - \frac{c\sin\alpha\cos\beta^*}{c_0\sin\alpha_0\sin\beta_0^*}\right)$$

$$e_{12} = \frac{1}{2}\left(\frac{a\cos\gamma}{a_0\sin\gamma_0} - \frac{b\cos\gamma_0}{b_0\sin\gamma_0}\right)$$

$$\varepsilon_{ss} = \left(\sum_{ij}\varepsilon_{ij}^2\right)^{\frac{1}{2}}$$

In the equations, the a, b, c, α, β, and γ represent the crystal lattice of the phase at lower temperature, and the a_0, b_0, c_0, α_0, β_0, and γ_0 represent the crystal lattice of the parent phase at higher temperature. β^* and β_0^* denote the reciprocal-lattice angles. Based on the cell parameters measured at 300 K and 347 K, the total spontaneous strain ε_{ss} was given as 0.097.

Nanoindentation method. A Bruker Hysitron TI Premier nanoindenter with a Berkovich probe (tip radius of 150 nm) was utilized to perform nanoindentation test to reveal elastic modulus (*E*) and hardness (*H*). The **1** single crystal plate with a smooth surface was adhered to a stainless iron disk by cyanoacrylate glue which could be fixed firmly onto the sample stage of the testing instrument by static magnetic attraction. Standard load function was set with peak load of 800 μN during the test. The *H* and *E* values were obtained by fitting the load-displacement curve based on the Oliver-Pharr method using the pre-installed software on the device. The

load-displacement data were collected from four different positions with distance of 10 μm to minimize test error.

TGA measurement. The TGA measurement was conducted using a Perkin-Elmer TGA 8000 instrument, with the sample heated at a rate of 30 K min^{-1} under a nitrogen atmosphere.

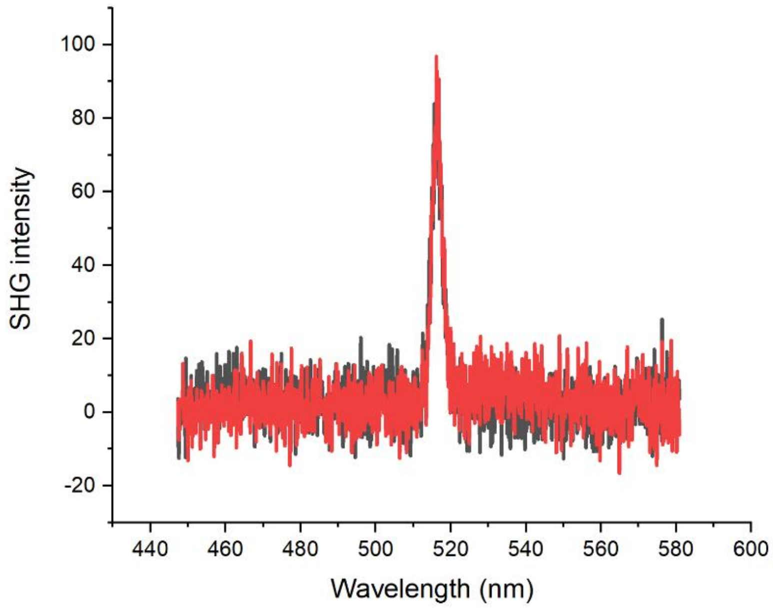

Figure S1. The SHG signal of compound **2**.

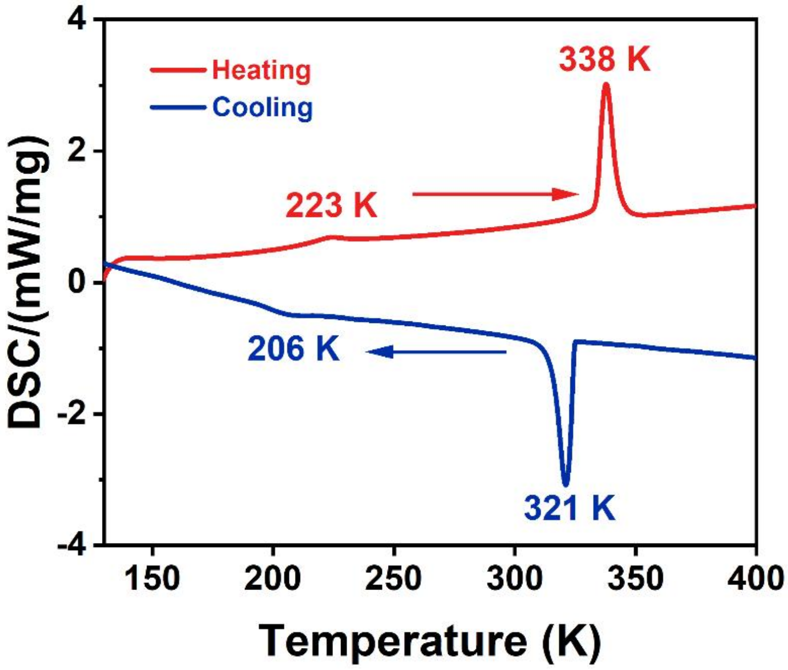

Figure S2. The DSC result of compound **2**.

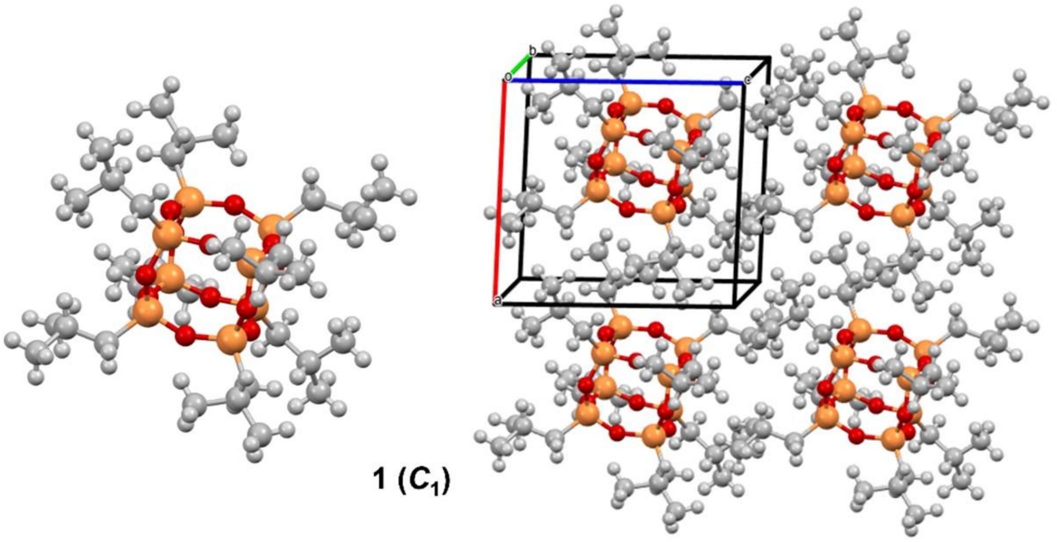

Figure S3. Crystal structure of compound **2** at 100 K.

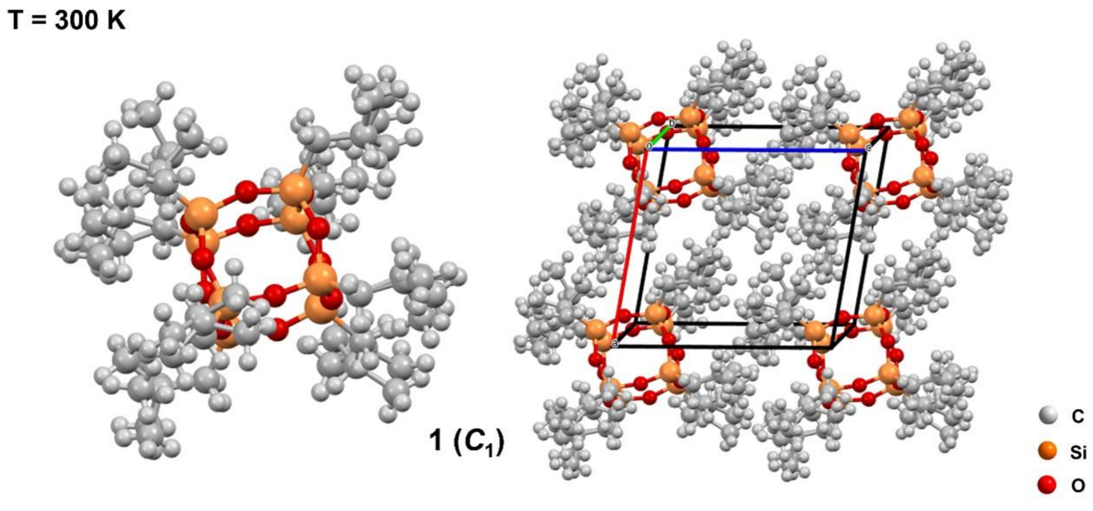

Figure S4. Crystal structure of compound **2** at 300 K.

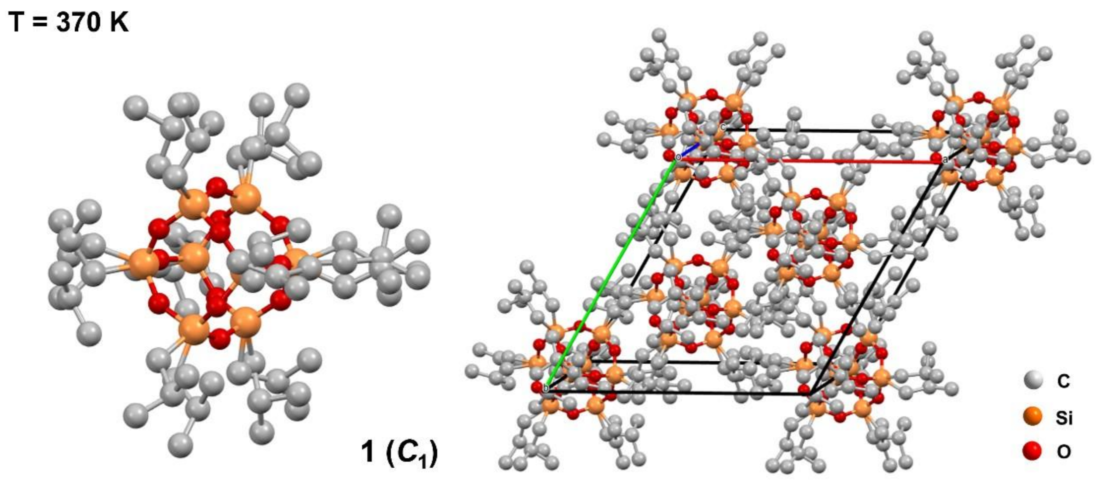

Figure S5. Crystal structure of compound **2** at 370 K.

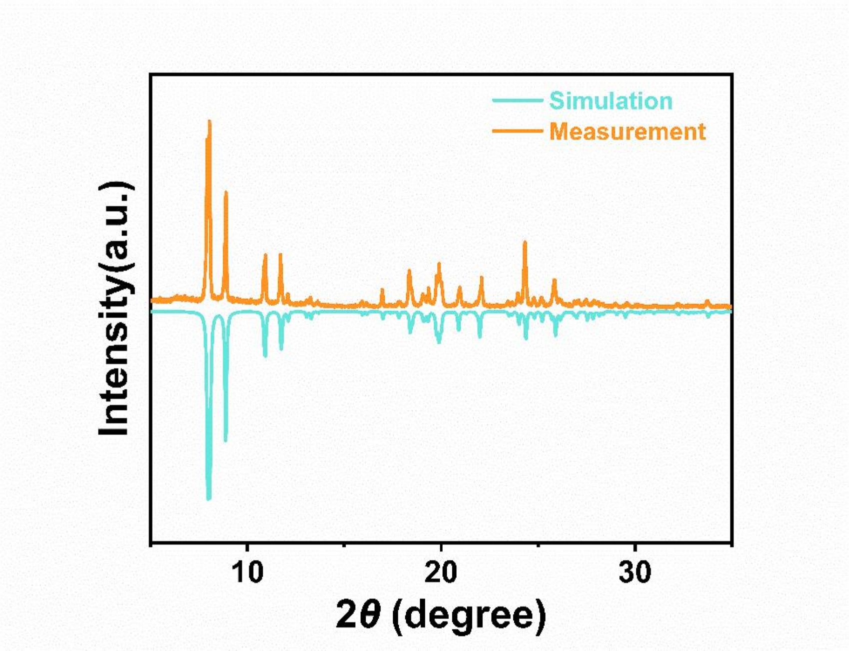

Figure S6. PXRD patterns of compound **1**.

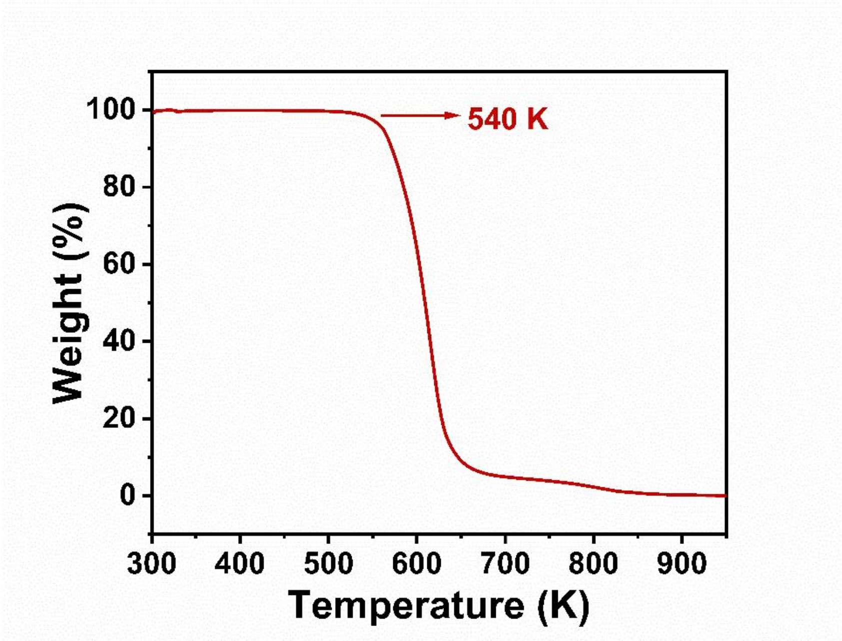

Figure S7. TGA curve of **1**.

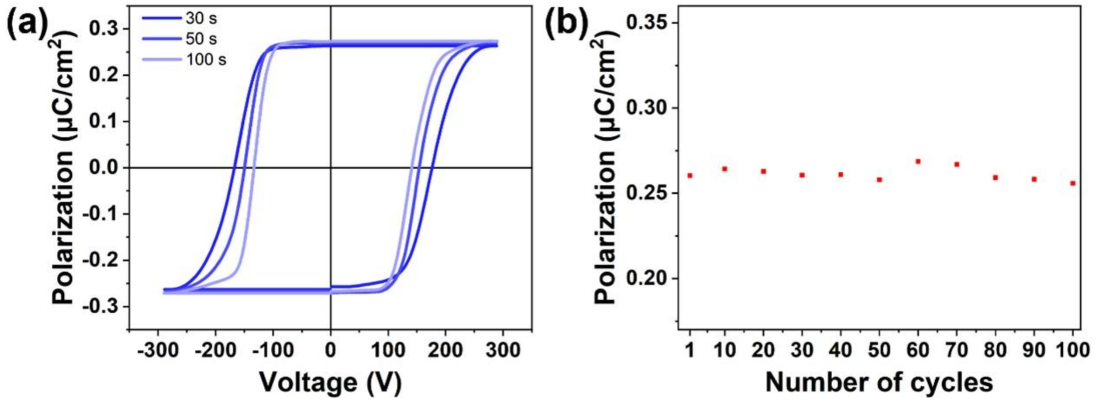

Figure S8. (a) Polarization-voltage (*P*-*V*) hysteresis loops under different testing periods and (b) cycling stability of saturation polarization.

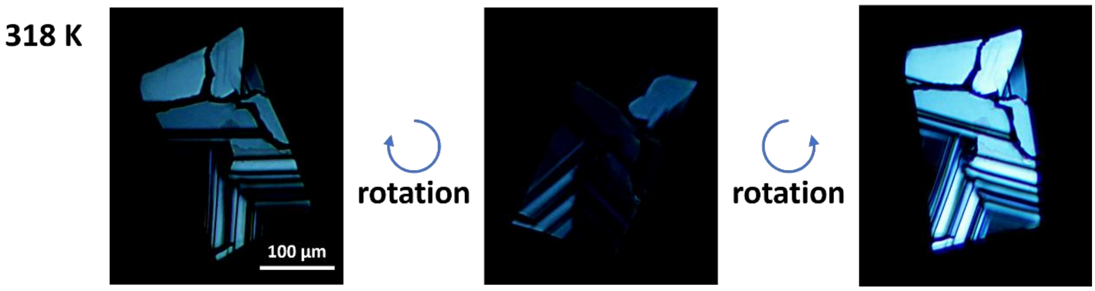

Figure S9. Evolution of ferroelastic domains during rotation.

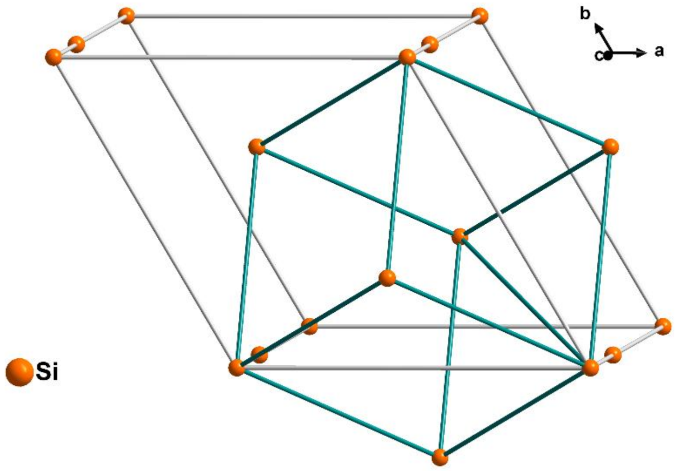

Figure S10. The lattice relationship between the basis vectors of the triclinic phase at 300 K (gray) and the trigonal phase at 373 K (green) of **1**.

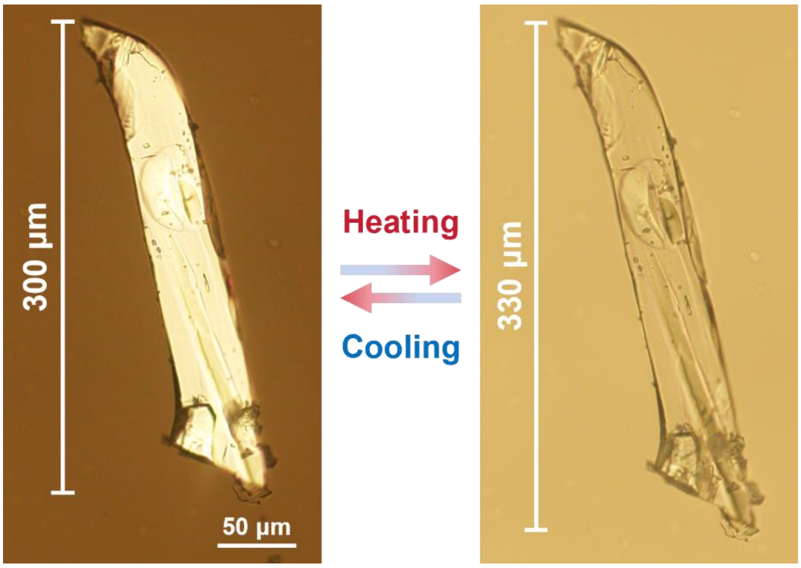

Figure S11. Optical images showing the reversible change in the length of the crystal of **1** during the phase transition.

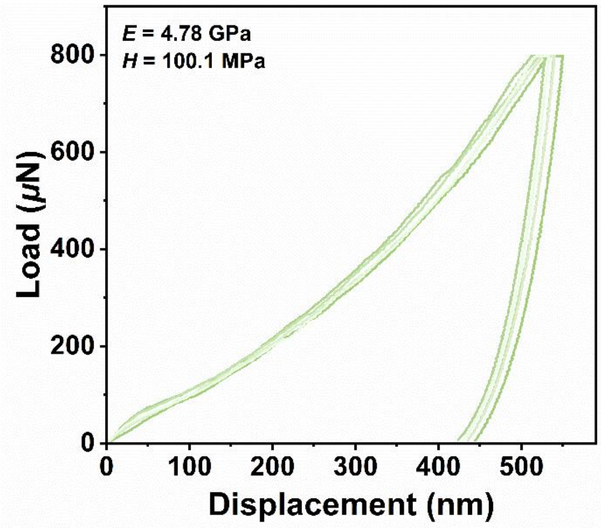

Figure S12. Load-displacement curves of the **1**.

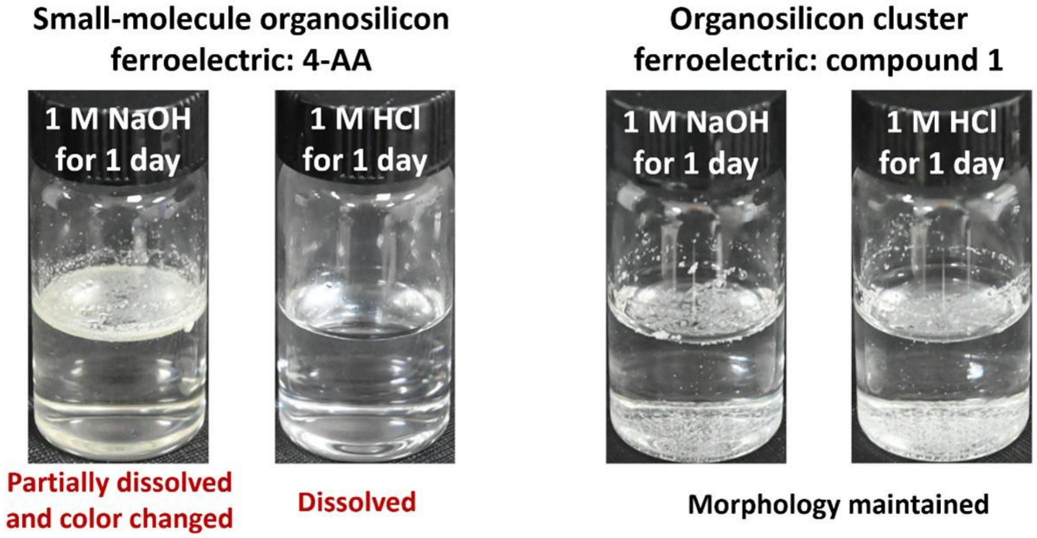

Figure S13. Morphology images of 4-AA (left) and compound **1**(right) after being immersed in aqueous HCl (1 M) and NaOH (1 M) solutions for 1 day.

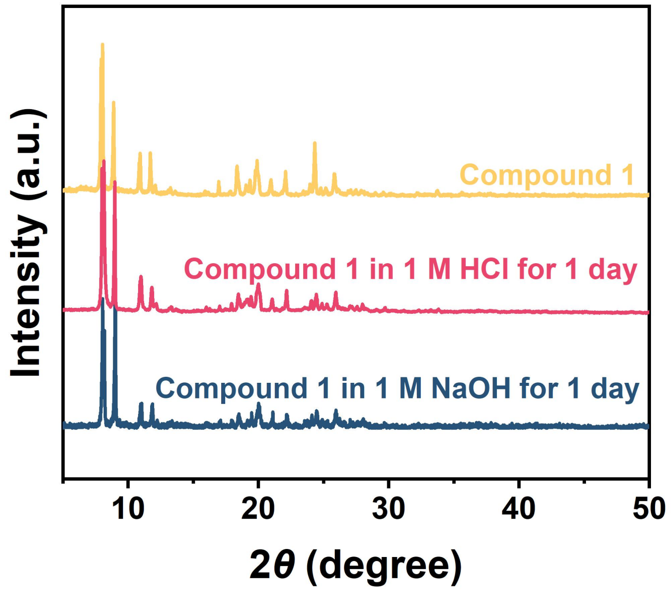

Figure S14. The corresponding PXRD spectra of compound **1** for both untreated and treated.

Table S1. Crystal data and structure refinement for compound **2** at 100, 300, and 370 K, respectively.

**Compound**	**2**	**2**	**2**
**Temperature**	100 K	300 K	370 K
**Formula**	$C_{32}H_{72}O_{12}Si_8$	$C_{32}H_{72}O_{12}Si_8$	$C_{32}H_{72}O_{12}Si_8$
**Weight**	873.61	873.61	873.61
**System**	triclinic	triclinic	trigonal
**Space group**	*P*1	*P*1	*R*3
a **(Å)**	9.9963(2)	10.12467(18)	16.2239(11)
b **(Å)**	10.8102(2)	11.14837(16)	16.2239(11)
c **(Å)**	10.9600(1)	11.19548(18)	17.2393(17)
$\boldsymbol{\alpha}$ **(°)**	96.770(1)	93.8033(12)	90
$\boldsymbol{\beta}$ **(°)**	91.073(1)	100.4056(14)	90
$\boldsymbol{\gamma}$ **(°)**	99.396(1)	90.6432(13)	120
**V (Å^3)**	1159.45(3)	1239.81(3)	3929.7(7)
Z	1	1	3
R_{int}	0.0218	0.0164	0.0417
R_1	0.0379	0.0515	0.2117
wR_2	0.1101	0.1626	0.2711
**GOF**	1.081	1.086	1.064

Table S2. Crystal data and structure refinement for compound **1** at 300 and 347 K, respectively.

**Compound**	**1**	**1**
**Temperature**	300 K	347 K
**Formula**	$C_{31}H_{71}O_{12}NSi_8$	$C_{31}H_{71}O_{12}NSi_8$
**Weight**	874.60	874.60
**System**	triclinic	trigonal
**Space group**	*P*1	*R*3
a **(Å)**	10.1134(2)	16.1357(12)
b **(Å)**	11.1379(1)	16.1357(12)
c **(Å)**	11.1621(1)	17.1112(17)
α **(°)**	93.844(1)	90
β **(°)**	100.347(1)	90
γ **(°)**	90.922(1)	120
**V (Å^3)**	1233.59(3)	3858.2(7)
Z	1	3
$\boldsymbol{R_{int}}$	0.0409	0.0174
$\boldsymbol{R_1}$	0.0828	0.1319
$\boldsymbol{wR_2}$	0.2838	0.2751
**GOF**	1.219	1.048
